# Comparative Skull Morphology of Uropeltid Snakes (Alethinophidia: Uropeltidae) with Special Reference to Disarticulated Elements and Variation

**DOI:** 10.1371/journal.pone.0032450

**Published:** 2012-03-08

**Authors:** Jennifer C. Olori, Christopher J. Bell

**Affiliations:** Department of Geological Sciences, Jackson School of Geosciences, The University of Texas at Austin, Austin, Texas, United States of America; Raymond M. Alf Museum of Paleontology, United States of America

## Abstract

Uropeltids form a diverse clade of highly derived, fossorial snakes that, because of their phylogenetic position among other alethinophidian lineages, may play a key role in understanding the early evolution of cranial morphology in snakes. We include detailed osteological descriptions of crania and mandibles for eight uropeltid species from three nominal genera (*Uropeltis*, *Rhinophis*, and *Brachyophidium*) and emphasize disarticulated elements and the impact of intraspecific variation on previously proposed morphological characters used for phylogenetic analysis. Preliminary analysis of phylogenetic relationships strongly supports a clade composed exclusively of species of *Plectrurus*, *Uropeltis*, and *Rhinophis*. However, monophyly of each of those genera and *Melanophidium* is not upheld. There is moderate support that Sri Lankan species (e.g., *Rhinophis* and *Uropeltis melanogaster*) are monophyletic with respect to Indian uropeltids. Previously proposed characters that are phylogenetically informative include the shape of the nasals, length of the occipital condyle, level of development of the posteroventral process of the dentary, and participation of the parietal in the optic foramen. Additionally, thirty new features that may be systematically informative are identified and described, but were not verified for their utility. Such verification must await availability of additional disarticulated cranial material from a larger sample of taxa. All characters require further testing through increased focus on sources and patterns of intraspecific variation, inclusion of broader taxonomic samples in comparative studies, and exploration of skeletal development, sexual dimorphism, and biogeographic patterns. Additionally, trends in the relative enlargement of the sensory capsules, reduction in cranial ossification and dentition, fusion of elements, and the appearance of novel morphological conditions, such as the structure and location of the suspensorium, may be related to fossoriality and miniaturization in some uropeltid taxa, and may complicate analysis of relationships within Uropeltidae and among alethinophidian snakes.

## Introduction

The Uropeltidae (*sensu* [1]) is a small group of fossorial snakes restricted to India and Sri Lanka, whose phylogenetic position among other snakes remains controversial. Based on morphological data, the clade was hypothesized to be the sister taxon to either *Anomochilus* [2]–[6] or *Anilius* ([7]; *Cylindrophis* and *Anomochilus* were not included), or along with *Cylindrophis*, *Anomochilus*, and *Anilius*, to be part of a series of successive outgroups to all other alethinophidian snakes [2], [5], [6]. Hypotheses based on molecular data are more variable, with uropeltids placed as the sister taxon to *Calabaria* [8], *Tropidophis* and *Casarea* [9], Caenophidia [10], *Liotyphlops* [7], *Cylindrophis* [6], [9], [11]–[13], or *Cylindrophis* and *Anomochilus* [14]. Superficially, the within-group relationships of uropeltids appear to be more stable and relatively robust over the last 150 years [15]. However, that stability results from a lack of re-evaluation of the validity and diagnoses of genera and species of uropeltids, coupled with limited analyses of higher-level relationships. The most recent taxonomic summary of the Uropeltidae recognized 47 species distributed within eight genera [1], but authorities on the group agree that major taxonomic revisions are required [1], [15], [16]–[19]. Phylogenetic analyses based on morphological [17], immunological [20], and genetic [21] data consistently indicated that currently recognized genera comprising more than one species are not monophyletic. Moreover, molecular analyses suggested that the Sri Lankan uropeltids constitute a monophyletic group [20], [21], which may necessitate re-assignment of species within the two most specious genera, *Uropeltis* and *Rhinophis* [22].

In addition to the relative paucity of recent systematic research on uropeltids, no developmental data are yet published, and patterns of interspecific variation remain undocumented. The majority of the scant literature on uropeltid biology that does exist is focused on species records including external measurements and scale counts (e.g., [23]–[30]). Descriptions rarely go below the surface, and few data on uropeltid anatomy were published between an early monograph on the skeleton of two species of *Rhinophis* [31] and a series of studies on the morphology of the ‘Henophidia,’ in which Uropeltidae was included [32]–[35]. The earliest studies of uropeltid anatomy were not as taxonomically comprehensive or anatomically detailed as those later works and included only limited illustrations and discussion of morphology [36]–[38]. Additionally, particular aspects of uropeltid cranial or functional anatomy were discussed in reference to specific taxa [19], [39]–[51] and a collection of studies specifically focused on the cranio-vertebral joint [52]–[54]. The two most comprehensive studies of cranial anatomy within the group provided reviews of uropeltid skull morphology and contributed new data and observations on available specimens in museum collections [17], [55]. Both works are particularly important because they included previously unstudied taxa. Nonetheless, small samples of individual taxa continue to hamper research on uropeltids. Skeletal collections of these snakes are limited, and in many cases single specimens serve as exemplars of entire (presumed) lineages.

Before his death in 2009, Carl Gans provided us with a remarkable collection of dried and previously unstudied uropeltid specimens that he collected in the 1970s. Those animals died shortly after capture and were desiccated for future preparation as skeletons. Unexpected dermestid beetle infestation at some point in the past resulted in partial skeletonization, and dissociation of some skeletal elements. Despite some damage, the collection provided us with an excellent opportunity to evaluate skeletal morphology and variation of multiple individuals of two species of uropeltids, and to comment on specimens of six additional species. In this paper we describe in detail the crania and mandibles of eight uropeltid species, using articulated, partially disarticulated, and fully disarticulated skulls. Our main objectives are to gain a deeper understanding of skull morphology in the group, assess the variation among individuals in each taxon, and compare skull morphology across the taxa represented in the sample. Previously proposed characters for phylogenetic analysis [17], [19] are evaluated in reference to the variation observed within our sample. In addition, we intend for this paper to be a starting point for the identification of new morphological characters that can be used in future phylogenetic analyses of this group as a whole, or lineages contained within it.

## Materials and Methods

During expeditions to India and Sri Lanka in the 1970s, Carl Gans and colleagues amassed one of the largest existing collections of uropeltid snakes. Many of those specimens were deposited at the California Academy of Sciences in San Francisco and are stored as standard alcohol-preserved specimens. Gans' collection included a subset of 48 dried specimens or partial specimens that originally were set aside for skeletal preparation; those were donated to the Texas Natural Science Center in Austin, Texas and are curated in the modern skeletal collection of the Vertebrate Paleontology Laboratory. Specimen numbers in that collection are designated as TMM M-# (e.g., TMM M-10022). At least 35 specimens retain partial skulls, and those form the material component of our analysis (Table 1). The cranial sample includes 15 specimens of *Brachyophidium rhodogaster*, 11 specimens of *Uropeltis woodmasoni*, two specimens each of *Rhinophis philippinus* and *Uropeltis melanogaster*, and one specimen each of *Uropeltis rubromaculata*, *Uropeltis* sp., *Rhinophis blythii*, *Rhinophis homolepis*, and *Rhinophis drummondhayi*. A total of seven skulls were partially disarticulated and free of soft tissue before any preparation began. We removed the head of each specimen; where possible, skulls were kept fully intact, soaked in warm water, and hand-cleaned with a needle under a dissecting microscope. Most of the soft tissues were destroyed by previous dermestid infestation, but scale samples were retained when available.

**Table 1:** Specimen data and measurements.

TMM number	current taxonomy	skull condition	skull length (mm)	narrowest width (mm)	greatest width (mm)	tooth count (U/L)
M-10001	*Uropeltis woodmasoni*	fully disarticulated			3	(8/10)
M-10002	*Uropeltis woodmasoni*	articulated	8.2	1.4	2.6	R-(6/10), L-(6/9)
M-10003	*Uropeltis woodmasoni*	articulated	9.2	1.4	3.2	(8/9)
M-10004	*Uropeltis woodmasoni*	articulated	9.6	1.45	3.2	(8/10)
M-10005	*Uropeltis woodmasoni*	articulated	7.9	1.25	2.8	R-(7/9), L-(8/9)*
M-10006	*Uropeltis woodmasoni*	articulated	9.4	1.6	3.3	(8/10)
M-10007	*Uropeltis woodmasoni*	articulated	9.5	1.4	3.3	(8/10)
M-10008	*Uropeltis woodmasoni*	articulated	9.2	1.4	3.2	(8/10)
M-10009	*Uropeltis woodmasoni*	articulated	8	1.3	2.7	(8/10?)
M-10010	*Uropeltis woodmasoni*	partially disarticulated	7.8	1.3	2.75	(8/9)
M-10011	*Brachyophidium rhodogaster*	articulated	7.3	1.1	2.9	(9/12?)
M-10013	*Brachyophidium rhodogaster*	partially disarticulated	6.4∧	1.1	3	(9/10)
M-10014	*Brachyophidium rhodogaster*	articulated	7.9	1.15	3	(9/10)
M-10015	*Brachyophidium rhodogaster*	articulated	7	1	2.9	(9/10)
M-10016	*Brachyophidium rhodogaster*	partially disarticulated	6.1∧	1.1	3	(9/10)
M-10017	*Brachyophidium rhodogaster*	articulated	7	1	2.9	R-(9/9), L-(9/10)
M-10018	*Brachyophidium rhodogaster*	partially disarticulated	7.5	1.1	2.9	(9/10)
M-10019	*Brachyophidium rhodogaster*	partially disarticulated			3	(9/10)
M-10020	*Brachyophidium rhodogaster*	articulated	7.8	1.1	3	(9/10)
M-10021	*Uropeltis woodmasoni*	fully disarticulated			3.1	(8/?)
M-10022	*Brachyophidium rhodogaster*	fully disarticulated			3.1	(9/10)
M-10023	*Brachyophidium rhodogaster*	fully disarticulated			3.1	(9/10)
M-10024	*Brachyophidium rhodogaster*	fully disarticulated			2.7	(9/?)
M-10025	*Brachyophidium rhodogaster* **	fully disarticulated			2.65	R-(8/9), L-(8/10?)
M-10026	*Brachyophidium rhodogaster*	fully disarticulated			2.9	(9/10)
M-10027	*Brachyophidium rhodogaster*	fully disarticulated			2.9	(?/?)
M-10028	*Uropeltis rubromaculata*	articulated	9.4	1.4	3.4	(5?/8)
M-10030	*Rhinophis blythii*	articulated	10	1.65	3.65	(7/8)
M-10032	*Uropeltis melanogaster*	fully disarticulated			2.4	R-(7?/8), L-(6/8)
M-10036	*Uropeltis sp.*	partially disarticulated	5.7∧	1.1	2.5	R-(8?/7), L-(7-8?/7)
M-10037	*Rhinophis philippinus (?)*	partially disarticulated	7.2	1.2	2.4	(5/7)
M-10038	*Rhinophis philippinus*	partially disarticulated	6.4∧	1.1	2.2	(5/7)
M-10041	*Rhinophis homolepis*	articulated	6.2	1.1	2.4	(7/9?)
M-10045	*Uropeltis melanogaster*	fully disarticulated			2.9	(7/8)

Current taxonomy from [1]. U = upper jaw; L = lower jaw.

* dentary is shorter on right side (asymmetry).

∧ parietal separated from braincase, measured across otic capsules.

** Misidentified - probably *Uropeltis* sp.

Three specimens were at least partially disarticulated originally, and two additional specimens were fully disarticulated during preparation; the latter two were selected for disarticulation because they were already partially dissociated. We removed the lower jaws and quadrate from at least one side of the head of many specimens, but ‘partially disarticulated’ in Table 1 and Table S1 refers to removal of other elements in addition to those.

Before any preparation was undertaken, a single specimen of *Uropeltis woodmasoni* (TMM M-10006) was CT scanned at the University of Texas at Austin High Resolution X-ray Computed Tomography Laboratory. The full dataset is available at www.Digimorph.org. A total of 1175 coronal (axial) slices were acquired using a field of reconstruction of 14 mm (maximum field of view 14.09 mm) and 25 slices per rotation. Both the slice thickness and the inter-slice spacing of the raw CT slices are 0.01495 mm and the image resolution is 1024×1024. The volume graphics software VGStudioMax (version 2.0.1., 2008, Volume Graphics, Heidelberg, Germany) was used for image processing, including reslicing and 3-D reconstruction. Additionally, all cranial elements were rendered separately using the segmentation tool of that software package in order to digitally disarticulate the skull.

We used *U. woodmasoni* as the basis for a general description of each element; for other taxa we discuss only the differences from the condition seen in *U. woodmasoni*. A list of specimens, current taxonomy (follows [1]), preservation condition, measurements of each skull, and tooth counts are given in Table 1. Locality data, field numbers, original taxonomic identifications as provided by C. Gans, and expanded comments on skull condition are found in Table S1. Note in particular that specimens called *Teretrurus rhodogaster* by C. Gans are referred to as *Brachyophidium rhodogaster* by us (following [1]). Additionally, *Rhinophis trevelyanus*, the original identification for specimen TMM M-10041, is considered a junior synonym of *Rhinophis homolepis* [1]. All measurements were taken in dorsal view. Skull length is a linear measurement from the tip of the premaxilla to the posterior tip of the occipital condyle, ‘narrowest width’ is a linear measurement of the width across the midpoint of the frontals, and ‘greatest width’ is the width across the otic capsules at the level of the juxtastapedial recess. In our osteological descriptions the term ‘foramen’ refers to an opening enclosed within a single bone, whereas a ‘fenestra’ is an opening bounded by multiple elements [56].

In order to evaluate morphological characters proposed previously for phylogenetic analysis [17], all TMM-M specimens referred to *Rhinophis*, *U. melanogaster*, and *U. rubromaculata* were scored for those original 33 characters (see Table S2). Nine specimens each of the TMM-M *U. woodmasoni* and *B. rhodogaster* (completely disarticulated individuals not scored), and a previously CT-scanned specimen of *Plectrurus aureus* from the California Academy of Science (CAS 17177) [19] were additionally scored and included. The specimens of uropeltids from the Natural History Museum, London (BMNH), upon which the original character descriptions were based [17], also were examined and re-scored in person (JCO), except for the specimen of ‘*Teretrurus rhodogaster*’ (BMNH 1930.5.8.98), which was unavailable. Scores for the original outgroup taxa were checked using skeletal specimens from the University of California Museum of Paleontology (*Cylindrophis rufus*, UCMP 136995) and CT data (*Anomochilus* and *Anilius*) provided to us by the Squamate Tree of Life project (Deep Scaly). In some cases the original character descriptions [17] were modified or expanded by us, and in most cases the descriptions were annotated based on our osteological and literature review (see Character Descriptions in Methods S1).

The redundancy of the Operational Taxonomic Units in the matrix providing the full range of intraspecific variation (Table S2) was reduced to obtain a matrix (see Methods S2, S3) for phylogenetic analysis of 16 species of uropeltids. In cases where specimens of the same species were scored for BMNH and TMM specimens, both sets were retained in the matrix to evaluate whether they would form a clade. Specimens from BMNH posed problems for scoring because many individuals are articulated, soft tissue is present, and historical names are retained despite subsequent taxonomic revisions (the latter is a complex problem because many specimens are historical holotypes or potentially are misidentified). Additionally, without examining the specimen originally called ‘*Teretrurus rhodogaster*’ by [17], we were unsure if that individual is the same taxon as our *B. rhodogaster* or if it is *Teretrurus sanguineus*. The phylogenetic analysis was run in PAUP* [57] using parameters similar to those published previously for analysis of uropeltid morphological characters [17]. All characters were treated as unordered and unweighted, branches were collapsed if the minimum branch lengths were zero (amb-), and DELTRAN was used for character optimization. Because our study documented a wider range of intraspecific variation than recognized previously, polymorphism was distinguished from uncertainty in multistate taxa (setting = ‘respect () verses {}’). The analysis was run under maximum parsimony as a heuristic search using random addition, Tree Bisection Reconnection, and 1000 replicates, and the resulting trees were rooted using the outgroup taxa *Anilius*, *Anomochilus*, and *Cylindrophis* (as in original analysis by [17]). Bremer support [58] for nodes retained in the Strict Consensus tree was calculated manually in PAUP* using constraint trees generated in MacClade 4.08 for OS X [59] from the Decay Index PAUP* File command.

## Results

### Premaxilla

The premaxilla is the anterior-most element in the head and tapers anteriorly in all uropeltids, terminating in a gentle dorsoventral curvature. The bone is posteriorly tripartite, with distinct nasal, transverse, and vomerine processes. In all species we examined, the premaxilla contacts the maxilla posterolaterally, the nasal posterodorsally, and the septomaxilla and vomer posteriorly. A single, ventral premaxillary foramen occurs at (or near) the midline in all specimens we examined. The premaxilla is edentulous.

#### *Uropeltis woodmasoni*

**Figure 1:**
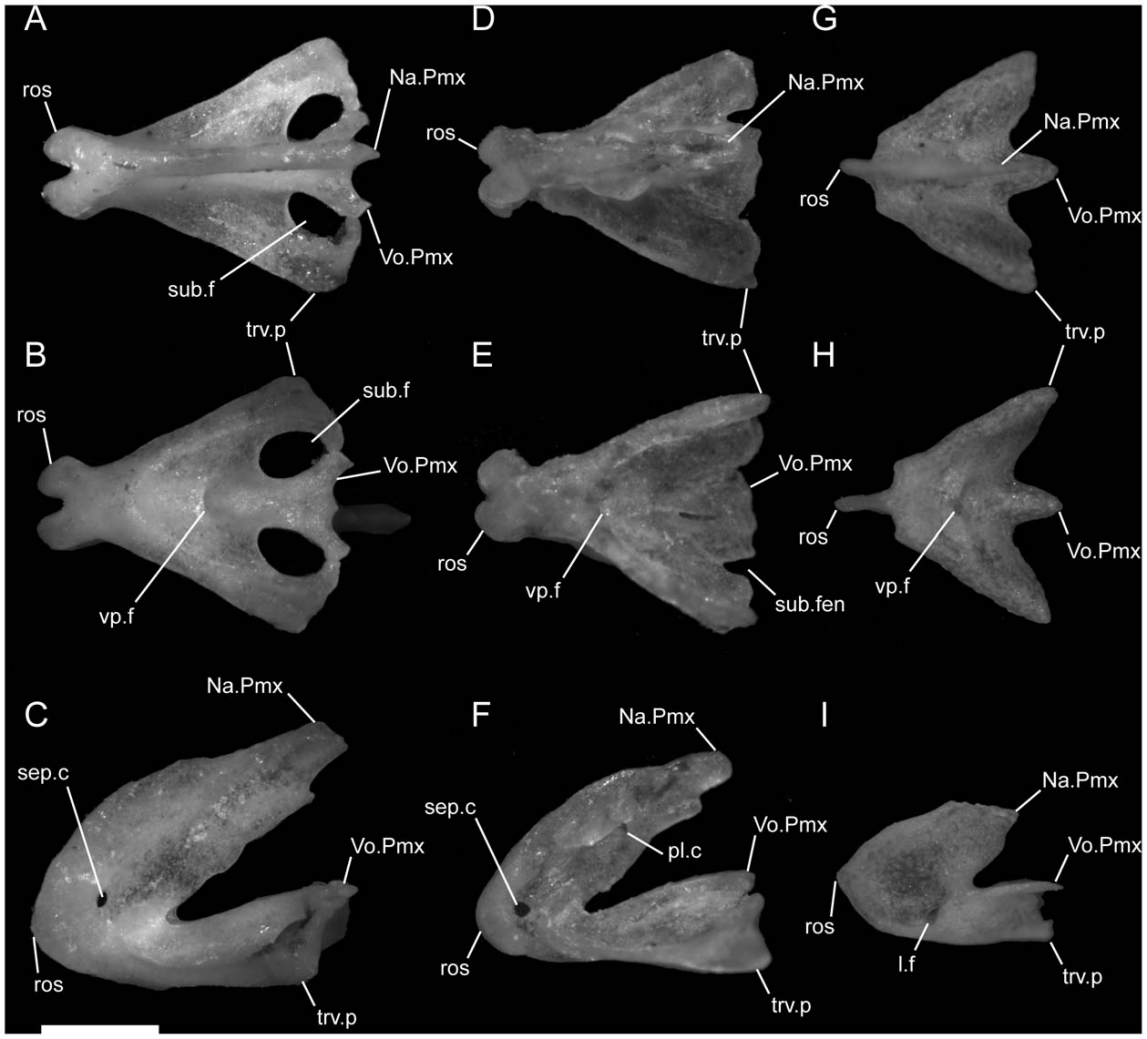
Disarticulated premaxillae. Anterior is to the left; scale bar = 0.5 mm. A–C from *U. woodmasoni* (TMM M-10001); D–F from *U. melanogaster* (TMM M-10045); and G–I from *B. rhodogaster* (TMM M-10024). A,D,G in dorsal view; B,E,H in ventral view; and C,F,I in lateral view. The posterior tip of the nasal process is broken in the *B. rhodogaster* specimen. l.f = lateral foramen; Na.Pmx = nasal process (keel) of premaxilla; pl.c = posterolateral canal; ros = rostral tip; sep.c = septal canal; sub.f = subnarial foramen; sub.fen = subnarial fenestra; trv.p = transverse process; vp.f = ventral premaxillary foramen; Vo.Pmx = vomerine process of premaxilla.

**Figure 2:**
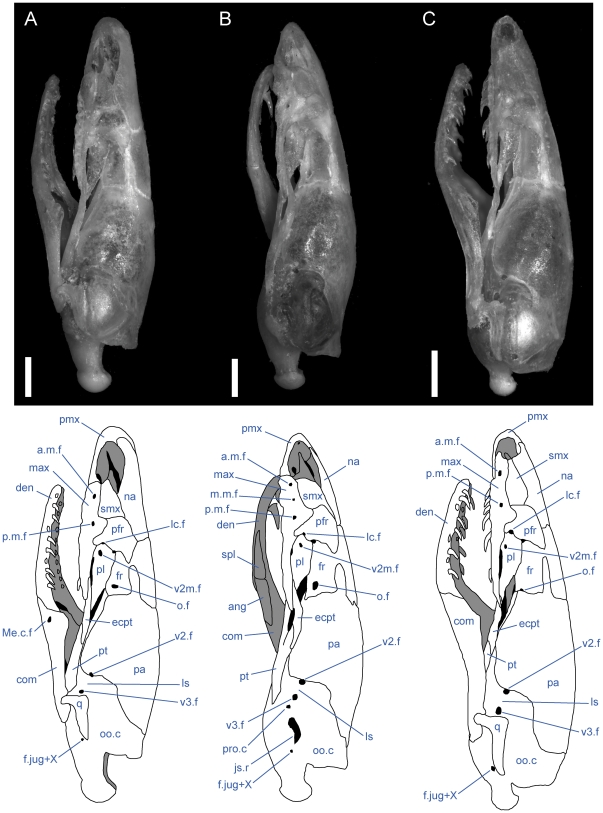
Left lateral view of articulated *Uropeltis* and *Brachyophidium* skulls. Anterior up; scale bars = 1.0 mm. (A) *U. woodmasoni*, TMM M-10006; (B) *U. rubromaculata*, TMM M-10028; (C) *B. rhodogaster*, TMM M-10011. a.m.f. = anterior maxillary foramen; Ang = angular; com = compound; den = dentary; ecpt = ectopterygoid; fr = frontal; js.r = juxtastapedial recess; lc.f = lacrimal foramen; ls = laterosphenoid region; m.m.f = middle maxillary foramen; max = maxilla; Me.c.f = foramen associated with canal for Meckel's cartilage; na = nasal; o.f = optic foramen; oo.c = otooccipital complex; p.m.f = posterior maxillary foramen; pa = parietal; pfr = prefrontal; pl = palatine; pmx = premaxilla; pro.c = prootic canal; pt = pterygoid; smx = septomaxilla; spl = splenial; q = quadrate; v2.f = foramen for maxillary branch of trigeminal nerve; v2m.f = foramen for branch of maxillary branch of trigeminal nerve; v3.f = foramen for mandibular branch of trigeminal nerve.

The rounded anterior tip of the rostrum possesses a midline groove that extends along the entire anterior surface of the bone, giving it a bifurcate appearance when viewed dorsally (the ‘bipartite-rostrum’ of [17]; Fig. 1A). This groove or notch varies individually in its depth but is universally present and distinct in our sample of *U. woodmasoni*. The groove shallows dorsally and disappears as the nasal process extends posteriorly to separate the nasals for approximately the anterior third of their length. The dorsal exposure of the nasal process is narrow, tapering posteriorly as it is overlapped dorsally by the horizontal laminae of the nasals. The nasal process is robust and dorsoventrally extensive, but this is best seen in the disarticulated element (Fig. 1C). In lateral view of the articulated skull, the lateral wall of the process is visible as the medial wall of the external naris (Fig. 2A). The lateral wall of the process is generally smooth and unornamented, with a barely perceptible shallow channel oriented anteroventral-posterodorsal, along the central section of the process. Posteroventrally the nasal process meets the transverse process laterally and the vomerine process along the midline. Just dorsal to that junction, a tiny, anteriorly directed canal pierces the base of the nasal process posteriorly. In at least some specimens (e.g., TMM M-10001), that canal bifurcates after traveling a short distance, with separate rami passing anterolaterally towards the edge of the snout (this can only be seen in an immaculately clean disarticulated element). In addition, the lateral surface of the base of the nasal process is pierced by a small canal in TMM M-10001 and TMM M-10002 (i.e., septal foramen, Fig. 1C); in one other specimen (TMM M-10006) a single foramen pierces the median septum on only the left side, but a canal is not formed.

**Figure 3:**
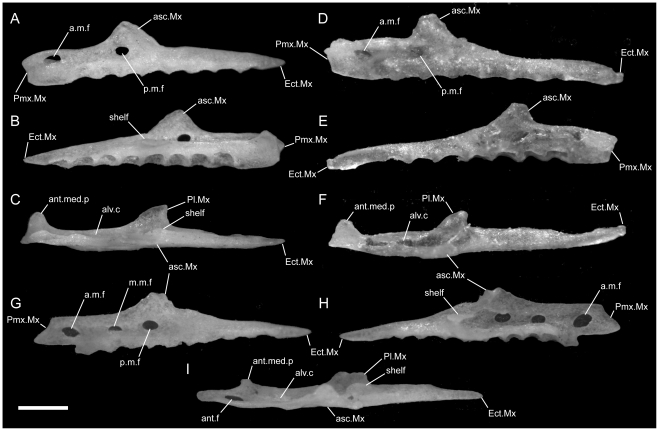
Disarticulated maxillae. Anterior is to the left unless noted; scale bar = 0.5 mm. All elements from the left side of the skull. A–C from *U. woodmasoni* (TMM M-10001); D–F from *U. melanogaster* (TMM M-10045); and G–I from *B. rhodogaster* (TMM M-10022). A,D,G in lateral view; B,E,H in medial view (anterior is to the right); and C,F,I in dorsal view. a.m.f = anterior maxillary foramen; alv.c = alveolar canal; ant.med.p = anteromedial process; asc.Mx = ascending process; Ect.Mx = ectopterygoid process of maxilla; f.jug+X = foramen for jugular vein and vagus nerve; m.m.f = middle maxillary foramen; p.m.f = posterior maxillary foramen; Pl.Mx = palatine process (posteromedial process) of maxilla; Pmx.Mx = premaxillary process of maxilla; shelf = shelf medial to articulation facet for prefrontal.

The transverse process of the premaxilla forms the ventral margin of the external nares and contacts the maxilla posteriorly in lateral view. The contact between the premaxilla and maxilla was described previously as ‘schizarthrotic’ [17], a term intended to describe the generally flat and buttressing contact visible in lateral view. The contact usually is more or less vertical and does not involve true clasping or overlap by either element, but the detailed nature of the contact varies individually, including variations generated by slight curvature of the posterior end of the transverse process, the anterior end of the maxilla, or both (Figs. 1C, 2A, 3A). Posteriorly, a small medial process turns towards the midline and forms the posterolateral edge of a relatively large subnarial opening (a similar process in *Pseudotyphlops philippinus* was reported previously [17], [55]). In most of our specimens of *U. woodmasoni* that process is well developed and meets a posterolaterally oriented flange of the vomerine process to enclose the foramen completely within the premaxilla (Fig. 1B). The exceptions are TMM M-10007 and TMM M-10010, in which the foramen is fully enclosed within the premaxilla on the left side, but on the right the palatal tubercle of the septomaxilla forms the posterior margin.

**Figure 4:**
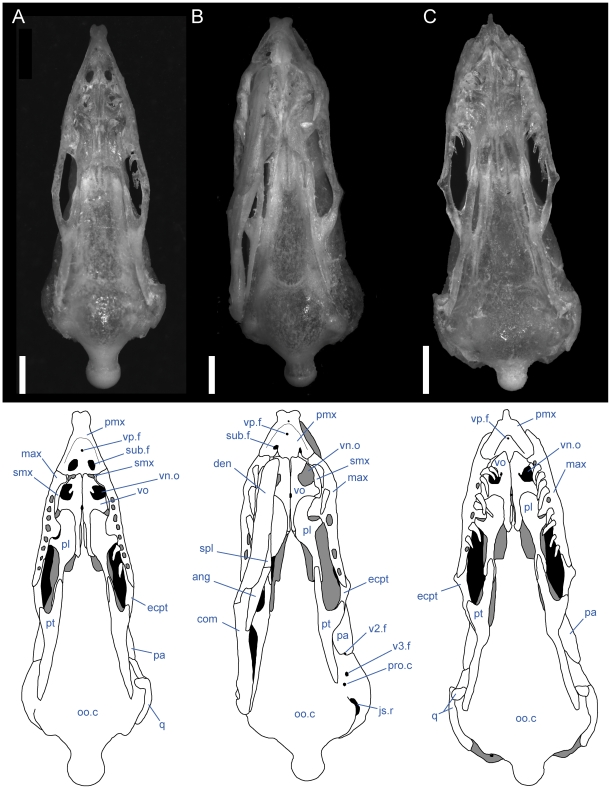
Ventral view of articulated *Uropeltis* and *Brachyophidium* skulls. Anterior up; scale bars = 1.0 mm. (A) *U. woodmasoni*, TMM M-10006; (B) *U. rubromaculata*, TMM M-10028; (C) *B. rhodogaster*, TMM M-10011. Ang = angular; com = compound; den = dentary; ecpt = ectopterygoid; js.r = juxtastapedial recess; max = maxilla; oo.c = otooccipital complex; pa = parietal; pl = palatine; pmx = premaxilla; pro.c = prootic canal; pt = pterygoid; smx = septomaxilla; spl = splenial; sub.f = subnarial foramen; q = quadrate; vn.o = vomeronasal opening; vo = vomer; vp.f = ventral premaxillary foramen; v2.f = foramen for maxillary branch of trigeminal nerve; v3.f = foramen for mandibular branch of trigeminal nerve.

The vomerine process of the premaxilla is a ventral midline projection that extends posteriorly to abut the vomers. In the articulated skull, the two elements meet in what appears as a tightly abutting articulation (Fig. 4A). That articulation masks a triangular dorsomedial projection on the vomer that overrides the premaxilla. Posteriorly, the vomerine process forms a minute midline projection, on either side of which are two crescentic emarginations that form a w-shaped articulation surface with the vomer in ventral view (Figs. 2B; 4A). A short posterolateral flange forms the posteromedial margin of the subnarial foramen. That flange contacts the medial inflection of the transverse process to fully enclose the foramen in most specimens (see above). Although not visible in the articulated skull, the septomaxilla also contacts the vomerine process dorsally. The function of the subnarial foramen is unknown, and that opening was not previously discussed in the literature.

Just anterior to the subnarial foramina, the ventral surface of the premaxilla is excavated into a dorsally convex recess (Fig. 1B). At its anterior margin the ventral premaxillary foramen [17] marks the posterior end of a canal that penetrates anteriorly into the body of the premaxilla.

#### *Uropeltis rubromaculata*

**Figure 5:**
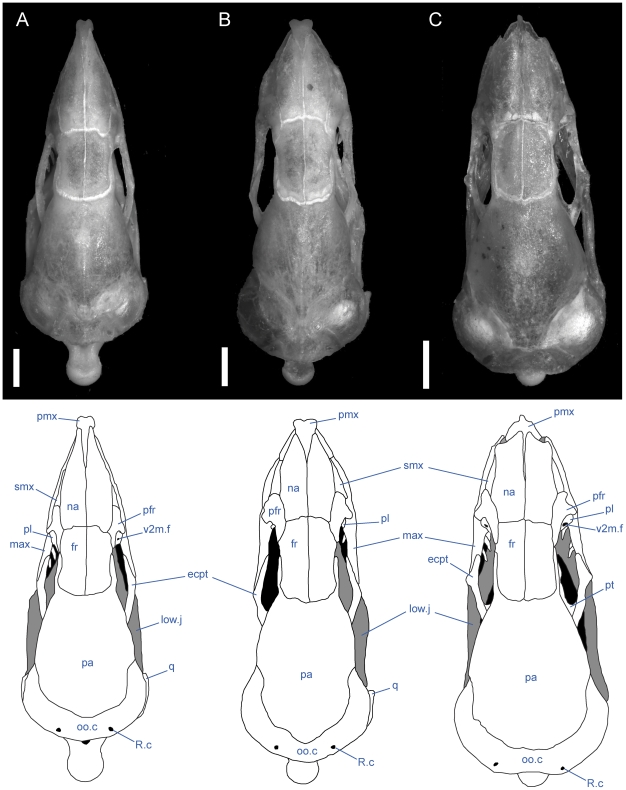
Dorsal view of articulated *Uropeltis* and *Brachyophidium* skulls. Anterior up; scale bars = 1.0 mm. (A) *U. woodmasoni*, TMM M-10006; (B) *U. rubromaculata*, TMM M-10028 (C) *B. rhodogaster*, TMM M-10011. Ecpt = ectopterygoid; fr = frontal; low.j = lower jaw; max = maxilla; na = nasal; oo.c = otooccipital complex; pa = parietal; pfr = prefrontal; pmx = premaxilla; pl = palatine; pt = pterygoid; smx = septomaxilla; q = quadrate; R.c = Rieppel's canal; v2m.f = foramen for branch of maxillary branch of trigeminal nerve.

The anterior rostrum is broader than that of *U. woodmasoni*, as are the sagittal groove and the portion of the nasal process that separates the nasals (Fig. 5B). Contact with the vomers does not differ substantially from *U. woodmasoni*. The subnarial foramen is enclosed entirely within the premaxilla on the right side, but on the left the posterior margin is formed by the palatal tubercle of the septomaxilla (Fig. 4B). The contact with the maxilla is relatively broader than in *U. woodmasoni*, owing mostly to the proportionally broader transverse process of the premaxilla. In palatal view, the vomer is excluded from contact with the maxilla and the transverse process of the premaxilla by a significant exposure of the septomaxilla. A mediolaterally oriented canal penetrates the septum at the base of the nasal process (as in some specimens of *U. woodmasoni*). The ventral premaxillary foramen is formed as in *U. woodmasoni*, but an additional small foramen is situated anterior to the former foramen, entering dorsally into the body of the rostrum.

#### *Uropeltis melanogaster*

Both of our specimens (TMM M-10032 and TMM M-10045) are completely disarticulated, so we are unable to comment on the specific nature of most contacts. However, a small, curved, anteroposteriorly directed shelf sits on either side of and ventral to the dorsal exposure of the nasal process (Fig. 1D,F). The shelf widens laterally and is expanded for approximately the middle third of the length of the dorsal exposure. These shelves are clearly articulation surfaces for the nasals, and the surfaces extend along the lateral surface of the nasal process, converging posteriorly at a point well posterior to the portion of the process that would be externally exposed in the articulated skull. On the right side of TMM M-10045, a narrow flange of bone extends ventrally from the lateral edge of the anterior portion of the shelf to contact a short flange of bone rising dorsally from the base of the midline septum, forming a short canal just posterior to the rostrum. On the left side of the same specimen a similar canal is formed posteriorly, at approximately the level of the posterior edge of the widest part of the shelf (Fig. 1F). In TMM M-10032, posterior canals are formed on both sides; anteriorly the two flanges are present but fail to meet. This creates the appearance of a deep, anteroposteriorly oriented channel in lateral view. Based on the shallow w-shaped posterior margin of the vomerine process (Fig. 1E), the premaxilla probably contacted the vomers in a similar manner to that of *U. woodmasoni*, with the vomer slightly overlapping the premaxilla internally. The subnarial fenestra is open posteriorly, but we are unable to determine if its posterior margin was formed by vomer, septomaxilla, maxilla, or some combination of those. Additionally, TMM M-10045 possesses a small, enclosed slit along the midline of the vomerine process, anterior to the articulation area with the vomer; the slit makes it appear as though the vomerine process represents an incomplete fusion of two processes (Fig. 1E). The anteriorly directed canal where the base of the nasal process meets the vomerine process is also present in *U. melanogaster*. A secondary, anterior, ventral premaxillary foramen is present in TMM M-10032, but is larger than that seen in *U. rubromaculata*. The mediolaterally oriented canal piercing the base of the septum of the nasal process (Fig. 1F) is relatively larger in *U. melanogaster* than it is in *U. woodmasoni*.

#### *Rhinophis blythii*

**Figure 6:**
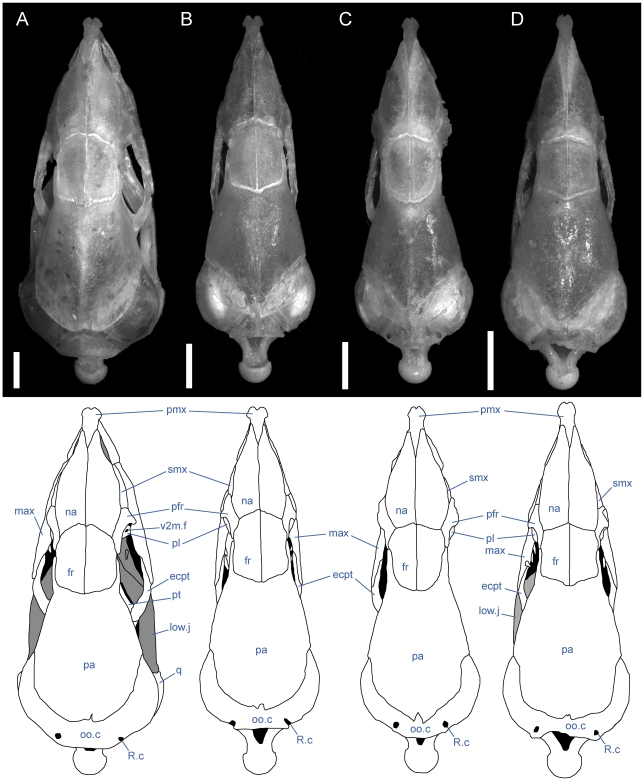
Dorsal view of articulated *Rhinophis* skulls. Anterior up; scale bars = 1.0 mm. (A) *R. blythii*, TMM M-10030; (B) *R. drummondhayi*, TMM M-10046; (C) *R. philippinus*, TMM M-10037; (D) *R. homolepis*, TMM M-10041. Ecpt = ectopterygoid; fr = frontal; low.j = lower jaw; max = maxilla; na = nasal; oo.c = otooccipital complex; pa = parietal; pfr = prefrontal; pl = palatine; pmx = premaxilla; pt = pterygoid; smx = septomaxilla; q = quadrate; R.c = Rieppel's canal; v2m.f = foramen for branch of maxillary branch of trigeminal nerve.

**Figure 7:**
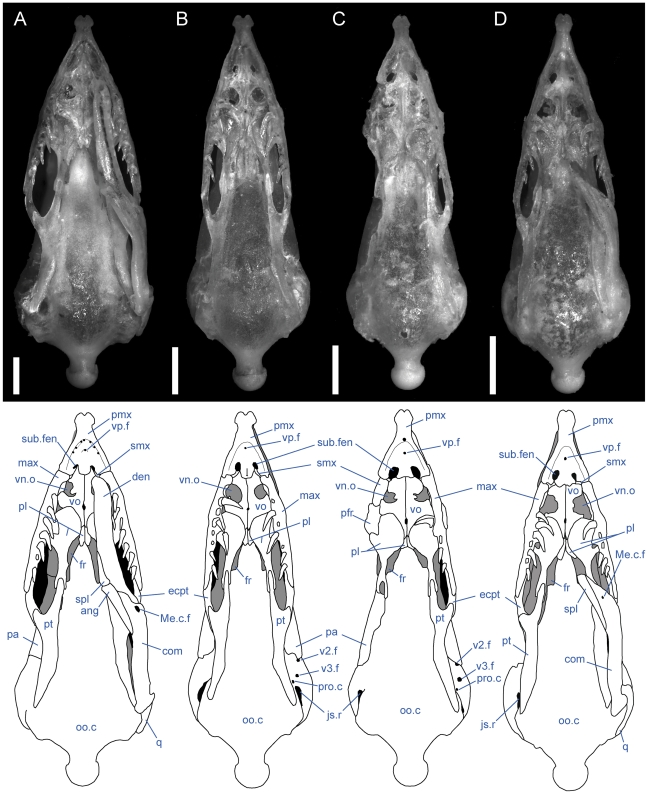
Ventral view of articulated *Rhinophis* skulls. Anterior up; scale bars = 1.0 mm. (A) *R. blythii*, TMM M-10030; (B) *R. drummondhayi*, TMM M-10046; (C) *R. philippinus*, TMM M-10037; (D) *R. homolepis*, TMM M-10041. Ang = angular; com = compound; den = dentary; ecpt = ectopterygoid; fr = frontal; js.r = juxtastapedial recess; max = maxilla; oo.c = otooccipital complex; pa = parietal; pfr = prefrontal; pl = palatine; pmx = premaxilla; pro.c = prootic canal; pt = pterygoid; smx = septomaxilla; spl = splenial; sub.fen = subnarial fenestra; q = quadrate; vn.o = vomeronasal opening; vo = vomer; vp.f = ventral premaxillary foramen; v2.f = foramen for maxillary branch of trigeminal nerve; v3.f = foramen for mandibular branch of trigeminal nerve.

**Figure 8:**
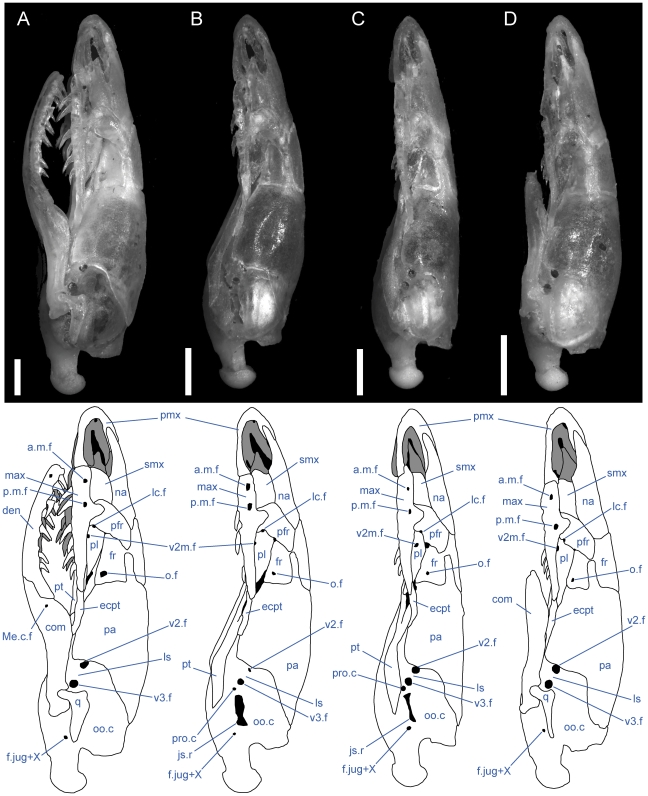
Left lateral view of articulated *Rhinophis* skulls. Anterior up; scale bars = 1.0 mm. (A) *R. blythii*, TMM M-10030; (B) *R. drummondhayi*, TMM M-10046; (C) *R. philippinus*, TMM M-10037; (D) *R. homolepis*, TMM M-10041. a.m.f. = anterior maxillary foramen; com = compound; den = dentary; ecpt = ectopterygoid; fr = frontal; js.r = juxtastapedial recess; lc.f = lacrimal foramen; ls = laterosphenoid region; m.m.f = middle maxillary foramen; max = maxilla; Me.c.f = foramen associated with canal for Meckel's cartilage; na = nasal; o.f = optic foramen; oo.c = otooccipital complex; p.m.f = posterior maxillary foramen; pa = parietal; pfr = prefrontal; pl = palatine; pmx = premaxilla; pro.c = prootic canal; pt = pterygoid; smx = septomaxilla; q = quadrate; v2.f = foramen for maxillary branch of trigeminal nerve; v2m.f = foramen for branch of maxillary branch of trigeminal nerve; v3.f = foramen for mandibular branch of trigeminal nerve.

The midline sagittal groove on the rostrum is deep. The portions of the bone lateral to this groove are more inflated than in *Uropeltis* and flare out laterally for a short distance along the bone's length before the margin turns sharply to the midline, creating a clear separation between the rostral tip and the main body of the premaxilla (Fig. 6A). The dorsal exposure of the nasal process in *R. blythii* is broad anteriorly and free of contact with the nasals for almost half of the length of the process posterior to the flared rostrum. The single specimen we examined (TMM M-10030) possessed a foramen piercing the median septum, immediately posterior to the rostral swelling. Immediately posterior to that foramen, the base of the nasal process swells laterally forming the lower margin of a channel that traverses the lateral wall of the nasal process (as in *U. melanogaster*); in *R. blythii* that channel appears to empty anteriorly into the foramen piercing the median septum. Posterodorsally, the channel margins are well formed (dorsally by lateral articulation facets for the nasals), but not enclosed in canals. A midline ventral premaxillary foramen pierces the floor of the premaxilla. Anterolateral to the foramen, three additional foramina are formed. The two on the right form a short canal, but only a single opening occurs on the left (Fig. 7A). These foramina are separated by a shallow groove from elongated canals that traverse the ventral portion of the transverse process. In our specimen, those canals are filled with darkened soft tissue and are, thus, clearly visible through the bone. The vomerine process is broad and squared posteriorly, apparently lacking the posterior emarginations seen in the three *Uropeltis* species. The contact between the vomer and the premaxilla is more complex, as well. Dorsally, the vomer extends over the vomerine process of the premaxilla in a manner similar to that in *U. woodmasoni*. In ventral view the vomer tightly abuts the premaxilla along the midline (as in the *Uropeltis* species), but in *R. blythii*, the premaxillary process of the vomer extends anteriorly along the lateral side of the vomerine process of the premaxilla to contribute to the posterior and posteromedial margins of the subnarial fenestra. In lateral view, the transverse process meets the maxilla in a slightly sinuous contact (Fig. 8A).

#### *Rhinophis drummondhayi*

With few exceptions, the premaxilla is similar to that of *R. blythii*. The rostral tip possesses steeper sides, forming a sharper separation of the tip from the main body in dorsal view (Fig. 6B). The subnarial fenestrae are relatively rounder and larger, and are almost fully enclosed by premaxilla ossification; only a narrow portion of the posterior margin is closed by septomaxilla (Fig. 7B). The vomerine process has a shallow W-shaped posterior emargination and is open along a narrow slit along the posterior midline. As in *R. blythii*, short anterior projections of the premaxillary processes of the vomer bound the vomerine process of the premaxilla laterally.

#### *Rhinophis philippinus*

The ventral premaxillary foramen occurs on the midline, but the canal into which it opens travels only a short distance anteriorly before its floor is pierced by a large ventral foramen, clearly visible in ventral view (Fig. 7C). The subnarial fenestra is closed posteriorly by the vomer, and possibly a small contribution from the septomaxilla.

#### *Rhinophis homolepis*

The premaxilla is similar to that of *R. blythii*, but the rostrum is more rounded and less broad (similar to the condition in *U. woodmasoni*). The contact with the maxilla in lateral view is almost vertical, but a small posterodorsal tip of the transverse process of the premaxilla overlaps the anterior portion of the dorsal margin of the maxilla (Fig. 8D). The vomerine process is emarginated in a way similar to that of *R. drummondhayi*. The subnarial opening is completely enclosed by the premaxilla on the right side, but a small portion of the septomaxilla closes the opening on the left (Fig. 7D). A single ventral premaxillary foramen and a foramen piercing the medial septum are present.

#### *Brachyophidium rhodogaster*

The premaxilla is delicately built, and its contacts differ substantially from those in the species of *Uropeltis* and *Rhinophis*. The premaxilla lacks the swollen rostrum and anterior sagittal groove. The nasal process is a thin, vertical sheet, and its dorsal exposure is greatly reduced (Fig. 5C). The lateral walls are smooth, with no obvious channel or groove, and taper posteriorly to a triangular point (Fig. 1I). No mediolaterally oriented foramen pierces the septum. The transverse process is triangular, with the apex pointed posterolaterally (Fig. 1H). It is shallowly concave dorsally along its lateral margin, and its posteromedial portion slopes dorsally to meet the vomerine process. The transverse process contacts the maxilla posteriorly, but instead of the abutting articulation seen in other taxa, in *B. rhodogaster* a thin lamina of premaxilla is visible ventral to the maxilla (Fig. 2C). The vomerine process is relatively short and tapers to a rounded triangular tip posteriorly (Fig. 1H). The lateral edges of the vomerine process are underlapped by the ventral premaxillary processes of the vomers. The dorsal premaxillary process of the vomer extends a slight distance over the main body of the premaxilla on either side of the vomerine process, so that the latter is clasped by the vomer. There is no subnarial opening. Ventrally, where the vomerine process meets the transverse and nasal processes, the single midline ventral premaxillary foramen is well developed and large (Fig. 1H). It marks the posterior opening of a short canal. Anterodorsally the canal opens at the base of the nasal process; that process bisects the opening, and a foramen is visible in lateral view (Fig. 1I). Minute additional ventral foramina open anterolateral to the premaxillary foramen in a clean, disarticulated specimen (TMM M-10024). In articulated skulls, these may be difficult to see, but one is visible on the right in TMM M-10017. Relatively large, unpaired foramina are developed in the same position on the left side in TMM M-10013 and TMM M-10019; a single relatively large foramen occurs on the right in TMM M-10020.

### Maxilla

In all taxa studied, the maxilla contacts the premaxilla anteriorly, the septomaxilla dorsomedially, the prefrontal posterodorsally, the palatine ventromedially, and the ectopterygoid posteriorly. In some specimens the anteromedial process of the maxilla may contact the vomer in palatal view. Overall the maxilla is straight, slender, and dorsoventrally compressed except for the bluntly triangular ascending process positioned slightly anterior to the midpoint of its anteroposterior length. Although the anterior half of the bone may show a slight, gentle curvature medially, it lacks the strong curvature present in *Melanophidium wynaudense* ([17]:fig. 2) and *Platyplectrurus madurensis* ([55]:fig. 2.34). The anteriormost portion of the maxilla is edentulous.

#### *Uropeltis woodmasoni*

In lateral view, the ventral edge of the maxilla is slightly irregular, with dorsally directed emarginations marking tooth positions on the ventral surface. In articulated specimens of *U. woodmasoni*, the maxilla meets the premaxilla in a fairly straight, vertical suture (Fig. 2A). In disarticulation, the anterior tip of the maxilla is broadly Y-shaped, but the anterior surface is relatively flat for contact with the transverse process of the premaxilla (Fig. 3C). Posterior to that contact, the maxilla overlaps the septomaxilla laterally for the entire exposed length of the septomaxilla. The dorsolateral margin of the maxilla curves up and around the posterior corner of the septomaxilla to form the bluntly triangular ascending process of the maxilla, which abuts the anterolateral face of the prefrontal. Posteriorly, the lateral surface of the prefrontal is curved and thus forms an additional abutting or overlapping contact with the maxilla, creating an interlocking relationship between the elements. Posterior to the ascending process, the maxilla tapers to a point (Fig. 3A,B). The slender posterior portion of the maxilla underlaps and sits lateral to the anterior portion of the ectopterygoid. The contact between the two elements extends along the entire length of their tapered ends and is clearly visible in dorsal view (Fig. 5A). In lateral view, the anterior portion of the ectopterygoid is hidden from view.

Two foramina are visible in lateral view. The larger one is positioned at the anterior end of the maxilla, at the level of the posterior half of the anteromedial process; the smaller is ventral to the anterior half of the ascending process (Fig. 3A). The anterior tip of the maxilla is expanded and rounded in the area of the anterior foramen. In three specimens (TMM M-10003, -10008, -10010), a third foramen is visible between the two main foramina, and in two specimens (TMM M-10008, -10021) the anteriormost opening is positioned farther dorsally and is thus unbounded by the dorsal margin of the maxilla.

In dorsal view of the disarticulated element, a groove for the alveolar nerve [17] is visible extending anteriorly from the medially directed palatine process to (TMM M-10021) or just past (TMM M-10001) the anterior-most lateral foramen (Fig. 3C). The groove exits though the foramen in TMM M-10021, but in TMM M-10001 it continues anteriorly, and at the level of the anteromedial process the dorsal margin of the bone folds over medially to create a narrow partial roof over the groove, which opens completely again anteriorly. The posterior, large lateral foramen (and the middle one, when present) also communicates with the groove. At the base of the palatine process, the floor of the groove is pierced by a minute foramen leading into the body of the bone dorsal to the tooth row. In TMM M-10021 an additional foramen occurs on each side; on the right the foramen also lies at the base of the palatine process, but on the left it is positioned just ventral to the posterior lateral foramen.

A small, roughened articulation facet for the lateral foot process of the prefrontal occurs along the dorsal margin of the maxilla, just posterior to the ascending process. It is recognizable in the disarticulated element but is not well developed. In medial view, a short, distinct shelf occurs medial to the ascending process and dorsal to the palatine process and alveolar groove (Fig. 3B,C).

Ventrally, in all observed specimens of *U. woodmasoni*, the anterior extent of the tooth row begins between the anteriormost and posteriormost lateral foramina (Fig. 3B). Posteriorly, the tooth row continues past the level of the anterior contact with the ectopterygoid; posterior to the last tooth position, an edentulous smooth space about the length of 1–1.5 tooth sockets extends to the posterior tip. The anteromedial process of the maxilla extends medially to overlaps the septomaxilla and may contact the anterolateral process of the vomer. This contact between the maxilla and vomer was mentioned previously as being unique among uropeltids [17]. In most of our specimens of *U. woodmasoni* the two processes barely contact, and in others the two bones meet on only one side of the head, or not at all. It is likely that differential drying of soft tissue contributes to the variation observed among specimens for the presence or absence of that contact. Where the two processes do not meet, the palatal tubercle of the septomaxilla intervenes to separate them. About halfway along the length of the maxilla posteriorly, a second (palatine) process extends medially to underlap the palatine (Fig. 3C).

**Figure 9:**
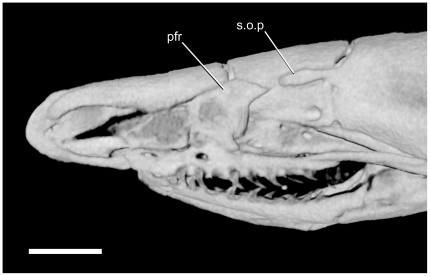
Lack of contact between prefrontal and supraorbital process of parietal of *Uropeltis woodmasoni* (TMM M-10006). Reconstruction from CT scan, left lateral view, anterior to the left. pfr = prefrontal; s.o.p = supraorbital process of the parietal.

The maxillary teeth are homodont, with a sharply pointed and backward projecting tip (i.e., recurved; Fig. 9). Most (n = 9) of our 11 specimens have eight tooth positions on each maxilla (Table 1). There are only six on each side of TMM M-10002; TMM M-10005 has eight positions on the left and six on the right. The largest tooth usually occurs ventral to the ascending process of the maxilla.

#### *Uropeltis rubromaculata*

The shape and proportions of the maxilla in lateral view differ in *U. rubromaculata*. More than half the length of the bone tapers posteriorly. The portion of the bone anterior to the ascending process is dorsoventrally much deeper than in *U. woodmasoni*, giving the (false) impression that the anterior portion has been anteroposteriorly compressed (Fig. 2B). The ventral margin of the maxilla in lateral view is more strongly irregular than in *U. woodmasoni*. At the contact with the premaxilla, the lateral surface extends below the ventral margin of the premaxilla.

A shallow, crescentic emargination occupies the dorsal edge of the anterior tip of the maxilla, so that the edge in lateral view slopes upward posteriorly to the level of the contact with the septomaxilla. A sharply angled inflection occurs at that point, and the margin continues to curve more gently posterodorsally until it forms the anterior margin of the ascending process (Fig. 2B). The peak of the ascending process is at the junction of the maxilla, septomaxilla, and prefrontal (there is no significant exposure of the prefrontal anterior to the ascending process). The base of the ascending process coincides with the farthest ventral extent of the lateral foot process of the prefrontal. There are three maxillary foramina. The anteriormost and largest foramen is just posterior to the beginning of the contact with the septomaxilla. A tiny middle opening is positioned along the curved slope, halfway between the contacts with the septomaxilla and prefrontal. The posterior-most foramen is located directly below the ascending process. A large soft-tissue-filled gap separates the lateral wall of the septomaxilla from the portion of the maxilla anterior to the ascending process.

As in *U. woodmasoni*, the maxilla and ectopterygoid of *U. rubromaculata* have a long mediolateral contact, with the maxilla lateral to the ectopterygoid. However, in *U. rubromaculata* the posterior rim of the posteriormost tooth position coincides with the beginning of the contact with the ectopterygoid. In ventral view, the palatine process is broader, larger, and more rounded medially than in *U. woodmasoni* (Fig. 4B). The maxilla and vomer do not contact in palatal view. The teeth are much larger and fewer in number than in any other taxon examined (six positions on the left, five on the right). Our tooth count is consistent with a previous report of five maxillary teeth in *U. rubromaculata* [15].

#### *Uropeltis melanogaster*

The groove for the alveolar nerve could be observed in both disarticulated specimens. On the left side of TMM M-10032 the groove pinches slightly between the anteromedial and palatine processes, but is at no point fully roofed. On the right, roofing is complete for a short distance just posterior to the anterior lateral foramen, as in *U. woodmasoni*. On both sides, the anterior end of the groove is closed by a low wall rather than being open anteriorly. In TMM M-10045, however, on both the left and right sides, the alveolar groove is roofed only partially, and a shallow channel over the wall closes the anterior end of the groove (Fig. 3F). Where the alveolar groove is partially roofed in TMM M-10045, a portion of the medial side of the roofing wall is medially inflected, so that in dorsal view there is an additional medial process between the anteromedial and palatine processes (two occur on the left side of TMM M-10032). The medial inflection is weak, and in no case does the additional process extend as far medially as the two major medial projections. The posterior end of the groove forms a deep and wide pocket just lateral to the palatine process and curves medially to continue along the dorsal surface of that process. In both specimens, a tiny foramen pierces the floor of the pocket and passes into the bone just dorsal to the tooth row. A pronounced tubercle occurs on the posterolateral edge of palatine process in both specimens (it is more strongly developed in TMM M-10045); this tubercle is positioned between the groove on the dorsal surface of the palatine process and the roughened area for the articulation of the prefrontal. In both specimens the anteriormost lateral foramen is located slightly more posteriorly than in *U. woodmasoni*, just posterior to the level of the anteromedial process. In TMM M-10032 the articulation with the transverse process of the premaxilla would have been vertical and straight, but a small, anteriorly projecting process on the dorsal surface of the anterior tip of the maxilla probably overlapped the premaxilla slightly. The small anterior process is not as well developed in TMM M-10045 (the larger specimen). The posterior end of the specimen tapers gradually, but then is stepped to form a squared terminal tip (Fig. 3D). There are seven tooth positions on each side on both specimens. The tooth row extends posterior to the level of the anteriormost contact with the ectopterygoid in TMM M-10045 (Fig. 3E); in TMM M-10032, the tooth row ends just posterior to what would have been the anteriormost contact with the ectopterygoid, based on the position of the articulation facet.

#### *Rhinophis blythii*

The maxilla resembles that of *U. woodmasoni* in lateral view, with a horizontal, unsloped dorsal surface anterior to the ascending process (Fig. 8). The process is narrower than in *U. woodmasoni* and more rounded at its apex. Additionally, the anteriormost foramen is more posteriorly located and the anterior tip of the maxilla is slightly taller, extending ventrally just past the ventral margin of the premaxilla in lateral view. Seven tooth positions occupy each side, and the posteriormost tooth is positioned at the level of the anterior-most contact with the ectopterygoid.

#### *Rhinophis drummondhayi*

The maxilla more closely resembles that of *U. woodmasoni* than *R. blythii* in lateral view, because the anterior tip does not extend ventrally past the premaxilla at the suture between the two elements, but the maxilla does deepen just posterior to that suture (Fig. 8B). The posteriormost lateral foramen is entirely anterior to the ascending process. In ventral view, the palatine process is small and does not extend far medially (Fig. 7B). Seven tooth positions occur on each side, and the posteriormost tooth is positioned at the level of the anterior-most contact with the ectopterygoid.

#### *Rhinophis philippinus*

The anterior lateral foramen of the maxilla is positioned more posteriorly than in *U. woodmasoni* (as it is in *R. blythii*). The posterior-most foramen is positioned ventral to the anterior half of the ascending process. In lateral view, the contact with the premaxilla is vertical ventrally, but dorsally a distinct process of the maxilla extends anteriorly and rests on the dorsal margin of the transverse process of the premaxilla (Fig. 8C). In ventral view, the anteromedial process is reduced, but it does contact a well-developed anterolateral process of the vomer (Fig. 7C). In dorsal view, the alveolar nerve groove is narrow and ends at the anteriormost lateral foramen, where the groove becomes pinched. At its posterior end, a tiny foramen penetrates the body of the bone, but a distinct pocket is lacking. There are five tooth positions on each side in both specimens. The posterior-most tooth is anterior to the anteriormost contact with the ectopterygoid.

#### *Rhinophis homolepis*

As in the other *Rhinophis*, the anterior lateral foramen of the maxilla is positioned more posteriorly than in *U. woodmasoni*. The lateral maxillary foramina are proportionately larger than any other taxon surveyed. The posterior-most foramen is located ventral and slightly posterior to the midpoint of the ascending process (Fig. 8D). Ventrally, the anteromedial process is reduced, and the palatine process has a triangular, posteriorly directed point (Fig. 7D). The vomer and maxilla contact in palatal view. There are seven tooth positions on each side, and the posteriormost tooth is positioned just anterior to the anteriormost contact with the ectopterygoid.

#### *Brachyophidium rhodogaster*

*Brachyophidium rhodogaster* expresses more individual variation in the maxilla than within species referred to either *Uropeltis* or *Rhinophis*. In lateral view, the element is proportionately longer and narrower than in the other genera (Fig. 2C). The anterior-most tip has a pointed process that extends anteriorly to overlap the transverse process of the premaxilla (Fig. 3G). Between the extended process of the maxilla and the transverse process of the premaxilla, a gap is filled with soft tissue. The dorsal margin of the maxilla slopes upward posteriorly from its anterior edge to meet the septomaxilla. The slope levels out at the level of the anterior-most contact of the maxilla with the septomaxilla. In TMM M-10019 the anterodorsal surface of the maxilla is strongly notched on the right side only. Between that notch and the ascending process, the dorsal margin is shallowly concave. TMM M-10015 has a similar shape, though slightly reduced. In all other specimens the dorsolateral margin remains level until the ascending process (Fig. 3G), which is broad, rounded, and short, except in two specimens in which the process is tall and narrow on one side (TMM M-10020, left; TMM M-10015, right). The apex of the process occurs just posterior to the junction with the septomaxilla and the prefrontal, as in most of the specimens referred to *Uropeltis* and *Rhinophis*. As in those specimens, the lateral foot process of the prefrontal overlaps the ascending process posteriorly.

Individuals can have two (TMM M-10026) or three (TMM M-10011, -10014, -10019, -10020, -10022–10024) lateral maxillary foramina on each side, or may have differing numbers between the right and left (TMM M-10013, three on the right but the central and posterior ones are confluent; TMM M-10015, -10017, -10018 three on right; TMM M-10016, three on left). The anterior-most foramen is usually located ventral to the point where the dorsal margin flattens, anterior to the anteromedial process. The posteriormost foramen can be below the anterior (TMM M-10011, -10013–10018, -10020, -10023, -10026) or posterior half (TMM M-10019) of the ascending process, or centered directly below it (TMM M-10022, -10024). When a third, middle foramen is present, whether it is positioned closer to either the anterior or posterior foramen varies individually. The contact with the ectopterygoid is not as long as in the other sampled taxa, and begins immediately posterior to the last tooth socket (Fig. 3H). In ventral view, the teeth terminate anteriorly just posterior to the anteromedial process, which is reduced or absent (e.g., TMM M-10024) in some specimens. The palatine process, however, is always large, broad, and roughly triangular (Fig. 4C). All specimens examined have nine maxillary teeth, except for TMM M-10025, which possesses only eight and may be misidentified (Table 1).

The posterior part of the transverse process of the premaxilla slots into the space between the anteromedial process and the anterior tip of the maxilla (Fig. 3I), forming a clasping articulation between the two elements. In palatal view, the entire anterior surface of the anteromedial process forms a firm articulation with the transverse process of the premaxilla (unlike in *U. woodmasoni*, in which only a small lateral portion of the anterior surface contacts the premaxilla).

In lateral view, the anteromedial process is visible as a ventral projection extending beyond the ventral margin of the premaxilla (Figs. 3G,4C). In the majority of specimens, the anteromedial process of the maxilla does not meet the anterolateral process of the vomer in palatal view. In TMM M-10011, however, the two processes meet on the left, but not on the right, and in TMM M-10014 there is a contact on the left, but damage on the right precludes assessment.

When viewed in disarticulated specimens, the anteromedial process is highly variable anteriorly. Just ventral to the anteriormost foramen, the anterior face of the process in TMM M-10018 has a partially-roofed notch that receives the transverse process of the premaxilla. TMM M-10016 has a similar notch, but lacks the dorsal shelf. TMM M-10013, TMM M-10024, and TMM M-10026, have a planar anterior surface, but the process is weakly developed in the latter two.

In dorsal view, most disarticulated specimens have a well-developed alveolar groove as exhibited in the other taxa, although the groove is not usually roofed in *B. rhodogaster*. In TMM M-10018 the alveolar groove is shallow and reduced along the inside of the lateral wall. Nearly all specimens possess a roughened surface or shallow groove posterior to the ascending process that marks the prefrontal articulation. However, individuals express various degrees of roughening, and some (e.g., TMM M-1016, -10023) also have a thin shelf of bone projecting medially from the base of the ascending process, as in *U. woodmasoni* (Fig. 3H,I). TMM M-10022 has the most complicated articulation area, in which the prefrontal articulation surface is expanded to form a broad hook-shaped shelf on the left side that projects medially from the lateral wall (on the right, the hook is not well developed, but a significant medial projection is present). In that specimen the alveolar nerve groove begins directly ventral to the shelf and may coincide with the internal opening for the posterior-most lateral foramen. In TMM M-10022 and TMM M-10023, a distinct pocket is formed ventral to the shelf; a small foramen penetrates ventrally within the pocket in TMM M-10023 and on the right side of TMM M-10022.

### Septomaxilla

The septomaxilla is a complex bone that can be conceptualized as having three main components. The first is a lateral ascending wall that curves dorsomedially; the second is a more-or-less horizontal medial sheet of bone that forms the dorsal portion of the broad, vomeronasal capsule; the third is a short, vertically oriented nasal buttress ( = medial flange of [32]) that ascends from the base of the medial edge of the bone. The septomaxilla contacts the maxilla ventrolaterally, prefrontal posteriorly, nasal dorsally and medially, vomer ventrally, the contralateral septomaxilla medially, and premaxilla anteriorly. Additionally, the septomaxilla forms the lateral margin of the vomeronasal opening of the vomer. In lateral view, the septomaxilla is overlapped by the maxilla, prefrontal, and nasal. The degree of overlap varies within and among taxa, sometimes yielding a triangular profile and sometimes a rectangular profile in lateral view.

#### *Uropeltis woodmasoni*

The septomaxilla overlies the maxilla at a straight, horizontal contact that terminates anteriorly just posterior to the suture between the maxilla and premaxilla. The posterior margin of the septomaxilla does not contact the ascending process of the maxilla because a thin section of the prefrontal and associated soft tissue intervene. Dorsally, the septomaxilla curves medially to underlie a short portion of the lateral margin of the nasal anteriorly and a section of the prefrontal posteriorly. The anterior extent of its lateral exposure is usually to the level of the suture between the premaxilla and maxilla. In some specimens, the septomaxilla is in direct contact with the maxilla immediately above that suture, and its anterior extent essentially forms a vertical wall (e.g., TMM M-10007, right side of TMM M-10009 and TMM M-10010). In other specimens, the ventral part of the anterolateral exposure is emarginated, isolating a short (e.g., TMM M-10004), or relatively long, finger-like process reaching to the level of the premaxilla-maxilla suture, or just beyond it (e.g., TMM M-10008).

**Figure 10:**
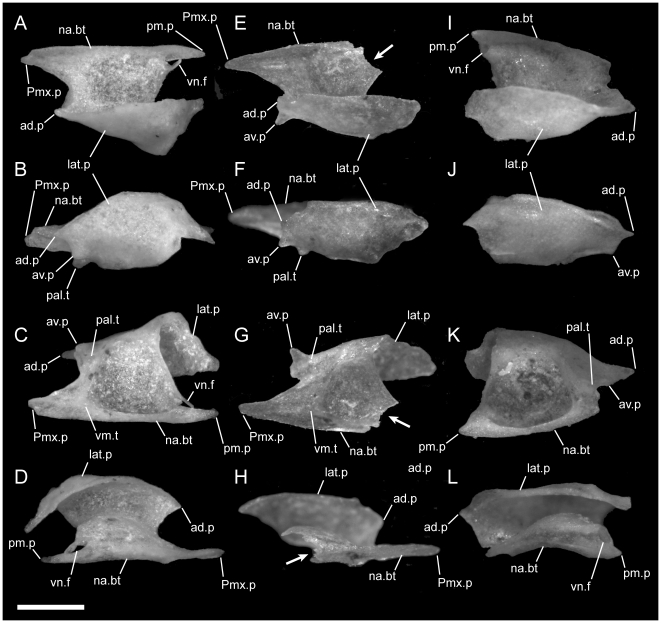
Disarticulated septomaxillae. Anterior is to the right in D,H–K; anterior is to the left in all others; scale bar = 0.5 mm. A–D from the left side of *U. woodmasoni* (TMM M-10001); E–H from the left side of *U. melanogaster* (TMM M-10045); and I–L from the right side of *B. rhodogaster* (TMM M-10022). A,E,I in lateral view; B,F,J in lateral view; C,G,K in ventral view; and D,H,L in medial view. Arrow denotes broken posteromedial portion of bone in *U. melanogaster* specimen. ad.p = anterodorsal process of lateral process; av.p = anteroventral process of lateral process; lat.p = lateral process (lateral wall); na.bt = nasal buttress; pal.t = palatal tubercle; pm.p = posteromedial process; Pmx.p = premaxillary process of septomaxilla; vm.t = ventromedial tubercle; vn.f = vomeronasal foramen.

Internally, the septomaxilla broadly overlies the vomer. It is excavated ventrally into a dorsally convex cupola that forms the roof of the vomeronasal chamber. In ventral view, the cupola is subcircular in shape and is circumscribed by crests anteriorly, laterally, and medially, but is open posteriorly (Fig. 10C). Anterior to the cupola, a distinct, triangular premaxillary process on the medial side fits into the space between the nasal and vomerine processes of the premaxilla (Figs. 2A; 10A,C). Two small tubercles occur on the ventral surface in this region. One is positioned at the base of the premaxillary process, near the junction of the medial and anterior crests surrounding the cupola. A second, the palatal tubercle, sits at the anteroventral corner of the lateral edge of the bone and often is visible in palatal view of the articulated skull, as a small exposure at the junction of the maxilla, premaxilla, and vomer (Figs. 3A,10B).

The medial margin of the bone (just medial to the cupola) is swept up to form the nearly vertical nasal buttress, which extends anteriorly to form the medial margin of the premaxillary process (Fig. 10A,D). Dorsally, the buttress contacts the medial process of the nasal. Posteriorly the nasal buttress extends into a long, thin, pointed posteromedial process that closely approaches, and in some specimens may contact (e.g. TMM M-10010), the anteroventral portion of the lateral frontal flange of the frontal. On the lateral side of the base of the posteromedial process, a round foramen for the vomeronasal nerve [32] is present (Fig. 10A). The foramen is closed laterally by a thin splinter of bone extending from the posterior margin of the cupola in TMM M-10001 and on the right side of TMM M-10021; in those specimens the thin splinter is not fused with the medial wall of the process. The foramen is fully closed by a relatively robust strut of bone on the left side of TMM M-10021, and a small accessory foramen pierces that strut immediately posterior to the vomeronasal nerve foramen.

**Figure 11:**
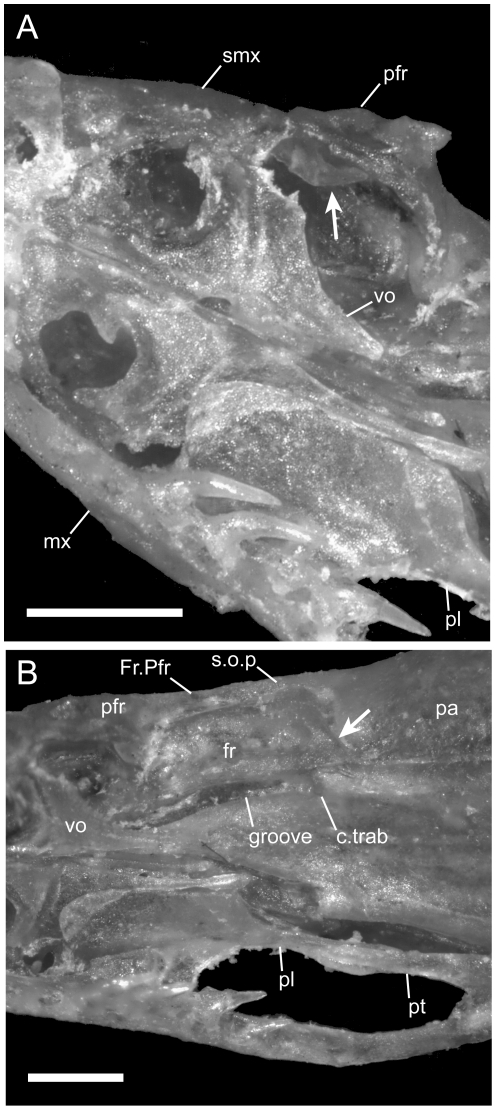
Magnified view of the palate of *U. woodmasoni* (TMM M-10010) with disarticulated left maxilla and palatine in ventral view. Scale bars = 0.5 mm. (A) Partial disarticulation reveals contact of the septomaxilla (arrow) with the medial surface of the prefrontal. Note broken palatine process of vomer on left side of animal. Anterior is toward the upper left corner. (B) The groove for the cartilaginous portion of the crista trabecularis is formed between the sphenoid region of the otooccipital complex and the frontal. Arrow points to the ventrolateral part of the frontal-parietal suture. Anterior is to the left. c.trab = ossified base of crista trabecularis; fr = frontal; Fr.Pfr = frontal process of prefrontal; groove = groove for the cartilaginous portion of the crista trabecularis; mx = maxilla; pa = parietal; pfr = prefrontal; pl = palatine; pt = pterygoid; smx = septomaxilla; s.o.p = supraorbital process of the parietal; vo = vomer.

In disarticulated septomaxillae, the lateral ascending wall curves medially to form a narrow dorsal roof over the nasal passage (Fig. 10A,D). At its anterior end, the lateral wall forms two processes, one dorsal and one ventral (Fig. 10B,C). An anterodorsal pointed process is visible in lateral view even in articulated skulls, where it is located ventral to the nasals and anterior to the contact with them. The anteroventral process is squared and participates in the articulation with the maxilla. This process is dorsal to the palatal tubercle and separated from it by a lateral groove. The posterior end of the lateral ascending wall is inflected posteroventrally and terminates in a broad triangular prefrontal process that underlies the prefrontal (Fig. 11A). The base of the prefrontal process is notched ventrally.

#### *Uropeltis rubromaculata*

The lateral exposure of the septomaxilla in our specimen (TMM M-10028) is proportionately rounder and shorter than in *U. woodmasoni*, extending a shorter distance anteriorly (Fig. 2B). Internally, as in *U. woodmasoni*, the premaxillary process of the septomaxilla contacts the premaxilla. This is a large specimen (Table 1), and when viewed through the nares, the septomaxilla can be observed contacting the premaxilla along the posteromedial surface of the transverse process from the midline to the point where the two bones meet the maxilla. The anterodorsal process of the lateral ascending wall is less well-developed than in *U. woodmasoni*, but in contrast, the palatal tubercle appears much larger and is more visible in ventral view, jutting between the anterolateral edge of the vomer, the maxilla, and the premaxilla (Fig. 4B). Ventrally, the crest ringing the median vomeronasal fenestra and flanking the vomer along its lateral margin is also more pronounced than in *U. woodmasoni*.

#### *Uropeltis melanogaster*

The lateral ascending wall is not as strongly inflected medially as it is in *U. woodmasoni* and so does not provide as much of a roof over the nasal passage (Fig. 10E). In our smallest specimen (TMM M-10032) the anterodorsal and anteroventral processes of the lateral wall are reduced and rounded, the palatal tubercle and the crests surrounding the cupola are extremely reduced, and the ventral tubercle at the base of the premaxillary process is absent. Those structures are well developed in the larger specimen (TMM M-10045, Fig. 10E–G). The anteroventral process in TMM M-10045 is narrower and more sharply pronounced, unlike the more rounded condition in *U. woodmasoni*. The posterior portion of TMM M-10045 is broken on both sides. In TMM M-10032, the posterior portion of the nasal buttress is shorter than in *U. woodmasoni*, but it terminates in a similar sharply pointed posteromedial process. The lateral side of the base of that process is notched, marking the passage of the vomeronasal nerve. Similarly, the premaxillary process is narrower and less robust than in *U. woodmasoni* and *U. rubromaculata*. The posterior margin of the cupola slopes laterally, as opposed to the straight margin in *U. woodmasoni*.

#### *Rhinophis blythii*

The shape of the septomaxilla is similar to that of *U. woodmasoni* in lateral view, but is more rounded overall. In lateral exposure, its anterior margin curves so that the ventral portion extends farther anteriorly than the dorsal (Fig. 8A). The septomaxilla reaches its anteriormost extent at the level of the premaxilla-maxilla suture and contacts both bones at that suture. There is no palatal tubercle visible between the junction of the vomer, premaxilla, and maxilla. The vomer and maxilla meet in palatal view because of complete underlap of the septomaxilla by the vomers and a robust anteromedial process of the maxilla (Fig. 7A).

#### *Rhinophis drummondhayi*

The lateral exposure of the septomaxilla is narrower and longer than in *R. blythii* (Fig. 8B). Anteriorly, the bone extends anterior to the premaxilla-maxilla suture, and its lateral margin is curved as in *R. blythii*. As in *U. melanogaster*, the lateral ascending wall is not inflected medially to form a dorsal roof over the nasal passage. Internally, when viewed through the nares, the vomer is not visible below the septomaxilla, although as in *R. blythii*, the premaxillary process of the septomaxilla does not completely fill the gap between the nasal process and floor of the premaxilla. In ventral view a small part of the palatal tubercle is visible between the premaxilla, vomer, and maxilla.

#### *Rhinophis philippinus*

In the articulated skull, the septomaxilla is long and narrow in its lateral exposure (Fig. 8C). Anteriorly, the septomaxilla extends to the posterior portion of the suture between the maxilla and premaxilla (a finger-like process of the maxilla in this species extends over the premaxilla). The dorsal and ventral anterior processes of the anterolateral surface are reduced markedly. A palatal tubercle is barely visible in palatal view on the left side, sitting anterior to the contact of the maxilla and the vomer. On the right, the maxilla is removed, and a stout tubercle is visible; it is not clear whether it would have been completely obscured if the maxilla was articulated. The disarticulated septomaxilla of TMM M-10038 also shows a small tubercle in that position. The premaxillary process is longer and comes to a sharper point distally than it does in *U. woodmasoni*. This specimen also reveals that *R. philippinus* has a short posteromedial process. Just lateral to its base, a small posteromedial foramen for the vomeronasal nerve pierces the posterior portion of the cupola. The lateral surface is a vertical sheet of bone, with almost no medial tilt dorsally.

#### *Rhinophis homolepis*

In *R. homolepis*, the lateral exposure of the septomaxilla is approximately rectangular in lateral view and lacks the curved anterior margin seen in other *Rhinophis* species (Fig. 8D). It extends anteriorly beyond the suture between the maxilla and premaxilla, and contacts both bones ventrally. Palatal tubercles are visible in ventral view.

#### *Brachyophidium rhodogaster*

In lateral view of the articulated skulls of *B. rhodogaster*, the septomaxilla extends farther anteriorly than the anteriormost extent of the elongated premaxilla-maxilla suture (Fig. 2C). The exposed surface of the lateral wall is long and tapers sharply anteriorly, beginning about half the distance along its length. As in the other taxa, the septomaxilla is overlapped by the maxilla, prefrontal, and nasals. Similar to *U. woodmasoni*, the lateral wall folds over medially to form a dorsal roof that covers less than half of the vomeronasal cupola (Fig. 10I). Posteriorly, when disarticulated, the lateral wall ends in a tab-like process that underlies the prefrontal. Unlike in *U. woodmasoni* and *U. melanogaster*, in dorsal view, the medial edge of the nasal buttress curves down medially rather than being upswept.

There is no premaxillary process on the medial side of the bone. The anteroventral portion of the lateral wall is inflected medially and forms a bony lamina that, in anterior view, is exposed as an angled sheet within the external naris. That lamina extends medially, and its ventral side forms the dorsal part of a shallow tube completed ventrally by the premaxilla, maxilla, and vomer. In the disarticulated septomaxilla, a low, short crest and reduced palatal tubercle are anterior to the cupola and ventral to the point where the medial inflection starts (Fig. 10J,K). The tubercle is more strongly developed in larger specimens (e.g., TMM M-10023), but usually is not visible in palatal view of articulated skulls. In two specimens (TMM M-10019, -10020) a thin sliver of septomaxilla is visible at the junction of the premaxilla, maxilla, and vomer, but as in the others there is no true ventral projection. In all specimens the maxilla covers the anterior extent of the septomaxilla in ventral view.

The posteromedial corner of the nasal buttress completely encloses the vomeronasal posteromedial foramen, forming a short tube (visible only in the disarticulated element, Fig. 10I,L). The posteromedial process associated with this foramen is short and triangular in *B. rhodogaster*. The open, posterior margin of the cupola for the vomeronasal organ is rounded and upswept, as opposed to the condition in *U. woodmasoni and U. melanogaster*.

### Nasal

The nasal is the anterior-most skull roofing bone and forms the dorsal margin of the external naris. The nasal contacts the premaxilla anteromedially, septomaxilla laterally, prefrontal posterolaterally, frontal posteriorly, and the contralateral nasal medially, and has two distinct and smooth surfaces. The dorsal lamina is convex dorsally and curves laterally to form a continuous surface with a variably extensive lateral lamina. A medial process is well developed in all taxa but not visible in articulated skulls. The process is most extensive posteriorly, but its height is progressively reduced anteriorly, and it is absent at the anterior end of the bone.

#### *Uropeltis woodmasoni*

**Figure 12:**
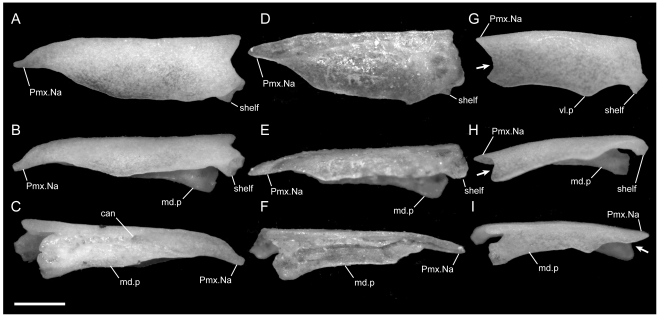
Disarticulated nasals. Anterior is to the left unless noted; scale bar = 0.5 mm. All elements from the left side of the skull. A–C from *U. woodmasoni* (TMM M-10001); D–F from *U. melanogaster* (TMM M-10045); and G–I from *B. rhodogaster* (TMM M-10022). A,D,G in dorsal view; B,E,H in lateral view; and C,F,I in medial view (anterior is to the right). can = anterior opening of the canal within the medial process of the nasal; md.p = medial process; Pmx.Na = premaxillary process of nasal; shelf = shelf that is continuous with pre-orbital ridge of frontal; vl.p = ventrolateral process at triple junction.

In *U. woodmasoni*, the nasals contact each other medially along a straight suture from their posterior contact with the frontals until approximately three-quarters of their length anteriorly (Fig. 5A). At the point of overlap with the premaxilla, the premaxillary processes of the nasals diverge laterally. The nasal process of the premaxilla is visible dorsally as a wedge located in the fork between the two nasals. The nasals overlap the premaxilla up to the point where the rostral process of the premaxilla expands laterally. In both dorsal and lateral views, the nasal tapers anteriorly (Figs. 2A; 5A; 12A,B). In lateral view, a broad, crescentic ventral emargination forms the dorsal border of the external naris. In most specimens, the emargination begins at the anterior point of contact with the septomaxilla and increases in a gradual curve anteriorly. In TMM M-10003, TMM M-10005, and TMM M-10010, the emargination begins anterior to that contact. The anterolateral extent forms a rounded surface with a slight ventral inclination (Fig. 2A); dorsally the anterior end of the nasal appears as an elongated, pointed premaxillary process.

The ventral edge of the medial process of the nasal contacts the nasal buttress of the septomaxilla, in an articulation that cannot be seen in the articulated skull. In lateral view, the two elements form a straight, posterodorsally oriented suture. Ventrally, the nasal overlaps a small portion of the lateral septomaxilla internally. Contact with the prefrontal occurs posteriorly along a curved suture, following the shape of the anterodorsal portion of the prefrontal. The suture with the frontal varies individually and can be relatively straight in dorsal view, or it can form a curved (e.g., TMM M-10009) or sharply angled (e.g., TMM M-10003) notch in the posterior nasal, forming distinct posterolateral and sometimes posteromedial processes on the bone. In TMM M-10006, the right nasal has a slightly larger posterolateral process. The nasal-frontal suture is accompanied by an extensive amount of soft tissue (appearing as bright white areas in Figure 5A); some specimens show left-right asymmetry in the relative anteroposterior position of the nasals (e.g.,TMM M-10009), but that condition appears to be a result of differential drying of specimens during preparation of dry skeletal material.

By examining isolated nasals and data from a CT scan of TMM M-10006, it is clear that the medial process is a vertical wall of bone that abuts the same process of the contralateral nasal for much of their length (anteriorly, the nasal process of the premaxilla separates them). The medial process forms a gentle ventrolateral curve along most of its length, but posteriorly it is more strongly inflected laterally (Fig. 12C). Posteriorly, the medial face consists of alternating rugosities and concavities that form an interlocking articulation between the nasals (fig. 2.26A,B [55]). In our larger disarticulated specimen (TMM M-10021), the rugosities are more pronounced than in the smaller specimen (TMM M-10001), indicating that their degree of development may be subject to ontogenetic variation.

About midway along the anteroposterior length of the medial process, the anterior opening of an anteroposteriorly oriented canal is marked by a foramen on the dorsal part of this wall. It is most visible in anterior or anteromedial view, but is observable in direct medial view (Fig. 12C). Anterior to that opening, the bone is grooved with a shallow channel. Additional minute foramina pierce the medial process posterior to that opening and enter into the canal; a single foramen occurs in TMM M-10021, and two occur in TMM M-10001. The posterior end of the canal opens on the lateral side of the medial process, just posterior and ventral to the anterior end of a pronounced crest that extends from the ventral surface of the lateral side of the dorsal lamina, curving medially and somewhat ventrally as it extends anteriorly to terminate near the junction of the dorsal lamina and the medial process. When we manually articulate isolated elements, it appears that the canal would be continuous with the shallow groove on the lateral surface of the nasal process of the premaxilla. Ventrally, the nasal is concave and smooth anterior to the crest. Posterior to the crest, a relatively large triangular area accepts the olfactory process of the frontal [55] (="transverse frontal ridge” of [17], ), which inserts into the posterior part of the nasal to participate in a strong, interlocking articulation (Figs. 5A, 12A). Two rounded tubercles are visible in the posterior view of the nasal. One is positioned dorsolaterally at the medial edge of the prefrontal shelf (see below) and another occurs at the lateral edge of a strong lateral inflection of the posteriormost portion of the medial process. The two tubercles also participate in the interlocking articulation with the frontal. That complex arrangement suggests that little kinetic movement is possible at this joint. On the right side only of TMM M-10001 and TMM M-10021, a third, small tubercle projects medially from the wall of the medial process, dorsal to the one positioned at the lateral inflection, and may facilitate articulation with the contralateral nasal. In dorsal view of the disarticulated specimen, a small, triangular process at the posterolateral corner forms a low shelf ventral to the dorsal surface of the nasal (Fig. 12A,B). It forms the anterior part of a continuous shelf that underlies the prefrontal; the posterior part of that shelf is formed by a corresponding structure on the lateral side of the frontal (the ‘preorbital ridge’ of [17], [60]).

#### *Uropeltis rubromaculata*

The nasals have a broader dorsal surface than in *U. woodmasoni* (Fig. 5B). Tapering of the nasals in dorsal view begins farther anteriorly and in lateral view occurs at a much shallower angle, forming a triangular point anteriorly rather than the curved edge seen in *U. woodmasoni* (Fig. 2B). This is associated with the more blunt appearance of the tip of the snout in *U. rubromaculata*. In addition, in lateral view the ventral surface is less emarginated than in *U. woodmasoni*. The contact with the frontal is subtly angled posterolaterally and has fine-scale undulations along the suture.

#### *Uropeltis melanogaster*

Tapering of the nasals begins even farther anteriorly than in *U. rubromaculata*. Tapering is more abrupt than in *U. woodmasoni*, and leaves only a narrow, rounded finger of bone projecting anteriorly in dorsal view (Fig. 12D). In lateral view, the dorsal emargination is abrupt and squared (Fig. 12E). The medial process is similar to that in *U. woodmasoni*. In one specimen (TMM M-10032) a single medial rugosity occurs posteriorly; the second specimen (TMM M-10045) possesses two. Neither specimen shows the medial canal seen in *U. woodmasoni*. Instead, a pronounced channel traverses the length of the dorsal portion of the medial process (Fig. 12F). In TMM M-10032 the channel bifurcates anteriorly, and on the right side of TMM M-10045 one of the medial rugosities expands to form a short canal near the anterior end of the channel. At the posterior end of the channel, a foramen pierces the medial process and opens laterally at the posteroventral edge of a low crest (i.e., in a position similar to the posterior opening of the canal in *U. woodmasoni*). The crest in *U. melanogaster* is less well developed in TMM M-10032, but quite robust in TMM M-10045. The prefrontal shelf is less well developed than in *U. woodmasoni*, but is stronger on TMM M-10045 than on TMM M-10032 (Fig. 12D,E). The two posterior tubercles in *U. woodmasoni* that serve as accessory articulations for the frontal are retained in *U. melanogaster*. The medial tubercle on the wall of the medial process is present, but reduced, on only the left side of TMM M-10045 and is absent on the right side of TMM M-10045 and on both sides of TMM M-10032. On both nasals of TMM M-10045 and the left side of TMM M-10032, the medial wall of the laterally inflected posterior portion of the medial process is deeply incised with a groove that continues a short distance anterodorsally on the medial process.

#### *Rhinophis blythii*

In dorsal view, the nasals appear to be slightly broader than in *U. woodmasoni* and taper anteriorly only at their tips (Fig. 6A). The nasals do not extend as far anteriorly as they do in *U. woodmasoni*, terminating well posterior to the expansion of the rostral process of the premaxilla. The lateral suture with the septomaxilla is more horizontal than in articulated *Uropeltis* specimens. The suture with the frontal is rounded along its central portion, with distinct lateral processes posteriorly, and small medial processes directed posteriorly between the frontals. No disarticulated material is available.

#### *Rhinophis drummondhayi*

The nasals most closely resemble those of *U. woodmasoni* in proportions and shape. The lateral suture with the septomaxilla is more horizontal. The suture with the frontal is straight but angled obliquely (Fig. 6B), because the lateral edge of the nasal extends farther posteriorly than does the medial edge.

#### *Rhinophis philippinus*

The anterior extent of the nasals in dorsal view is similar to that in *R. blythii*, but the lateral edges appear more rounded in dorsal view. The lateral suture with the septomaxilla is again more horizontal than in *Uropeltis*. The nasal-frontal contact appears similar to that of *R. drummondhayi* but is at a shallower angle (Fig. 6C). The suture is irregular and has fine undulations. In one specimen (TMM M-10037) the anterior tapering of the nasals in lateral view is less abrupt, and the emargination is more smoothly curved, similar to *U. woodmasoni*, whereas it is abrupt in the other (TMM M-10038) creating a highly angled inflection at the origin of the emargination in lateral view. In dorsal view, the nasal of TMM M-10038 also is slightly notched along the margin shared with the prefrontal. The partially disarticulated specimen (TMM M-10038) shows a well-developed prefrontal shelf, a groove (but not a canal) along the dorsal portion of the medial process, and a deeply incised groove on the medial surface of the lateral inflection at the posterior end of the medial process (similar to that in *U. melanogaster*). The internal crest at the posterior end of the bone is poorly developed (as in *U. melanogaster*, TMM M-10032). Small rugosities occur posteriorly on the medial surface of the medial process, and the two posterior tubercles for accessory articulation with the frontal are present.

#### *Rhinophis homolepis*

The lateral contact of the nasals with the septomaxilla is nearly horizontal and ends anteriorly at a point posterior to the initiation of the dorsal emargination (Fig. 8D). That emargination thus appears to be abrupt. In dorsal view, the nasals terminate at a point apparently even farther posterior than in *R. blythii* and *R. philippinus*. The suture with the frontals is at a shallow angle.

#### *Brachyophidium rhodogaster*

The nasals are more rectangular than in species referred to either *Uropeltis* or *Rhinophis*. Tapering occurs only at the anteromedial tip and is visible only in dorsal view (Figs. 5C,12G). The overall appearance is of a rectangular bone with an anterolateral notch. The nasal process of the premaxilla is exposed in the narrow space between the anterior ends of the nasals. In lateral view, the nasal shares an elongated contact with the septomaxilla; no dorsal emargination is present, but the posterior margin of the external naris excavates a shallow notch in the anterior surface of the nasal (Figs. 2C,12H).

At the triple junction with the prefrontals and septomaxillae, the disarticulated nasal has a weakly developed triangular process oriented laterally and slightly ventrally (Fig. 12G,H). In the articulated skull, that process appears to be strongly developed, because of the degree of overlap between the three elements. Posteriorly, the suture with the frontals is smooth and slightly curved, with only a slight lateral process developed on the nasal.

In most of the disarticulated specimens, the medial process is relatively shallow and almost uniform in height along most of its length (Fig. 12I). The process is deeper posteriorly, where it is notched just ventral to the junction with the dorsal lamina; the notch accommodates a small lateral projection on the olfactory process of the frontal. The medial process does not taper anteriorly in most specimens, but ends in a squared-off or slanted surface approximately perpendicular to the dorsal lamina (Fig. 12I). In TMM M-10026, the depth shallows anteriorly, with two step-like reductions in height. The medial process in *B. rhodogaster* is nearly vertical, lacking a lateral inflection for most of its extent. A slight lateral inflection occurs at the posterior end, where a small tubercle is developed as an accessory articulation point with the frontal. The other tubercles seen in *U. woodmasoni* are absent. The medial surface is nearly smooth, lacking rugosities, foramina, and canals. A shallow groove traverses the dorsal portion of the medial process. The anterior end of this groove is indistinct, but it terminates posteriorly at the anterior edge of the posterior notch.

In lateral view, the articulation area for the olfactory process of the frontal is distinct and terminates anteriorly at a weak crest (Fig. 12H). The prefrontal shelf is rounded instead of triangular and is offset only slightly from the dorsal surface of the nasal. Individuals vary in the lateral extent of this process, but its structure is always the same.

In TMM M-10027 a tiny foramen occurs on the anterior end of the right nasal. No other foramina are present.

### Prefrontal

The prefrontal contacts the frontal posteromedially, nasal and septomaxilla anteriorly, maxilla ventrally, and palatine posteroventrally. A distinct frontal process is oriented posteriorly, and in some specimens may contact the anterior tip of the supraorbital process of the parietal, but this varies individually rather than among species. The bone is concave medially, with a smooth medial surface and a broad, rounded lateral surface. The posterior surface of the prefrontal extends medially to form the anterior wall of the orbit. A distinct maxillary process, composed of lateral and medial foot processes, projects ventrally from the main body of the bone and articulates with the ascending process of the maxilla.

#### *Uropeltis woodmasoni*

**Figure 13:**
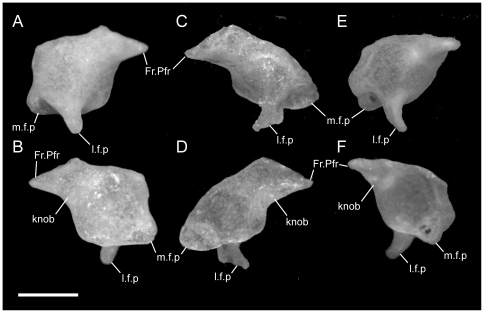
Disarticulated prefrontals. Anterior is to the left in A,D,E; anterior is to the right in B,C,F; scale bar = 0.5 mm. A–C from the left side of the skull in *U. woodmasoni* (TMM M-10001); D–F from the right side of the skull in *U. melanogaster* (TMM M-10045); and G–I from the left side of the skull in *B. rhodogaster* (TMM M-10027). A,C,E in lateral view and B,D,F in medial view. Fr.Pfr = frontal process of prefrontal; knob = medially projecting knob at base of frontal process of prefrontal; l.f.p = lateral foot plate; m.f.p = medial foot process.

In *U. woodmasoni*, the main body of the prefrontal is approximately as long (anteroposteriorly) as it is tall (dorsoventrally). When viewed laterally, the frontal process of the prefrontal overlaps the frontal posteromedially, fitting into a groove in the lateral surface of the frontal and overlying the preorbital ridge. In the disarticulated element, a knob-like process is visible extending medially from the main body of the bone, at the base of the frontal process (barely visible as a dull spot in Fig. 13B). This medial process slots into a relatively large groove dorsal to the lateral frontal flange on the frontal. In two of the articulated specimens (TMM M-10007, -10008) the frontal process of the prefrontal and the supraorbital process of the parietal appear to contact on both sides, but careful examination under high magnification reveals that soft tissue intervenes between them, as does a small process on the frontal. In CT scans, the separation is clear (Fig. 9), and the appearance (or lack of it) in dry skulls is likely a result of differential drying of specimens. In other specimens the processes closely approach one another, but do not have a deceptive appearance of contact.

In lateral view, the dorsal edge of the prefrontal slopes upward anteriorly from the posterior tip of the frontal process until the edge reaches a peak at the junction between the nasal and frontal (Fig. 2A). In articulated skulls, that apex may be rounded or more sharply angled; the disarticulated element forms a clear angle in that region (Fig. 13A). Anterior to that apex, the dorsal edge curves and slopes downward while maintaining contact with the nasal anteriorly. At the junction with the nasal and the septomaxilla, the anterior edge of the prefrontal drops off abruptly and contacts the septomaxilla along a vertical suture. The anteroventral margin of the prefrontal, which forms the medial foot of the maxillary process [19], is overlapped laterally by the ascending process of the maxilla. Unlike the condition in *Anomochilus*, no gap separates the medial foot process and the body of the prefrontal [49]. A distinct, posterolaterally oriented finger-like process in *U. woodmasoni* (Fig. 13A) corresponds to the lateral foot process of the maxillary process [19]; the finger-like process overlaps the posterior margin of the ascending process of the maxilla. The posteroventral margin of the prefrontal, between the lateral foot and frontal processes, is squared, giving the body of the frontal an overall diamond shape.

Medial to the lateral foot process of the prefrontal, a small lacrimal fenestra is completed by the dorsal surface of the palatine and positioned deep in a corner formed between the lateral foot process and the body of the prefrontal. The prefrontal overlaps the dorsolateral surface of the palatine, but the lateral foot process of the prefrontal does not extend past the posterior extent of the lateral process of the palatine.

#### *Uropeltis rubromaculata*

The prefrontal is similar to that of *U. woodmasoni*, but has a relatively larger frontal process that is also larger than the lateral foot process. In lateral view the rounded inflection in the anterodorsal margin occurs just posterior to the junction with the nasal and septomaxilla. A clear gap separates the frontal process of the prefrontal and the supraorbital process of the parietal. Additionally, the entire anterior half of the ventrolateral margin (including the medial foot process) is overlapped by the ascending process of the maxilla, so that no part of the prefrontal is visible between the septomaxilla and the ascending process of the maxilla.

#### *Uropeltis melanogaster*

The prefrontal appears anteroposteriorly compressed relative to *U. woodmasoni* (Fig. 13C). In lateral view, the edge of the bone that contacts the nasal is steep, about 15° from vertical. Unlike the condition in *U. woodmasoni*, no distinct anterior peak marks the junction of the prefrontal, nasal, and septomaxilla. The frontal process of the prefrontal is much larger than the lateral foot process. In medial view, the medial foot process is more distinct from the body of the prefrontal in *U. melanogaster* than in *U. woodmasoni* (Fig. 13D). On the right side of TMM M-10032, the lacrimal foramen appears as a distinct notch or groove just dorsomedial to the maxillary process; in our second specimen (TMM M-10045) and in *U. woodmasoni*, the groove is shallow.

#### *Rhinophis blythii*

The contact of the prefrontal with the nasal in lateral view is longer than in *U. woodmasoni*, and the inflection in the shape of the anterodorsal margin occurs ventral to the junction with the nasal and septomaxilla. As in *U. melanogaster*, the lateral surface appears anteroposteriorly compressed, and overall the bone is taller than it is long (Fig. 8A). The frontal process is much larger than the lateral foot process, but does not contact the supraorbital process of the parietal.

#### *Rhinophis drummondhayi*

The prefrontal is not as anteroposteriorly shortened as in *R. blythii*, nor is it as round as in *U. woodmasoni* (Fig. 8B). In this specimen (TMM M-10046) the prefrontal and parietal closely approach, but do not actually contact, one another. The rounded inflection of the anterodorsal margin is located at the junction with the nasal and septomaxilla.

#### *Rhinophis philippinus*

A broad but short frontal process and a dorsal margin that contacts the frontal for a longer distance than in other taxa gives the prefrontal a square or rhomboidal appearance in lateral view (Fig. 8C). The rounded inflection of the anterodorsal margin occurs at the junction with the nasal and septomaxilla, and the lateral foot process is small, rounded, and stubby. In TMM M-10037 the parietal and prefrontal meet, whereas in TMM M-10038 they do not.

#### *Rhinophis homolepis*

The prefrontal is nearly as long as it is tall, giving it a more rounded appearance. It appears to lack a distinct inflection along the anterodorsal margin, which slopes gradually downward anteriorly. The frontal process is larger than the lateral foot process and clearly meets the supraorbital process of the parietal (Fig. 8D). The contact with the nasal is long, but the suture with the septomaxilla is relatively shorter than in other taxa examined.

#### *Brachyophidium rhodogaster*

The prefrontal is as long as it is tall, and the maxillary and frontal processes are of equal size (Fig. 13E). Although this condition is similar to that in *U. woodmasoni*, disarticulated prefrontals of *B. rhodogaster* can be distinguished from those of *Uropeltis* and *Rhinophis* because those of *B. rhodogaster* have a consistently rounded shape along the anterior and posterior margins, and more bone mass directed anteroventrally. *Uropeltis woodmasoni* is more variable and can approach that condition, but disarticulated specimens generally have a distinct angle or inflection along both the anterior and posterior margins giving the prefrontal a triangular or diamond-shaped appearance when viewed posterolaterally. Specimens of *U. melanogaster* also have a squared posteroventral margin, unlike the condition in *B. rhodogaster* (Fig. 13).

In lateral view of articulated skulls of *B. rhodogaster*, the inflection in the visible anterior margin is rounded and occurs posterodorsal to the junction with the nasal and septomaxilla (Fig. 2C). In three of the articulated specimens (TMM M-10011, -10013, -10015) the supraorbital process of the parietal and the frontal process of the prefrontal meet, but the supraorbital process is longer and thinner than in the other genera. It is difficult to tell if the contacts are bone-bone or if there is more connective tissue or calcified cartilage. In two specimens (TMM M-10017, -10020) the prefrontal and parietal do not touch, and in two others the elements meet on one side only (left of TMM M-10014, right of TMM M-10018), although in both cases the observed condition may be a result of damage or displacement. One specimen (TMM M-10027) has a foramen centered in the middle of the articulation facet for the ascending process of the maxilla. The medial foot process is more distinct than in *U. woodmasoni* and *U. melanogaster* because in *B. rhodogaster* the process extends further anteroventrally and is recessed medially from the body of the bone (Fig. 13E,F).

### Frontal

**Figure 14:**
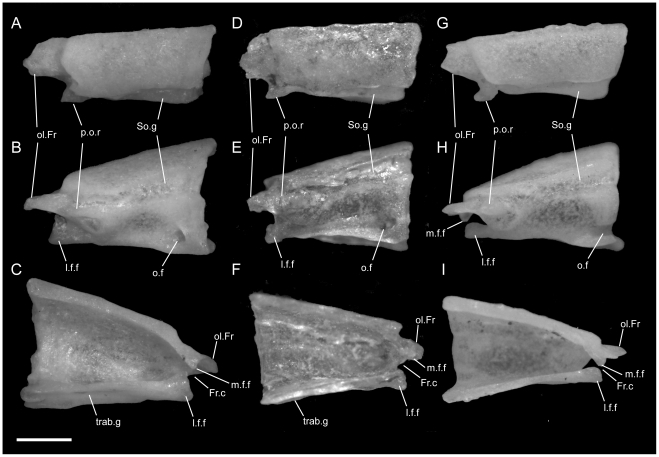
Disarticulated frontals. Anterior is to the left unless noted; scale bar = 0.5 mm. All elements from the left side of the skull. A–C from *U. woodmasoni* (TMM M-10001); D–F from *U. melanogaster* (TMM M-10045); and G–I from *B. rhodogaster* (TMM M-10022). A,D,G in dorsal view; B,E,H in lateral view; and C,F,I in medial view (anterior is to the right). Fr.c = frontal canal; l.f.f = lateral frontal flange; m.f.f = mesial frontal flange; o.f = optic foramen; ol.Fr. = olfactory process of frontal; p.o.r = pre-orbital ridge of frontal; So.g = groove for supraorbital process of parietal; trab.g = groove for cartilaginous portion of crista trabecularis.

The frontal contacts the nasal anteriorly, palatine anteroventrally, parietal posteriorly, sphenoid posteroventrally, and contralateral frontal medially. Contact with the palatine may be made anterolaterally in dried skulls, although CT scans of an alcohol preserved *P. aureus* showed that a small gap filled with soft tissue intervenes [19]. The frontal is broadly exposed in dorsal and lateral views of articulated skulls. The dorsal surface is smooth and convex. The dorsal and lateral portions are separated by a pronounced supraorbital process ( = postfrontal process of [32]) of the parietal, and a less pronounced frontal process ( = supraorbital process of [55]) on the posterodorsal edge of the prefrontal. Contact between those processes varies within and among taxa, and a small portion of the lateral wall of the frontal may be visible between them. The paired frontals meet in an abutting articulation along the midline, and the contact between them is straight and never fused. The articulation areas with the nasal, parietal, and (to a lesser extent) prefrontal are associated with extensive soft-tissue that is clearly visible in the articulated skull (Figs. 5,6). Two distinct articulation facets occur anteriorly in dorsal view. A wide anterior olfactory process [55] (="transverse frontal ridge” of [17], [33]) inserts into the posterior portion of the nasal, and the medial edge of the process turns ventrally to form the mesial frontal flange [33]. Anterolaterally, the preorbital ridge is distinct and, in articulation, is connected to a smaller triangular flange of bone on the posterolateral side of the nasal. Posteriorly, a short extension of the dorsal surface overlaps a narrow shelf on the anterior edge of the parietal. Ventrally, the anterior end of the frontal forms a distinct process, the lateral frontal flange, the medial edge of which curves dorsally to approach the mesial frontal flange. The mesial and lateral frontal flanges do not meet, and a persistent transverse mesial gap is present [33]. The medial portion of the body of the frontal is hollowed and houses the olfactory tracts of the brain [33], forming a crescentic shape in posterior view. The concavity narrows anteriorly and ends at a relatively small opening, the anterior end of the frontal canal (the margins of which are delineated by the mesial and lateral frontal flanges; Fig. 14).

#### *Uropeltis woodmasoni*

The anterior end of the dorsally exposed portion of the frontal varies in shape. In some specimens the anterior exposure appears smooth (e.g., TMM M-10008, -10021), but in others it appears distinctly crenulated along most of its length (e.g., TMM M-10007), is shallowly notched laterally (e.g., left side of TMM M-10006), or more deeply notched and yielding the appearance of anteriorly directed lateral processes (e.g., TMM M-10005, right side of TMM M-10006). The posterodorsal exposure is also somewhat variable. The dominant condition is nearly straight with a slight posterior convexity (e.g., TMM M-10008), but in one specimen (TMM M-10007) the posterior portion is concave posteriorly. In dorsal view of the articulated skull, the lateral edge of the dorsal exposure is emarginated along the length of the supraorbital process of the parietal (Fig. 5A). At the level of the anterior tip of the supraorbital process, the frontal expands again laterally, marking the posterior contact with the prefrontal dorsally. Just ventral to that expansion, a lateral projection of bone (hidden from view in the articulated skull) marks the junction of two distinct grooves that traverse the dorsolateral surface of the frontal (Fig. 14A,B). The anterior groove, for the frontal process of the prefrontal, is oriented anteroposteriorly and is underlain by a narrow shelf of bone marking the posterior extreme of the preorbital ridge. The posterior groove is for the supraorbital process of the parietal, and is inclined posterodorsally from its anterior end. The two grooves are confluent along a bend ventral to the lateral bony projection (Fig. 13B). In dorsal view, the groove that supports the supraorbital process possesses posteriorly a low process or knob on its dorsolateral surface.

A distinct optic foramen is situated ventrally on the posterolateral surface of the frontal, and is contained entirely within the frontal (Fig. 13B). Anterior to that foramen, a low shelf extends anteriorly and then turns ventrally in TMM M-10021. At the anterior extent of this shelf, a narrow, shallow groove marks the frontal contribution to a small fenestra located at the junction between the frontal, prefrontal, and palatine (visible in articulated skulls). The shelf and narrow groove are less distinct in a smaller individual (TMM M-10001; Fig. 13B). Anterodorsal to that narrow groove, a wider groove extends anterodorsally a short distance to terminate at a notch between the olfactory process and the lateral frontal flange. The larger groove, along the base of the lateral frontal flange, receives the medial process of the prefrontal. In anterolateral view, a small tubercle is visible on the dorsolateral edge of the lateral frontal flange.

In articulation, the ventral margin of the frontal in lateral view is gently inclined anteriorly, following the inclination of the palatine on which it sits, while the posterior margin is gently convex and curves around the anterolateral wall of the parietal. In the disarticulated element, the ventral surface is excavated into a dorsally convex groove that traverses most of the anteroposterior length of the bone (Fig. 14C). The lateral and medial margins of the groove are marked by distinct, elongated crests. The groove is widest posteriorly, tapering anteriorly to terminate just posterior to a short, blunt, anterior process that forms the ventral portion of the notch (mentioned above) beneath the olfactory process. The groove is bisected for a short distance at its posterior end by a ventrally projecting crest that delimits a somewhat wider medial portion of the groove and a narrower lateral portion. In the articulated skull, the medial portion is underlain by the parasphenoid rostrum of the sphenoid. The narrower lateral portion of the groove accommodates the cartilaginous trabecula cranii that extends forward from the ossified base of the crista trabecularis [17]. The ossified segment of the crista trabecularis ends just anterior to the ventrolateral part of the frontal-parietal suture, at about the level of the optic foramen (Fig. 11B).

#### *Uropeltis rubromaculata*

The shape, structure, and associations of the frontal in the articulated skull are similar to those outlined for *U. woodmasoni*, although the crista trabecularis ends posterior to the frontal-parietal suture. Disarticulated material is not available.

#### *Uropeltis melanogaster*

The frontals in the two specimens of *U. melanogaster* resemble one another in overall shape, but show less lateral curvature in dorsal view than do those in *U. rubromaculata* and *U. woodmasoni* (Fig. 14D). The olfactory process in both *U. melanogaster* specimens is shorter and stockier than in *U. woodmasoni* (Fig. 14D,E), and in our smaller specimen (TMM M-10032) the process is separated from the preorbital ridge by only a shallow notch, whereas in TMM M-10045 a deep gap separates the two (Fig. 14D). In lateral view, TMM M-10032 lacks a distinct shelf underneath the supraorbital process of the parietal. The ventral trough is not bifurcated posteriorly in either specimen. The tubercle on the lateral frontal flange is developed only as a small nubbin in TMM M-10032. In all *Uropeltis* species that we examined, the optic foramen is enclosed entirely within the frontal, although the bridge of bone separating the foramen from the posterior margin of the frontal is narrower in *U. melanogaster* than in *U. woodmasoni*.

#### *Rhinophis blythii*

*Rhinophis blythii* has proportionately wider frontals than all three *Uropeltis* species we examined (Fig. 6A); in dorsal exposure the combined frontals thus appear more rounded than in *U. woodmasoni*. The contacts between bones are the same, although in *R. blythii* these contacts are always more rounded. The complications of evaluating the association of the posterolateral frontal (i.e., ventrolateral suture with parietal) and the ossified base of the crista trabecularis (character 6 of [17]) are exemplified by *R. blythii*. In our specimen the frontoparietal suture ventral to the supraorbital process of the parietal is somewhat sinuous, but essentially vertically oriented. The ossified base of the crista trabecularis approaches that suture from a strongly angled posteroventral orientation, such that a line extended from the suture would fail to contact the dorsal portion of the base but would intersect its ventral edge. This termination point of the crista is probably best interpreted as occurring at the level of the suture. The fenestra at the junction of the frontal, prefrontal, and palatine is considerably larger in *R. blythii* (TMM M-10030) than in the three *Uropeltis* species and is extended (especially on the left) into an open fissure that separates the dorsal surface of the palatine from the ventral surface of the prefrontal.

#### *Rhinophis drummondhayi*

*Rhinophis drummondhayi* resembles *U. woodmasoni* in the length and breadth of the frontals, but the lateral curvature is not as pronounced in dorsal view, and the contact with the prefrontal is curved as in *U. rubromaculata*. The crista trabecularis ends a short distance anterior to the ventrolateral frontal-parietal suture.

#### *Rhinophis homolepis*

The frontals are proportionately wider and shorter than in the *Uropeltis* specimens. The contacts in the articulated skull are the same, although sharper and more angled than in *R. blythii*. In dorsal exposure, a small, pointed process appears to jut into the junction of the frontal with the prefrontal and supraorbital process of the parietal and may occur because the prefrontal and supraorbital process are in contact. A similar, but narrower, surface extends into the junction between frontal, nasal, and prefrontal. The crista trabecularis ends at the frontal-parietal suture.

#### *Rhinophis philippinus*

Both specimens resemble *U. woodmasoni* in their frontal proportions, but as in the other *Rhinophis* species we examined, the frontals show much less mediolateral tapering in dorsal view. The crista trabecularis ends just anterior to the frontal-parietal suture. In all *Rhinophis* species examined the optic foramen was contained within the frontal. In TMM M-10038, the palatal bones are disarticulated, and it is clear that the posterior bifurcation of the ventral groove is absent. In TMM M-10037 the frontal process of the prefrontal and the supraorbital process of the parietal are in contact.

#### *Brachyophidium rhodogaster*

The frontals are proportionately longer and narrower than in the *Uropeltis* or *Rhinophis* specimens we examined, and also exhibit less lateral curvature in dorsal view, giving the elements a rectangular appearance in dorsal exposure (Figs. 5C,14G). Two specimens have asymmetric frontals, with the right being shorter than the left in dorsal exposure (TMM M-10013, -10015). The shelf that supports the supraorbital process is narrow and is well developed only posteriorly, continuing anteriorly as a weak ridge (Fig. 14H). There is no knob at the posterior end of the shelf. In most specimens, the opening for the optic nerve is a fenestra, formed as an open canal on the posterior ventrolateral surface of the frontal that is closed posteriorly by the parietal (Fig. 14H). In one specimen (TMM M-10017) the optic foramen is enclosed entirely within the frontal on the right side only, but it is still located farther posteriorly than in the *Uropeltis* and *Rhinophis* specimens we examined. Articulated specimens display an anterior foramen visible in lateral view at the junction of the frontal, prefrontal, and palatine; this is similar to the condition in *Uropeltis* and *Rhinophis*. However, if the prefrontal is removed the foramen appears to be formed mainly by the frontal and palatine, with the prefrontal simply overlapping those elements. In *B. rhodogaster* the dorsal lamina is slanted laterally. The shape of the preorbital ridge in dorsal view is variable within our sample. It may have a rounded triangular shape (e.g., TMM M-10016, -10023), a sharply triangular shape (e.g., TMM M-10024, left side of TMM M-10026) blocky with squared edges (e.g., left sides of TMM M-10027 and TMM M-10022); or triangular with a shallow (e.g., right side of TMM M-10026) or deep (e.g., right side of TMM M-10022) notch on the posterior edge. The ridge is offset from the olfactory process by a large gap, although the two are connected via a narrow shelf that extends along the edge just ventral to the dorsal exposure of the bone.

The olfactory process is variable in shape and may be triangular and pointed (e.g., TMM M-10016) or broad and square (e.g., TMM M-10023). One specimen (TMM M-10024) possesses an additional anterior process on the left side, ventromedial to the olfactory process; the additional process probably articulated with the nasals or the contralateral frontal. In anterior view, the frontal canal is completely closed by the mesial and lateral frontal flanges in most of our specimens (TMM M-10016, -10023, -10026, -10027). The flanges meet only on the right side in TMM M-10022, and are widely separated in our smallest specimen (TMM M-10024). The ventral groove is well developed and wide in *B. rhodogaster*, but is not bifurcated posteriorly. On the left side of TMM M-10024 the posterior extent of the medial crest of the ventral groove extends farther posteriorly so that it is visible in dorsal view. This is not the case in any other specimens observed. In all specimens the crista trabecularis ends at the frontoparietal suture.

### Parietal

**Figure 15:**
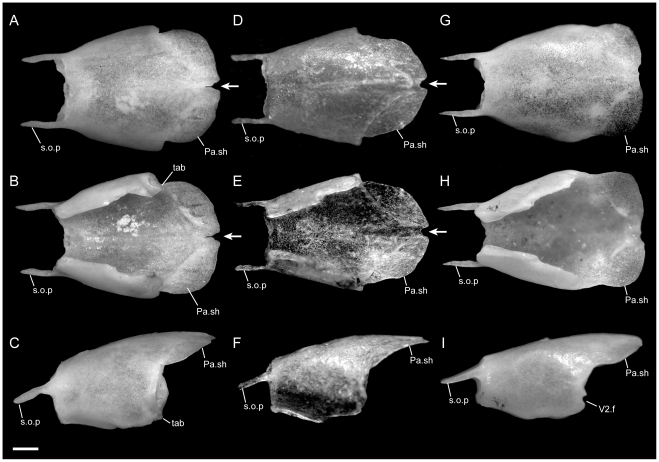
Disarticulated parietals. Anterior is to the left; scale bar = 0.5 mm. A–C from *U. woodmasoni* (TMM M-10001); D–F from *U. melanogaster* (TMM M-10045); and G–I from *B. rhodogaster* (TMM M-10022). A,D,G in dorsal view; B,E,H in ventral view; and C,F,I in lateral view. Arrow points to slit-like opening along posterior midline of parietal. Pa.sh = parietal shelf; s.o.p = supraorbital process of the parietal; tab = tab-like process that articulates with otic region; V2.f = notch that contributes to V2 foramen.

The parietal is a smoothly rounded, dorsally convex, midline element. At its anterolateral margin, a fingerlike supraorbital process extends anteriorly onto each frontal. In addition to the frontal, the parietal contacts the fused braincase complex posteriorly and ventrally, and occasionally the prefrontal anteriorly (via the supraorbital process). The parietal closely approaches the pterygoid laterally, but soft tissue prevents contact. In all uropeltids we examined, only a single, unpaired parietal is present, although previous authors reported that incomplete fusion of the parietals is visible in some specimens of *Rhinophis* and *Uropeltis* [17]. It seems likely that those reports were based on the narrow, slit-like opening along the posterior midline of the parietal visible in some specimens (e.g., Fig. 15).

#### *Uropeltis woodmasoni*

The parietal (excluding the supraorbital process) takes up about one-third of the total skull length and is anteroposteriorly longer than it is wide or tall (Figs. 2A;15A,C). In dorsal view, a low, weak sagittal crest terminates anteriorly at a small, shallow depression formed by a zone of thin, fragile bone. The depression is located along the midline, at about one-quarter of the distance from anterior to posterior. In some specimens (TMM M-10001, -10003, -10004, -10021) the anterior edge of the depression is noticeably thickened or inflated (barely discernible as a rounded bump in Fig. 15C), and in one specimen (TMM M-10007) a circular hole is punched through the thin floor of the depression.

The anterior edge of the parietal underlaps the frontal. In disarticulated specimens the frontal shelf is rough and irregular (Fig. 15A). In articulated specimens, the long, narrow supraorbital processes have a blunt, rounded tip and may or may not reach the prefrontals (see prefrontal description for variation). When viewed dorsally, the supraorbital processes frame the frontals and create a U-shaped frontal-parietal suture (Fig. 5A). Posterior to the base of the supraorbital processes, the parietal widens rapidly, reaching its greatest width immediately anterior to the otic region (Fig. 15A). At roughly the same point, the parietal, when viewed laterally, attains its greatest height (Fig. 15C).

In lateral view, contact with the frontals is a straight, vertical suture from the sphenoid up to the supraorbital process (Fig. 2A). Posterior to the frontal, the parietal rests on top of the sphenoid portion of the braincase. The suture between those elements is smooth and roughly horizontal, although it gradually declines ventrally from anterior to posterior, before leveling off posteriorly. When the parietals are disarticulated, the ventral surfaces of the lateral walls are slightly expanded into flat, narrow shelves that are angled dorsomedially (Fig. 15B). When viewed anteriorly, a groove is visible along the dorsal portion of the shelves. The groove ends posteriorly at the notch for the vidian canal, which is located along the sphenoid-parietal suture and completed by the parietal, at the level of the bifurcation in the pterygoid. The pterygoid is located just lateral to the long parietal-sphenoid suture and closely approaches the parietal at a point posterior to the base of the ectopterygoid process of the pterygoid.

Contact with the sphenoid ends posteriorly at the fusion of the sphenoid to the otic capsules. At that point the parietal articulates with the otic region along a straight, smooth, and mostly vertical suture ventrolaterally (Fig. 2A). Posterolaterally, the parietal overlaps the otic region with an external suture angled posterodorsally. The parietal actually underlies the otooccipital (the entire fused portion of the braincase) along that vertical margin, and when the two elements are separated a small tab-like process at the posteroventral corner of the parietal secures articulation with the braincase (Fig. 15B,C). The process is just medial to, and is parallel with, the lateral wall of the parietal and projects posteriorly. It extends vertically from the ventral margin of the parietal to a level just dorsal to the foramen for the maxillary branch of the trigeminal nerve (cranial nerve [CN] V_2_) foramen and includes a notch for the passage of soft tissue through that opening. The CN V_2_ foramen is located along the vertical suture, at the level of the quadrate, and is completed by the anterior margin of the otic region (Fig. 2A). In TMM M-10001 (right side only) and in TMM M-10009 (both sides) the foramen is shifted ventrally and approaches the parietal-sphenoid suture.

The vertical suture with the braincase complex terminates dorsally at a broad shelf of the parietal that extends posteriorly to overlie the anterodorsal half of the otic capsules, in the supraoccipital region. The lateral edges of the shelf are straight and horizontal and originate at a right angle to the vertical suture with the otic region. In other taxa the posterior extent of the shelf is composed of two short, oblate lobes, but in *U. woodmasoni* the lobes are broad and meet along the posterior midline to form a rounded, smooth, and upswept posterior margin. In three specimens (TMM M-10001, -10004, -10009), the posteriormost region of the shelf butterflies into a small v-shaped notch along the midline (Fig. 15A). In disarticulated specimens the ventral surface of the posterior shelf is rough and irregular along the area where it articulates with the otic capsules (Fig. 15B).

#### *Uropeltis rubromaculata*

The supraorbital processes of the parietal are proportionately narrower and straighter than in *U. woodmasoni*, and also are less tapered anteriorly. In dorsal view, the sagittal crest is more strongly developed in *U. rubromaculata*, but still weak, and the crest ends at a circular, roughened patch of bone instead of terminating at a depression. The posterior margin of the parietal has a prominent trilobed appearance. In lateral view, the opening for the CN V_2_ is almost entirely within the braincase, but the anterior margin is completed by the parietal (Fig. 2B).

#### *Uropeltis melanogaster*

The supraorbital processes are thin, fragile, and susceptible to curling away from the skull when dried (Fig. 15D–F). In dorsal view, the parietal of TMM M-10032 is nearly as wide as it is long, giving the bone a circular shape that is different from the morphology of *U. woodmasoni*, *U. rubromaculata*, and the second *U. melanogaster* (TMM M-10045), all of which have a parietal that is longer than it is wide (Fig. 15). TMM M-10032 also differs in the shorter length of the shelf along the ventrolateral margin, which fails to reach the vidian canal, and in its complete lack of a sagittal crest. In place of a depression, a flat, smooth, triangular area originates at the anterior margin and tapers posteriorly. TMM M-10045, which has a rounded sagittal crest, also exhibits a triangular flat spot, which occurs where the sagittal crest forks anteriorly (Fig. 15D). Posterior to each branch of the fork is a narrow depression, the anterior rim of which is thickened. In ventral view, the shelf along the ventrolateral margin of the parietal reaches the vidian canal as in the other *Uropeltis* species examined. In both specimens, the posterior shelf is different from that of *U. woodmasoni* and *U. rubromaculata*. The portion over the otic region consists of two oblate, narrow shelves that do not meet along the midline and a wide, square notch is between the shelves (Fig. 15D). Additionally, neither specimen has a tab-like process for interlocking with the braincase along the vertical parietal-braincase suture, although both exhibit a smooth anterodorsal surface for support of the frontals. *Rhinophis blythii*

The supraorbital processes are straight and slender, with little tapering and a shorter length relative to the other species we examined (Fig. 8A). Dorsally the sagittal crest is stronger than in the three *Uropeltis* species examined and ends anteriorly at a weakly depressed, roughened, irregular spot. The two lobes of the shelf dorsal to the otic region are relatively short, and there is a small, squared notch at the posterior midline. In lateral view the CN V_2_ opening is almost entirely within the braincase, but a small portion of the parietal completes its anterior edge.

#### *Rhinophis drummondhayi*

The supraorbital processes are robust and similar to those of *U. woodmasoni*, but the sagittal crest is stronger and terminates anteriorly at a triangular, roughened, shallow depression. There is only a small posterior notch along the midline. In lateral view, the majority of the CN V_2_ foramen is in the braincase, and the parietal completes only the anterior-most portion.

#### *Rhinophis homolepis*

The supraorbital processes are fairly straight and are proportionately wider than in *R. blythii*. There is no sagittal crest or depression in dorsal view. The posterior margin is like that of *U. woodmasoni*, being smoothly rounded and carrying only a small V-shaped notch along the posterodorsal midline of the area overlying the otic region. In lateral view the CN V_2_ opening is located more equally within the parietal and the braincase. Additionally, this specimen possesses a hole in the left-lateral side of the parietal that appears to be a result of mechanical damage, as indicated by multiple tiny scratches in the vicinity of the hole.

#### *Rhinophis philippinus*

The supraorbital processes are straight but deflected slightly anteroventrally (Fig. 8C). They do not taper greatly, but end as a squared anterior tip. The sagittal crest and associated anterior depression are weaker in TMM M-10038 than in TMM M-10037, and the latter also exhibits thickening of the anterior margin of the depression. In lateral view, two-thirds of the CN V_2_ foramen is within the braincase, and the parietal completes the anterior third. In both specimens, the height of the skull is proportionately lower than in other *Uropeltis* and *Rhinophis* species examined, and in TMM M-10037 the highest point occurs just anterior to the posterior margin of the otic shelf, far posterior to the widest region of the skull (Fig. 8C).

#### *Brachyophidium rhodogaster*

The supraorbital processes are straight and taper anteriorly to a sharp point, unlike the blunt or rounded condition of the other species (Figs. 2C,15I). The parietal is only slightly longer than it is wide (excluding the supraorbital process) in dorsal view (Fig. 15G), although it does not have the spherical appearance of the smaller *U. melanogaster* specimen (TMM M-10032). Five specimens (TMM M-10013, -10014, -10016, -10018, -10023) of *B. rhodogaster* have an extremely weak sagittal crest; it is absent in all other specimens. Whether or not there is a sagittal crest, an anteriorly thickened depression of varying depth is present at the center of the dorsal surface of the parietal (Fig. 15I, note thickened ridge). In most specimens the widest and highest points of the parietal occur in the same plane as the dorsal depression, but in three (TMM M-10016, -10019, -10022) these extrema occur farther posteriorly, at or near the parietal-braincase suture. In *B. rhodogaster* the two lobes of the posterior otic shelf are short and relatively widely separated. The posterior margins of the lobes are smooth, and there is no notch at the posterior midline, except in TMM M-10023 where the lobes nearly meet at the midline. That specimen also possesses an anomalous tiny foramen located on the left side only, dorsal to the sphenoid-parietal contact and posterior to the frontal-parietal suture. Another specimen, TMM M-10027 has three tiny foramina perforating the left otic lobe.

In lateral view the anterolateral margin completes the optic foramen along the frontal-parietal suture, and the posterolateral margin completes the CN V_2_ opening (Fig. 2C). In most individuals the CN V_2_ foramen is shared equally between the parietal and the braincase, but in three (TMM M-10014, -10018, -10020) the parietal only completes the anterior quarter of the opening. In disarticulated specimens, the anterior margin lacks the shelf or lip that underlies the frontal as seen in the *Uropeltis* species. Posteriorly, there is no tab-like process associated with the vertical parietal-braincase suture, but that area is roughened and irregular from articulation and does have a notch that contributes to the opening for CN V_2_ (Fig. 15H,I). Ventrally, the ventral surfaces of the lateral walls have a flat surface extending posteriorly to the vidian canal (Fig. 15H). The dorsal surface of the shelf has a groove leading to the vidian canal, as in *U. woodmasoni*.

### Vomer

The vomer contacts the premaxilla anteriorly, maxilla laterally, septomaxilla dorsally, and palatine posteriorly. The paired vomers share a straight, midline contact. The body of the vomer contains the vomeronasal opening, which opens laterally. In ventral view, two processes are visible as a result. The anterolateral process forms the anterior margin of the opening and contacts the maxilla, premaxilla, and septomaxilla; the posterolateral process forms the posterior margin and contacts the septomaxilla and palatine.

#### *Uropeltis woodmasoni*

**Figure 16:**
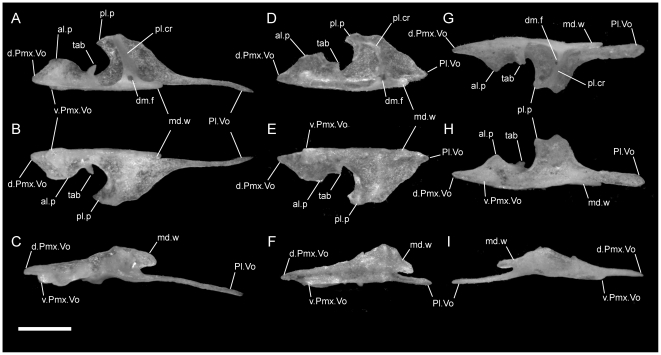
Disarticulated vomers. Anterior is to the left in A–F; anterior is to the right in I; scale bar = 0.5 mm. A–C from the right side of *U. woodmasoni* (TMM M-10001); D–F from the right side of *U. melanogaster* (TMM M-10045); and G–I from the left side of *B. rhodogaster* (TMM M-10022). A,D,G in dorsal view; B,E,H in ventral view; and C,F,I in medial view. al.p = anterior lateral process; d.Pmx.Vo = dorsal premaxillary process of vomer; dm.f = dorsomedial foramen of posterolateral crest; md.w = medial wall; pl.cr = crest on posterolateral process; pl.p = posterior lateral process; Pl.Vo = palatine process of vomer; Pmx.Vo = premaxillary process of vomer; tab = bone tab projecting into vomero-nasal opening; v.Pmx.Vo = ventral premaxillary process of vomer.

**Figure 17:**
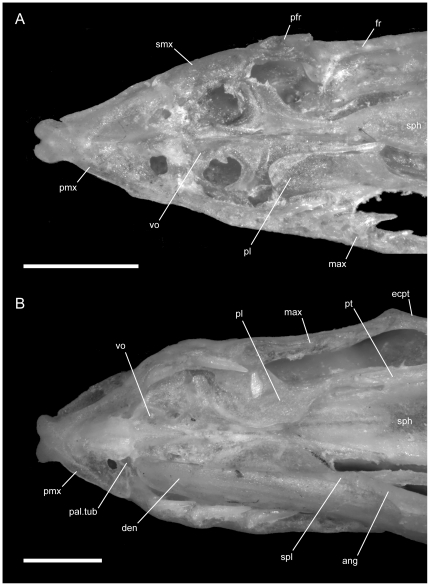
Magnified view of the palate of *Uropeltis*. Anterior is to the left; scale bars = 1.0 mm. (A) *U. woodmasoni*, TMM M-10010. Note missing max, pl, ecpt, and pt on left side. (B) *U. rubromaculata*, TMM M-10028. Note that right lower jaw is present. ang = angular; den = dentary; ecpt = ectopterygoid; fr = frontal; max = maxilla; pal.tub = palatine tubercle of septomaxilla; pfr = prefrontal; pl = palatine; pmx = premaxilla; pt = pterygoid; smx = septomaxilla; spl = splenial; sph = sphenoid region of the otooccipital complex; vo = vomer.

Anteriorly, in ventral view, the triangular premaxillary process (Fig. 16A,B) contacts the medial vomerine process of the premaxilla (Figs. 4A,17A). In disarticulated specimens a second medial process is visible dorsal to the premaxillary process (Fig. 16A–C). That triangular dorsal process is longer than the ventral premaxillary process and overlaps the vomerine process of the premaxilla, so that the vomer clasps the premaxilla. The anterior surface of the ventral premaxillary process may have a rounded, shallow notch or indentation for articulation with complimentary projections of the vomerine process of the premaxilla. When strongly developed, this facilitates interlocking of the two bones.

The anterolateral process of the vomer (Fig. 16B) is oriented lateral to the vomerine process of the premaxilla, and in cases where the septomaxilla does not fill the gap (e.g. TMM M-10004), the process is located posterior to the premaxillary ventral fenestra (Figs. 4A,17A). Just anterior to the vomeronasal opening, the anterolateral process of the vomer may meet the anteromedial process of the maxilla (see maxilla section). The posterolateral process of the vomer is a broad flange (Fig. 16B) that contacts the posterior end of the lateral wall of the septomaxilla and is overlapped by the palatine. The process has a small, pointed projection that curves anteriorly to form the posterolateral margin of the vomeronasal opening. The subcircular vomeronasal openings are large and may cover three-quarters of the ventral surface area (Fig. 16B). The medial margin of the opening possesses a pointed, slender, tab-like process that extends posterodorsally into the nasal passage to weakly contact the septomaxilla. In addition to completing the lateral margins of the vomeronasal openings, the septomaxillae separate the vomers from the maxillae at this point.

Posteriorly, the vomers and maxillae are separated by a gap filled with soft tissue. The posterolateral margin of the vomer narrows rapidly, producing a slender, pointed, posteromedial (palatine) process that is approximately 40% of the total length of the bone (Fig. 16A–C). The anteromedial edge of the palatine overlaps the vomer starting at the base of that process and continues to overlap its anterior half (Fig. 17A). The medial walls of the choanal processes of the palatines descend between the palatine processes of the vomers, separating the vomers posteriorly.

The medial surface of the vomer is smooth and vertically straight, terminating posteriorly in a blunt, tapered process that sits dorsal to the origin of the palatine process (Fig. 16C). Dorsally the vomer has a well-developed, thin crest of bone extending from the medial wall transversely across the center of the posterolateral process (Fig. 16A). Anterior to the crest, the vomer is concave dorsally to contain the vomeronasal organ. Immediately posterior to the point where the crest meets the medial wall, a small, circular foramen pierces the dorsomedial portion of the crest. Ventral to that foramen is a second, smaller foramen that is directed anteroventrally to penetrate the floor of the vomer and open at the ventral part of the medial wall, in ventromedial view. The latter foramen is obscured in articulated specimens, but occasionally a small, medial indentation in the posterior half of the ventral surface of the body of the vomer indicates its presence.

#### *Uropeltis rubromaculata*

In ventral view the anterior edge of the vomer has a short, ventral premaxillary process with a shallow, lateral indentation and farther laterally a tiny, pointed projection that juts between the premaxilla and septomaxilla (Figs. 4B; 17B). The anterior margin of the anterolateral process is posterior to that projection. Compared to *U. woodmasoni*, the anterolateral process is expanded and has a squared appearance. The anterolateral process of the vomer and the anteromedial process of the maxilla do not touch but both are in contact with the overlying septomaxilla. Within the vomeronasal opening, a thin bar of bone separates the smaller, medial half from the lateral half. As in *U. woodmasoni*, *U. rubromaculata* has a long, thin palatine process.

#### *Uropeltis melanogaster*

*S*imilar to *U. rubromaculata*, the anterolateral and premaxillary processes are more squared (Fig. 16D,E). In the smaller specimen, TMM M-10032, the anterolateral, premaxillary, and posterolateral processes all are relatively weakly developed and, other than the medial margin, the vomer appears to form little of the vomeronasal opening. In TMM M-10045, the processes are as well developed as in the other *Uropeltis* specimens examined. In dorsal view there is a crest or ridge as in *U. woodmasoni*, but the ridge folds over to create a convex pouch or cup (Fig. 16D). A small foramen pierces the dorsal portion of the crest, but only a rounded, posteriorly-opened notch occurs ventrally, rather than a complete foramen. The vomer terminates posteriorly in a short, broad, and triangular point (Fig. 16D,E).

#### *Rhinophis blythii*

**Figure 18:**
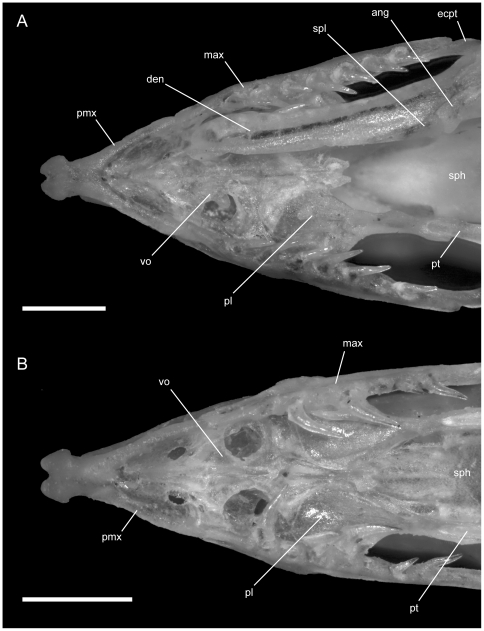
Magnified view of the palate of *Rhinophis*. Anterior is to the left; scale bars = 1.0 mm. (A) *R. blythi*. Note that the left lower jaw is present. (B) *R. drummondhayi*. ang = angular; den = dentary; ecpt = ectopterygoid; max = maxilla; pl = palatine; pmx = premaxilla; pt = pterygoid; smx = septomaxilla; spl = splenial; sph = sphenoid region of the otooccipital complex; vo = vomer.

In anterolateral view the dorsal premaxillary process of the vomer extends anteriorly past the septomaxilla and is visible in the floor of the external naris. In ventral view, the triangularly pointed ventral premaxillary process of the vomer lies lateral to the vomerine process of the premaxilla (Figs.7A; 18A). The medial surface of the premaxillary process is L-shaped and receives the vomerine process of the premaxilla. Posterolaterally, the anterolateral process of the vomer contacts the anteromedial process of the maxilla on the right side, but not the left side, of our specimen (TMM M-10030). The posterolateral process and its anterior projection are broader and rounder than in the other species of *Uropeltis* examined. The posterolateral process is thickened and a small ridge occurs where the process forms the posterolateral margin of the vomeronasal opening. The palatine process is not elongate.

#### *Rhinophis drummondhayi*

The ventral premaxillary process is small and rounded, abuts the vomerine process of the premaxilla, and possesses a shallow indentation laterally (Figs. 7B; 18B). The anterolateral process is squared and meets the anteromedial process of the maxilla. The anterior projection of the posterolateral process forms much more of the vomeronasal opening than in any other species examined. The small, pointed process that projects into the vomeronasal opening originates dorsal to the ventral surface, rather than at it as in the three species of *Uropeltis*, and *R. blythii*. Dorsally, the vomer has a strong medial inflection. The palatine process is not elongate.

#### *Rhinophis philippinus*

**Figure 19:**
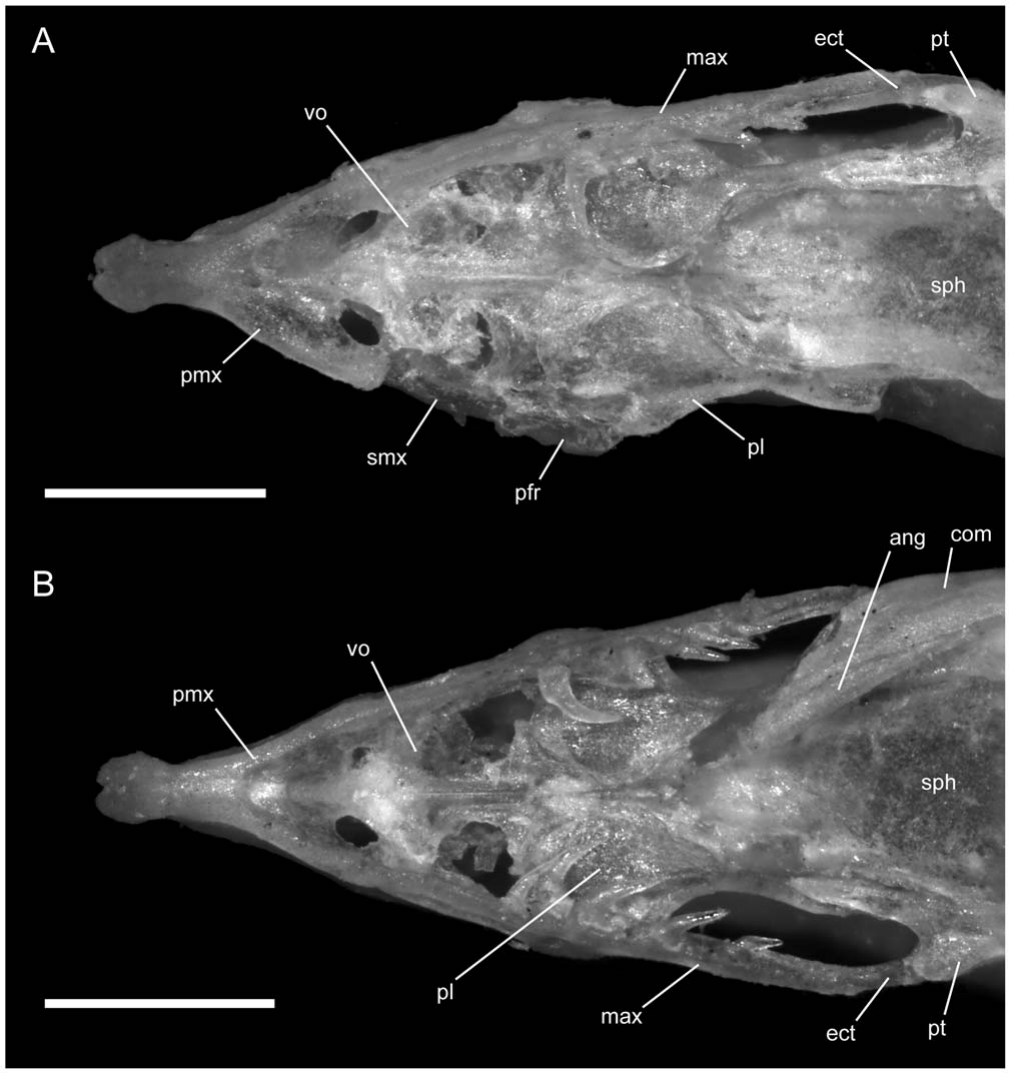
Magnified view of the palate of *Rhinophis*. Anterior is to the left; scale bars = 1.0 mm. (A) *R. philippinus*. Note missing max, ecpt, and pt on right side; (B) *R. homolepis*. Note that right com and ang are present. ang = angular; ecpt = ectopterygoid; max = maxilla; pfr = prefrontal; pl = palatine; pmx = premaxilla; pt = pterygoid; smx = septomaxilla; sph = sphenoid region of the otooccipital complex; vo = vomer.

As in *R. drummondhayi*, the anterolateral process is squared and the small, pointed process that projects into the vomeronasal opening originates dorsal to the ventral surface. In TMM M-10038, which is partially disarticulated, the process located dorsal to the premaxillary process is proportionately shorter than in other specimens. The posterolateral process may barely contact the prefrontal, ventral to the maxilla where the prefrontal, maxilla, and septomaxilla meet; that contact does not occur in other taxa. The palatine process is not elongate (Figs. 7C; 19A).

#### *Rhinophis homolepis*

The anterolateral process is large, broad, and squared in ventral view. It meets the anteromedial process of the maxilla and maintains contact with it along the posterior margin of the latter, moving toward the body of the maxilla. The anterior margin of the vomer is more like that of *U. woodmasoni* than *R. blythii*. It is also similar to those taxa in that the small process that projects into the vomeronasal opening originates on the ventral surface of the bone. As in all other *Rhinophis* species we examined, the palatine process is not elongate (Figs. 7D; 19B).

#### *Brachyophidium rhodogaster*

**Figure 20:**
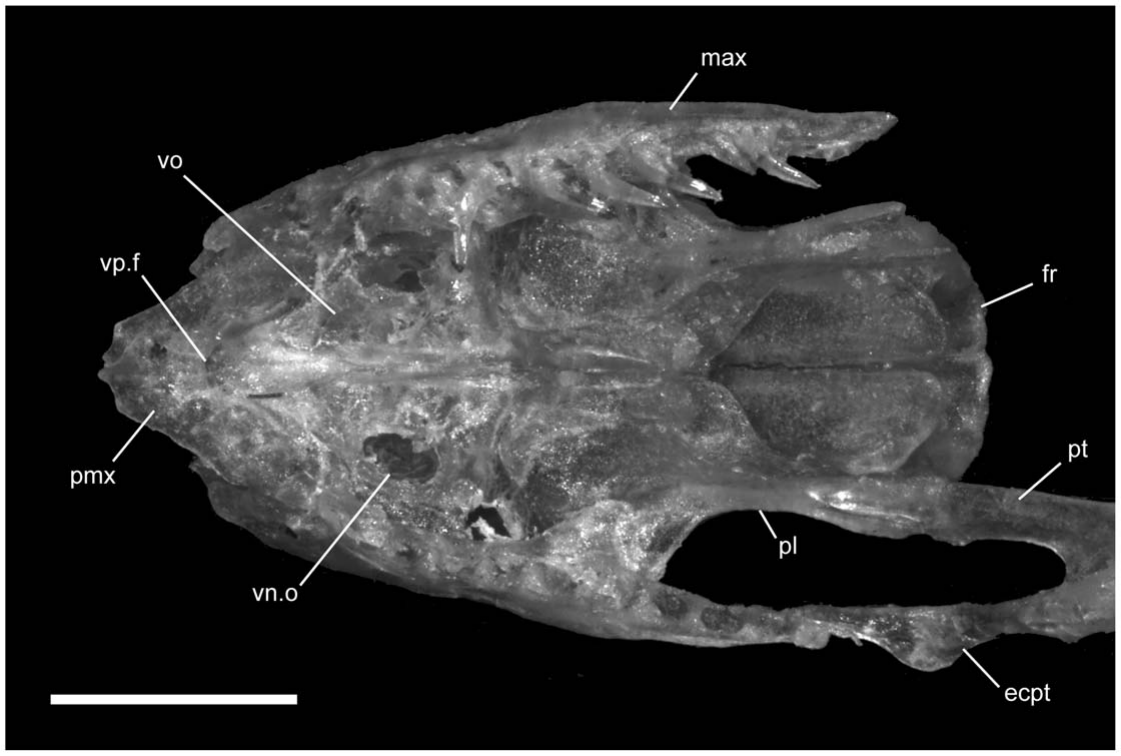
Magnified view of the palate of *Brachyophidium rhodogaster*. Anterior is to the left; scale bar = 1.0 mm. Note that posterior half of skull, including otooccipital region, left ecpt, and left pt, is missing. ecpt = ectopterygoid; fr = frontal; max = maxilla; pl = palatine; pmx = premaxilla; pt = pterygoid; vp.f = ventral premaxillary foramen; vn.o = vomeronasal opening; vo = vomer.

The premaxilla projects between the vomers, and the transverse process of the premaxilla extends to the junction between the maxillae, septomaxillae, and vomers. In ventral view, the anterior tip of the ventral premaxillary process of the vomer is broadly triangular, but lacks an indentation laterally for the premaxilla (Fig. 16H). Relative to the condition in *U. woodmasoni*, the ventral premaxillary process in *B. rhodogaster* is reduced, reaching only the midpoint of the vomerine process of the premaxilla in articulation. There is also a longer dorsal premaxillary process that in clean, articulated specimens is visible through the transparent underlying premaxilla, giving the false impression that the vomers completely underlie the vomerine process of the premaxilla, rather than clasp it. Posterolateral to the premaxillary process, the vomer curves laterally to form a small, pointed anterolateral process (Figs. 4C;16G,H;20). In most specimens the anterolateral process of the vomer and the anteromedial process of the maxilla do not touch, but refer to the section on the maxilla for variation. The posterolateral process is more squared than in the *Uropeltis* and *Rhinophis* species, and most specimens lack an anterior projection. The lateral edge of that process is slightly U-shaped and appears to gently clasp the posterior end of the lateral margin of the septomaxilla. In two specimens (TMM M-10022, -10026), the lateral edge of the process is more strongly developed into a V-shape (Fig. 16G,H). Inside the vomeronasal opening, the tiny, internally projecting process originates at the ventral surface.

The long, posterior palatine process is dorsoventrally compressed (Figs. 4C;16H,I) and much broader than in *U. woodmasoni*. The tapering of the posterolateral margin of the vomer that produces the process is more gradual than in *U. woodmasoni*. In dorsal view, a crest and anterior concavity occur in association with the posterolateral process as in other taxa (Fig. 16G). A single dorsal foramen pierces the posterolateral crest near its origin at the medial wall. Dorsal to the palatine process, a short, tapered, and pointed additional posterior process begins at the medial wall (Fig. 16G,I). In between the two posterior processes, a small, anteroposteriorly directed canal leads into the medial wall. The canal exits at the level of the crest, in the floor of the medial wall and, based on position, may be homologous to the ventral foramen of *U. woodmasoni*.

### Palatine

The palatine contacts the vomer anteriorly, maxilla laterally, septomaxilla anterodorsally, prefrontal laterally, pterygoid posteriorly, and frontal dorsally. The expanded anterior end of the palatine is broadly triangular with a rounded tip. As far as is known, the palatine is edentulous in all uropeltid species except for *Melanophidium wynaudense* [17].

#### *Uropeltis woodmasoni*

**Figure 21:**
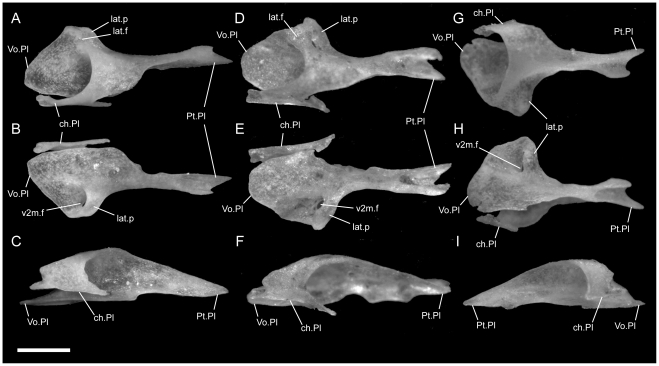
Disarticulated palatines. Anterior is to the left in A–F; anterior is to the right in I; scale bar = 0.5 mm. A–C from the right side of *U. woodmasoni* (TMM M-10001); D–F from the right side of the skull of *U. melanogaster* (TMM M-10045); and G–I from the left side of the skull of *B. rhodogaster* (TMM M-10022). A,D,G in dorsal view; B,E,H in ventral view; and C,F,I in medial view. ch.Pl = choanal process of palatine; lat.f = tiny lateral foramen; lat.p = lateral process of palatine; Pt.Pl = pterygoid process of palatine; Vo.Pl = vomerine process of palatine; v2m.f = foramen for branch of the trigeminal nerve (CN V_2_).

In dorsolateral view, the anterior half of the palatine underlies the prefrontal beginning from the junction between the frontal, prefrontal, and palatine and extending laterally to the lateral extent of the maxillary process of the prefrontal. Posterior to that point, the palatine contacts the dorsal edge of the maxilla. The palatine completes two fenestrae visible in posterolateral view: one at the triple junction with the prefrontal and frontal and a second on the ventral surface of the prefrontal which presumably is for the lacrimal duct. Posteromedial to the latter opening, a large foramen for a branch of CN V_2_ (Fig. 21B) occurs in the lateral process of the palatine. There is also a tiny foramen located ventral to the suture with the frontal, within the lateral surface of the palatine, which is exposed in both disarticulated and articulated specimens (Figs. 2A;21A). Also in lateral view, the palatine contributes to the floor and medial wall of the orbit. The palatine underlies the frontal and the anterior end of the sphenoid until immediately anterior to the frontal-parietal suture, at the optic foramen, where the palatine tapers posteriorly to form the long, slender pterygoid process. As a result of that tapering a large gap exists posteriorly between the palatine and the braincase (Fig. 2A). The posterior tip of the pterygoid process of the palatine clasps the palatine process of the pterygoid. The ventral surface of the tip is emarginated to form a groove, into which the pterygoid slots (Fig. 21B). The groove is asymmetrical, with the medial boundary longer than the lateral one.

In ventral view, the lateral process of the palatine [32] projects anterolaterally towards the posterior margin of the palatine process of the maxilla, by which it is underlapped. In dorsolateral view of articulated skulls, and even more clearly in disarticulated specimens, it can be seen that the lateral ‘process’ is the ventral surface of a loop of bone surrounding the large foramen for CN V_2_ (Fig. 21B). The same structure was described for the disarticulated palatine of *P. aureus* [19]. In *U. woodmasoni*, the loop is oriented vertically and aligned anterolaterally from its origin on the lateral surface of the palatine, positioned at right angles to the ventral floor and the lateral wall of the element. In some specimens the presence of a suture indicates that the loop is formed by closure between a dorsally reaching ventrolateral process and a ventrally reaching dorsolateral process. When disarticulated and viewed ventrally, anterior to the loop and at the exit for the foramen, a broad groove or depression slopes anteroventrally, eventually flattening out with the ventral surface of the palatine. The palatine process of the maxilla articulates with this groove (Fig. 21B).

Anteroventrally, the vomerine process of the palatine underlaps the posterior half of the palatine process of the vomer (Fig. 17A). The choanal process of the palatine, which encloses the choanal passage, ascends dorsally, curving anteromedially from its origination at the ventrolateral surface of the palatine (Fig. 21A,C). That structure gives the palatine the appearance of being curled into a C-shape, with the open portion directed dorsomedially, when viewed posteriorly in articulated specimens. The choanal process terminates in a ventromedial position (Fig. 21B) and would come back into contact with the ventral surface of the palatine if not for the intervening palatine process of the vomer. The medial edge of the ventral surface of the choanal process abuts the palatine process of the vomer (Fig. 4A), and when viewed posteriorly along the midline the narrow, the ventral surfaces of the medial walls of the paired palatines are visible sandwiched between the palatine processes of the vomers. In medial view of disarticulated specimens, the choanal process of the palatine narrows slightly along the medial face, but widens abruptly at its terminus to form an elongate, T-shaped expansion (Fig. 21C). Posteriorly that surface has a curved, pointed projection inclined vertically, while anteriorly there are two pointed projections, one dorsal and one ventral.

#### *Uropeltis rubromaculata*

In dorsolateral view, the wide, anterior portion of the palatine extends laterally only to the lacrimal duct, falling short of the terminus of the maxillary process of the prefrontal. In addition to the tiny foramen visible in this view in *U. woodmasoni*, a second foramen is located at the posterior extent of the contact with the maxilla, near the base of the pterygoid process. When viewed laterally, the tapering of the pterygoid process and the end of contact with the frontal occur anterior to the optic foramen, farther anteriorly than in *U. woodmasoni*. The clasping articulation of the pterygoid process of the palatine with the palatine process of the pterygoid is depicted in Figure 17B.

#### *Uropeltis melanogaster*

The palatine process of the vomer is absent, and the choanal process of the palatine weakly contacts the ventral surface of the palatine. This is partly because the ventral surface, particularly the vomerine process, is flat and expanded relative to that of *U. woodmasoni*, and fills the space that in *U. woodmasoni* is taken up by the vomer (Fig. 21E). The anterior tip of the ventromedial terminus of the choanal process is squared, whereas the posterior tip is a triangular point lacking well-developed projections (Fig. 21D,E). In ventral view of articulated specimens, the lateral process of the palatine is thin and straight, rather than hooked, and is directed anterolaterally. Again, presence of a suture suggests that the loop forming this surface is constructed from closure between two lateral processes (Fig. 21E). The pterygoid process is wider and more robust than in *U. woodmasoni* and *U. rubromaculata*.

#### *Rhinophis blythii*

As in *U. rubromaculata*, two foramina occur on the lateral surface of the palatine (not depicted in line drawings). In dorsolateral view, the anterior portion of the palatine extends laterally to the end of the maxillary process of the prefrontal, at which point the palatine contacts the maxilla and remains in contact with the latter until the origin of the pterygoid process. Tapering of the pterygoid process of the palatine begins immediately anterior to the frontal-parietal suture and the optic foramen. The ventral surface of the pterygoid process of the palatine exhibits a well-developed short, broad flange that is directed medially (Fig. 18A). In ventral view, this specimen appears to have a separate lateral process of the palatine because the overlying loop is not closed. The robust lateral process is straight, rather than hooked, extends anterolaterally, and has a rounded tip. In ventral view, the choanal processes come completely around to be underlapped by the ventral surface of the palatine. The ventromedial termination of the choanal process is diamond-shaped and has a spatulate posterior projection (Fig. 7A). The choanal process easily can be mistaken for the shorter palatine process of the vomer, although the latter terminates at about the midpoint of the body of the palatine in ventral view.

#### *Rhinophis drummondhayi*

The anterior vomerine processes of the ventral surface of the palatines have a distinct triangular morphology, whereas in the other taxa examined the tips are rounded. The lateral process is robust, slightly hooked, and directed anterolaterally. The overlying loop is closed, and no suture is visible. Internally, the ventromedial terminus of the choanal process is diamond-shaped, like that of *R. blythii*. In lateral view, the tapering of the pterygoid process occurs anterior to the frontal-parietal suture and at the optic foramen. The anterior half of the palatine extends laterally to the edge of the maxillary process of the prefrontal and maintains contact with the maxilla from that point posteriorly until the initiation of tapering of the pterygoid process. The groove in the pterygoid process is much longer than in any other specimen examined, covering greater than half the length of the process (Figs. 7B; 18B). The pterygoid process has a small medial flange as in *R. blythii*, but this flange is not as well-developed. Excluding the CN V_2_ opening, only a single foramen occurs in the lateral surface of the palatine.

#### *Rhinophis philippinus*

The choanal process of the palatine resembles that of *R. blythii* and *R. drummondhayi*, except at the element's posterior tip, which is fluted rather than diamond-shaped (Fig. 7C). In lateral view the anterior portion of the palatine extends laterally to the terminus of the maxillary process of the prefrontal, but has only a short contact with the maxilla, farther posteriorly. The base of the pterygoid process is more gradually tapered in lateral view than in the other taxa examined, being J- rather than L- shaped. The origination of the pterygoid process occurs between the optic foramen and the frontal-parietal suture. The groove for the reception of the pterygoid is wide and long (Fig. 19A), although not as elongate as in *R. drummondhayi*. In TMM M-10038 no tiny, lateral foramen is visible, whereas in TMM M-10037 the foramen is clearly present. In both, the lateral process of the palatine is small and obscured by the maxilla, but is clearly the ventral surface of a closed loop.

#### *Rhinophis homolepis*

The choanal process of the palatine is as observed in *R. blythii* and *R. drummondhayi*. In ventral view, the lateral process is thin, straight, and projects anterolaterally. The overlying loop is complete, although a suture is visible. In lateral view, the anterior half of the palatine extends laterally to the edge of the maxillary process of the prefrontal and then contacts the maxilla for a short distance posteriorly, until the origination of the pterygoid process. The base of the pterygoid process is located posterior to the frontal-parietal suture. No tiny foramen is visible on the lateral surface of the palatine.

#### *Brachyophidium rhodogaster*

In ventral view, the palatine has a rounded anterior vomerine process as in *U. woodmasoni* and most other taxa we examined (Fig. 21G). The lateral process, which is again the ventral surface of a closed loop, has a hooked morphology. The loop extends farther laterally than in the examined species of *Rhinophis* and *Uropeltis*, and this structure has a noticeably angled corner at its widest point (Fig. 21G,H). Unlike in *U. woodmasoni* the loop does not form right angles with the ventral floor and the lateral surface of the palatine.

In dorsolateral view, the anterior half of the palatine reaches the lateral extent of the maxillary process of the prefrontal, and posteriorly has a short contact with the maxilla before the tapering of the pterygoid process begins. The posterior tapering for that process originates anterior to both the frontal-parietal suture and the optic foramen and is gradual, producing a margin similar to that observed in *R. philippinus*. The groove for the reception of the pterygoid is proportionately wider than in *U. woodmasoni* (Figs. 20,21H). In the majority of specimens of *B. rhodogaster* a single lateral foramen occurs just below the suture with the frontal, although the foramen is markedly smaller in diameter than in the species of *Rhinophis* and *Uropeltis* examined. In one specimen (TMM M-10022) two foramina are present and in two others (TMM M-10014, -10018) no foramina were visible.

### Pterygoid

The pterygoid is a fairly straight, dorsoventrally compressed bone that primarily is aligned anteroposteriorly when in articulation. It underlaps the ectopterygoid along the anterolaterally-placed and slightly tapered ectopterygoid process. That process has a shallow groove for the ectopterygoid etched into its dorsal surface; the groove continues posteriorly a short distance onto the body of the pterygoid. An anteromedially directed palatine process forms the anterior end of the element. That process underlaps the palatine by inserting into a groove on the posteroventral surface of the pterygoid process of the palatine. Posterior to the junction of the ectopterygoid and palatine processes, the pterygoid closely approaches the ventrolateral surface of the braincase. The remaining posterior extent of the pterygoid follows the margin of the braincase, but much unossified tissue intervenes between the bones. The posterior tip ( = quadrate ramus of [17]) widens to form a broadly pointed, mediolaterally compressed process that is sandwiched between the ventrolateral surface of the braincase and the medial surfaces of the articulating quadrate and compound bones. The quadrate ramus is tilted medially to follow the curvature of the braincase. The pterygoid is edentulous in all uropeltids [17].

#### *Uropeltis woodmasoni*

**Figure 22:**
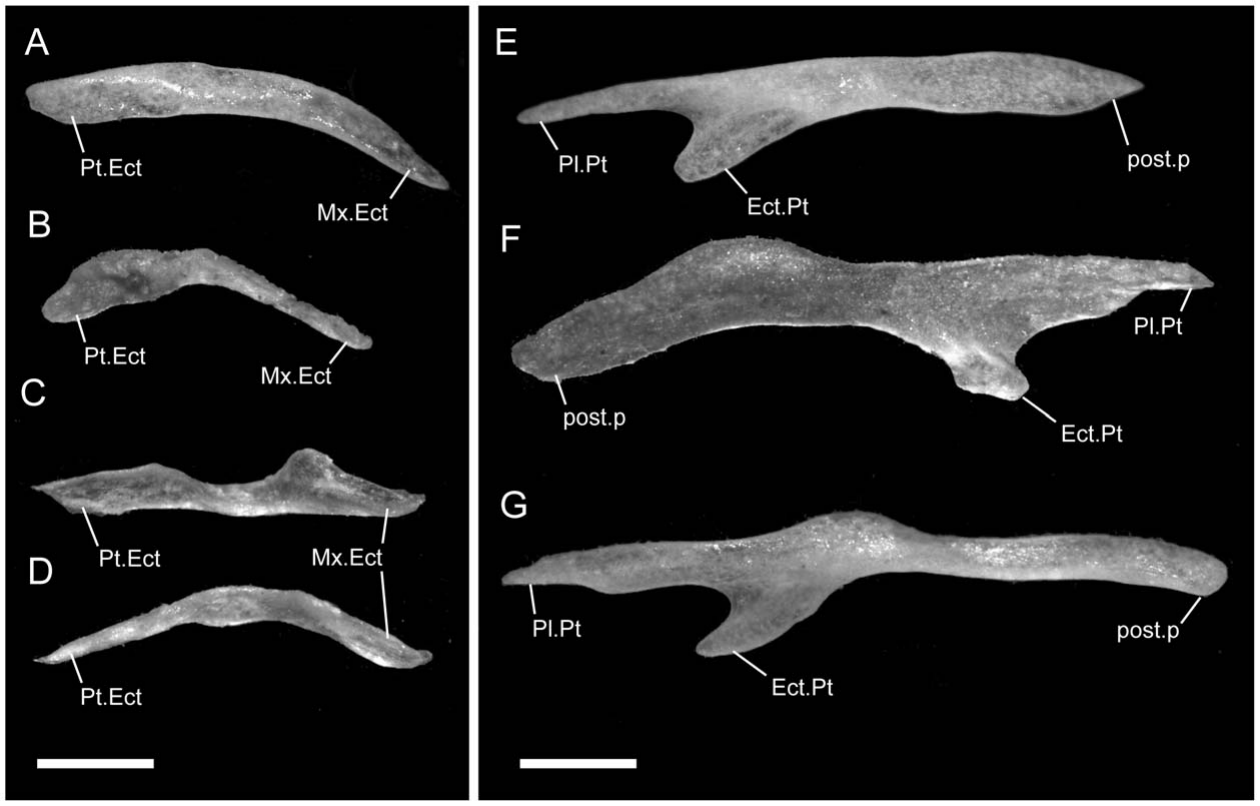
Disarticulated ectopterygoids and pterygoids. Anterior is to the right unless noted; scale bars = 0.5 mm. A,E from *U. woodmasoni* (TMM M-10001); B,F from *U. melanogaster* (TMM M-10045); C,D from *B. rhodogaster* (TMM M-10016); and G from *B. rhodogaster* (TMM M-10022). Right ectopterygoids in ventral (A–C) and dorsal (D) views; left pterygoids (E,G; anterior to the left) and right pterygoid (F) in dorsal views. Ect.Pt = ectopterygoid process of pterygoid; Mx.Ect = maxillary process of ectopterygoid; Pl.Pt = palatine process of pterygoid; post.p = posterior process of pterygoid; Pt.Ect = pterygoid process of ectopterygoid.

In *U. woodmasoni*, the ectopterygoid process is dorsoventrally compressed anteriorly. The angle formed at the junction of the ectopterygoid and palatine processes is approximately 45°, and the ectopterygoid process is usually between one-quarter and one-half the length of the palatine process (Fig. 22E). In ventral view, about three-quarters of the way from anterior to posterior, the pterygoid curves and bends laterally toward the otic region. In lateral view the element shows a dorsally convex arch, sloping upward from the lowest position at the posterior end to the highest point at the anterior end. The posterior tip of the pterygoid is spatulate in *U. woodmasoni*.

#### *Uropeltis rubromaculata*

The ectopterygoid process of the pterygoid is broader and more rounded than that of *U. woodmasoni*, and reaches half the length and twice the width of the associated palatine process. The angle between the two is greater than 45°.

#### *Uropeltis melanogaster*

The pterygoid has a higher arch in lateral view and more curvature in dorsal view than in *U. woodmasoni*. The bend originates earlier, approximately halfway along the bone from anterior to posterior. A broad, short flange or extension of the apex of curvature occurs posterior to the center of the pterygoid (Fig. 22F). The ectopterygoid process is short, approximately one-quarter the length of the palatine process. The anteriormost tip of the palatine process is irregular. The palatine process is wide at its base, but gradually tapers anteriorly. The process becomes dorsoventrally compressed on its lateral side near its base, giving the impression of a thin sheet (i.e., web) of bone between the palatine and ectopterygoid processes. At about three-quarters of the distance anteriorly, the tapering becomes abrupt and the resulting tip is pointed and slender. The posterior process is rounded.

#### *Rhinophis blythii*

The pterygoid is robust and smooth and has a cylindrical ectopterygoid process with a more rounded tip (Fig. 7A). The palatine process is also much more robust than in any other species of *Uropeltis* or *Rhinophis* we surveyed. As in *U. woodmasoni*, curvature toward the otic region is smooth and lacks a pronounced bend.

#### *Rhinophis drummondhayi*

The ectopterygoid process is narrow, and the palatine process is slender (Fig. 7B). The ectopterygoid process is less than a quarter of the length of the palatine process. The angle between the processes is slightly less than 45°, and their junction occurs farther anteriorly than in *Uropeltis*, approximately one-quarter of the way from the anterior end of the bone. A distinct bend with a roughly square flange or extension is placed at the point where the posterior tip curves upward toward the otic region. This occurs three-quarters of the way down the bone, moving from anterior to posterior.

#### *Rhinophis philippinus*

The pterygoid is relatively straight, although a slight curve occurs near its center. The ectopterygoid process is approximately one-quarter the length of the palatine process and is cylindrical and more rounded, but has the same width as the base of the latter (Fig. 7C). The palatine process gradually tapers anteriorly to a narrow, rounded tip and is rough and irregular. The posterior tip of the pterygoid tapers slightly to end in a blunt, rounded point.

#### *Rhinophis homolepis*

The single examined skull of *R. homolepis* is small, and the pterygoid exhibits the same irregularity common to that of other small specimens. The ectopterygoid process is broad, blunt, and nearly square (Fig. 7D). It is one-third the length of the palatine process, but approximately twice as wide. The junction with the ectopterygoid process is at an angle slightly greater than 45°. Posterior to the junction, there is little curvature toward the otic region. Uniquely, the posterior half of the bone widens just posterior to the level of the vidian canal in the braincase before tapering to a triangular point posteriorly.

#### *Brachyophidium rhodogaster*

The ectopterygoid process is cylindrical in cross section, rather than more dorsoventrally compressed as in *U. woodmasoni*. The ectopterygoid process can be one-quarter to one-third the length of the palatine process. Both are smooth, robust, and narrow. The palatine process is relatively longer than that of *U. woodmasoni* and tapers abruptly only at the anteriormost tip for insertion into the pterygoid process of the palatine (Fig. 22G). In TMM M-10019 the tip is extremely pointed and jagged. The angle of the junction between the two processes is approximately 45°. Farther posteriorly, behind the junction and approximately midway along the bone, the pterygoid curves toward the otic region. A broad, shallow, and rounded medial extension occurs at that point (Fig. 22G). Just posterior to the curve, the pterygoid twists much more than in the other genera, creating a large tissue-filled gap between the anterior and posterior contact with the braincase. At this twist, the bone narrows and then widens posteriorly before finally tapering to a narrowly rounded tip. One specimen, TMM M-10013, differs from all other *B. rhodogaster* examined. The pterygoid on the right side terminates in a squared posterior tip, while that on the left has a crooked, blunt end. The ectopterygoid process is nearly half the length of the palatine process and is strongly hooked and pointed, tapering from the base to the anterior tip.

### Ectopterygoid

Uropeltids have a small ectopterygoid that extends from the lateral process of the pterygoid to the posterior tip of the maxilla as a laterally compressed bar. The anterior tip is slender and pointed and has a roughened, lateral surface for contact with the maxilla. The posterior tip is wider than the anterior one and also is dorsoventrally compressed. On the ventral surface of the posterior portion is a medially angled groove for articulation with the pterygoid.

#### *Uropeltis woodmasoni*

A smooth, obtuse bend (∼160°) at the midpoint of the ectopterygoid gives it a curved appearance (Fig. 22A). Because the contact with the maxilla is longer than that with the pterygoid, the curvature occurs at the posterior extent of the maxillary articulation (Fig. 5A). In dorsal view, the apex of the curve is directed laterally. The posterior tip is less dorsoventrally compressed than in other taxa.

#### *Uropeltis rubromaculata*

The ectopterygoid is more arched than that of *U. woodmasoni* in lateral view. The curvature of the bone is also more prominent, forming a more acute and abrupt angle. The apex is slightly expanded laterally to form a small triangular flange (Fig. 5B).

#### *Uropeltis melanogaster*

The ectopterygoid appears irregular and weakly developed compared to the other *Uropeltis* taxa (Fig. 22B). The curvature of the bone forms a sharper (though still obtuse) angle, but does not form a lateral flange as in *U. rubromaculata*. The posterior half of the ectopterygoid is broader horizontally and more dorsoventrally compressed than the anterior half.

#### *Rhinophis blythii*

The ectopterygoid strongly resembles that of *U. woodmasoni*, but possesses a larger surface area for contact with the maxilla (the maxilla covers more than half the length of the ectopterygoid; Fig. 6A) and is also less strongly arched.

#### *Rhinophis drummondhayi*

The ectopterygoid shows less curvature than that of *U. woodmasoni*. A slight, dorsally convex arch characterizes the bone in lateral view. A subtle apex, formed by the transition between the laterally compressed anterior half and the dorsoventrally compressed posterior half of the element, occurs immediately posterior to the contact with the maxilla.

#### *Rhinophis philippinus*

The ectopterygoid is straighter than that of *U. woodmasoni* and *R. homolepis*, but has a slight bend just posterior to the contact with the maxilla. Little of the bone is free of contact with either the pterygoid or the maxilla, and that portion is only slightly arched in lateral view.

#### *Rhinophis homolepis*

The ectopterygoid is similar to that of *U. woodmasoni*, although the element is slender and somewhat irregular, as in *U. melanogaster*. However, in dorsal view, the posterior half is not as wide as in *U. melanogaster*.

#### *Brachyophidium rhodogaster*

The ectopterygoid is arched in lateral view (Fig. 22D). The bone is straight in dorsal view and although it has subtle, irregular undulations, it lacks a consistent curvature among the individuals examined. The medial margin is smooth and all undulations are visible only along the lateral edge. The angular apex of one undulation occurs just posterior to the contact with the maxilla, and at that point the ectopterygoid abruptly narrows and twists into a horizontal position that is maintained along the posterior half of the bone (Fig. 22C). In some specimens the apex of that undulation is extended into a pointed, triangular flange (Fig. 5C). The ectopterygoid widens again posteriorly at the beginning of the contact with the pterygoid, but the former narrows once more as it approaches the parietal. The ectopterygoid overlaps the entire ectopterygoid process of the pterygoid, extending past the fork in the latter and nearly contacting the parietal. The contact between ectopterygoid and maxilla is nearly horizontal but is angled medially. The groove for the pterygoid is much shallower than the groove for the maxilla. The ventral margin of the latter groove is folded over medially to form a small shelf, ventral to which the ectopterygoid process of the maxilla fits.

### Braincase (Spheno-Otooccipital Complex)

All uropeltids exhibit a high degree fusion in the posterior braincase, and all lack a separate supratemporal bone. As reported previously, in the two species of *Melanophidium* the opisthotic and exoccipital are fused together to form the otooccipital that is characteristic of nearly all snakes, but the exoccipital and basioccipital are joined seamlessly also [17]. In *Plectrurus*, *Pseudotyphlops*, *Rhinophis*, *Brachyophidium*, and *Uropeltis*, fusion is carried to an extreme, and all braincase elements fuse to form a single element in the adult. In all specimens the fused braincase complex appears to comprise the supraoccipital, otooccipitals, basioccipital, exoccipitals, prootics, laterosphenoid, basisphenoid, and parasphenoid, but that inference requires testing through developmental data. No sutures are visible, although the margins of the prootic and supraoccipital regions are delimited by the position of the semi-circular canals, which are visible through the thin bone.

#### *Uropeltis woodmasoni*

**Figure 23:**
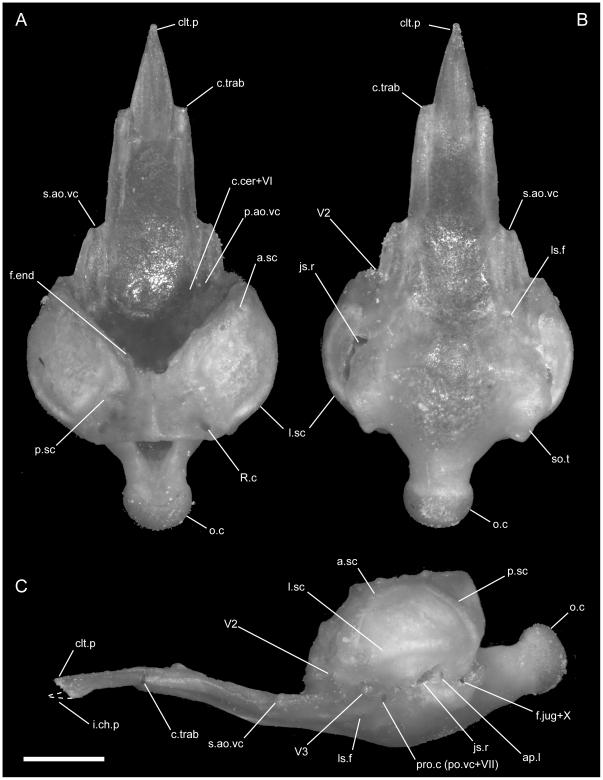
Disarticulated otooccipital complex of *Uropeltis woodmasoni* (TMM M-10021). Anterior up unless noted; scale bar = 1.0 mm. (A) Dorsal, (B) ventral, and (C) lateral (anterior to the left) views, note broken i.ch.p. a.sc = anterior semi-circular canal; ap.l = apertura lateralis recessus scalae tympani; c.cer = cerebral carotid foramen; c.trab = ossified crista trabecularis; clt.p = cultriform process; f.end = endolymphatic foramen; f.jug = jugular foramen; i.ch.p = interchoanal process of sphenoid; js.r = juxtastapedial recess; l.sc = lateral semi-circular canal; ls.f = laterosphenoid foramen; o.c = occipital condyle; p.ao.vc = primary anterior opening of vidian canal; p.sc = posterior semi-circular canal; po.vc = posterior opening of vidian canal; pro.c = prootic canal; R.c = Rieppel's canal; s.ao.vc = secondary anterior opening of vidian canal; so.t = spheno-occipital tubercle?; V2 = foramen for maxillary branch of trigeminal nerve; V3 foramen for mandibular branch of trigeminal nerve; VI = foramen for abducens nerve; VII = foramen for facial nerve seven; X = vagus nerve.

Endocasts of the associated soft tissues of the CT-scanned specimen, TMM M-10006, were described in a previous publication [51]. In dorsal view of the disarticulated braincase complex, the anterior end of the ossified crista trabecularis is located along the lateral margin of the sphenoid region (Fig. 23A). In TMM M-10001, a groove along the dorsal surface of the crista trabecularis ends posteriorly at a small foramen that enters a narrow canal in the floor of the braincase. The canal (visible within the thin bone) parallels the lateral margins of the sphenoid region and opens posteriorly as a minute foramen anteromedial to the secondary anterior opening of the vidian canal. The narrow canal was not reported previously in any uropeltids or related taxa and is closed anteriorly on both sides of the other disarticulated specimen, TMM M-10021. On the left side of that individual, a minute foramen opens anteromedial to the secondary anterior opening of the vidian canal, but the foramen is absent on the right side. The structure in TMM M-10021 suggests that the narrow canal is a byproduct of fusion or ossification during development and does not transmit nerves or vessels. Farther posteriorly, the proportionately large size and laterally inflated shape of the otic capsules is evident. The sagittal crest, which begins anteriorly on the parietal, continues to the posterior margin of the supraoccipital region.

In the posterolateral portion of the supraoccipital region, a posteroventrally directed canal pierces the dorsal surface of the bone medial to the semi-circular canals, on both sides of the skull. Those canals open posteriorly on either side of the foramen magnum, posterior to the inferred boundary between the fused prootic and exoccipital (Figs. 5,6; [55]: fig. 2.29). The openings were figured by previous authors but were not described [17], [55]. They recently were named Rieppel's canals [19], but their function is unknown. Careful dissection or histological sectioning of a specimen with soft tissue is required to ascertain what tissues (if any) pass through the canals. In at least some specimens of uropeltids the openings appear to be incompletely formed by a pinching of the posterior margin, suggesting that they are structural by-products of fusion (see [17], fig. 5C, *Pseudotyphlops philippinus*; [55], fig. 2.29B, *Uropeltis ocellata*). In a single specimen of *U. woodmasoni* (TMM M-10005) the canal was incomplete on the right side only.

The occipital condyle of *U. woodmasoni* has a long, robust neck (Figs. 5A; 23). Posterior to the trough leading down into the braincase, at the narrowest point of the neck, the dorsal surface of the occipital condyle has a shallow depression or fovea for the continuation of the brainstem [17]. This is also visible in the other species of *Uropeltis* and in *Rhinophis* (Figs. 5A,B;6).

In posterior view, dorsolateral to the occipital condyle and ventral to the posterior opening of the Rieppel's canal on either side, is a single hypoglossal (cranial nerve [CN] XII) foramen. The hypoglossal foramina are located within the inferred exoccipital region, level with the dorsal surface of the occipital condyle, and medial to Rieppel's canals. Viewed through the foramen magnum, an additional small foramen [34] is visible in each medial wall of the otooccipital region. That tiny foramen was named a dorsal metotic foramen by Rieppel [34], and should not be confused with the more anteriorly-positioned endolymphatic foramen. The function of the blood vessel transmitted by the dorsal metotic foramen is unknown [34]. The foramen is positioned at approximately the dorsal extent of the embryonic metotic fissure between the otic capsule and the occipital arch, and that position was the basis for the name. (O. Rieppel, pers. com., Oct. 2011). The expression of the embryonic metotic fissure in adult squamates typically is restricted to the recessus scala tympani and the foramen for the vagus nerve in the ventral portion of the braincase, but we retain an anglicized rendering of Rieppel's original terminology here for clarity.

In ventral view, at the posterior end of the basioccipital region, two rounded enlargements are visible, and these may either be homologous with the sphenooccipital tubercles of other squamates or by-products of complete fusion (Fig. 23B). These structures ascend to form part of the lower margin of the crista circumfenestralis, which partially encloses the juxtastapedial recess in lateral view (Fig. 23C). Anteromedial to the enlargements, at the level of the articulation of the pterygoid and lower jaw, a shallow, rounded depression occurs on either side of the posterior end of the basisphenoid region. The anterior and posterior rims of the depressions are inflated or thickened with calcified cartilage. Anteriorly, the sphenoid region tapers to form a pointed interchoanal process that comes to rest between the choanal processes of the palatines. In between the base of the pterygoid process of the palatine and the clasp between the pterygoid and palatine, at the ventrolateral margin of the sphenoid, the anterior end of the ossified crista trabecularis is present. A groove for the cartilaginous portion of the crista continues anteriorly (Fig. 9B).

**Figure 24:**
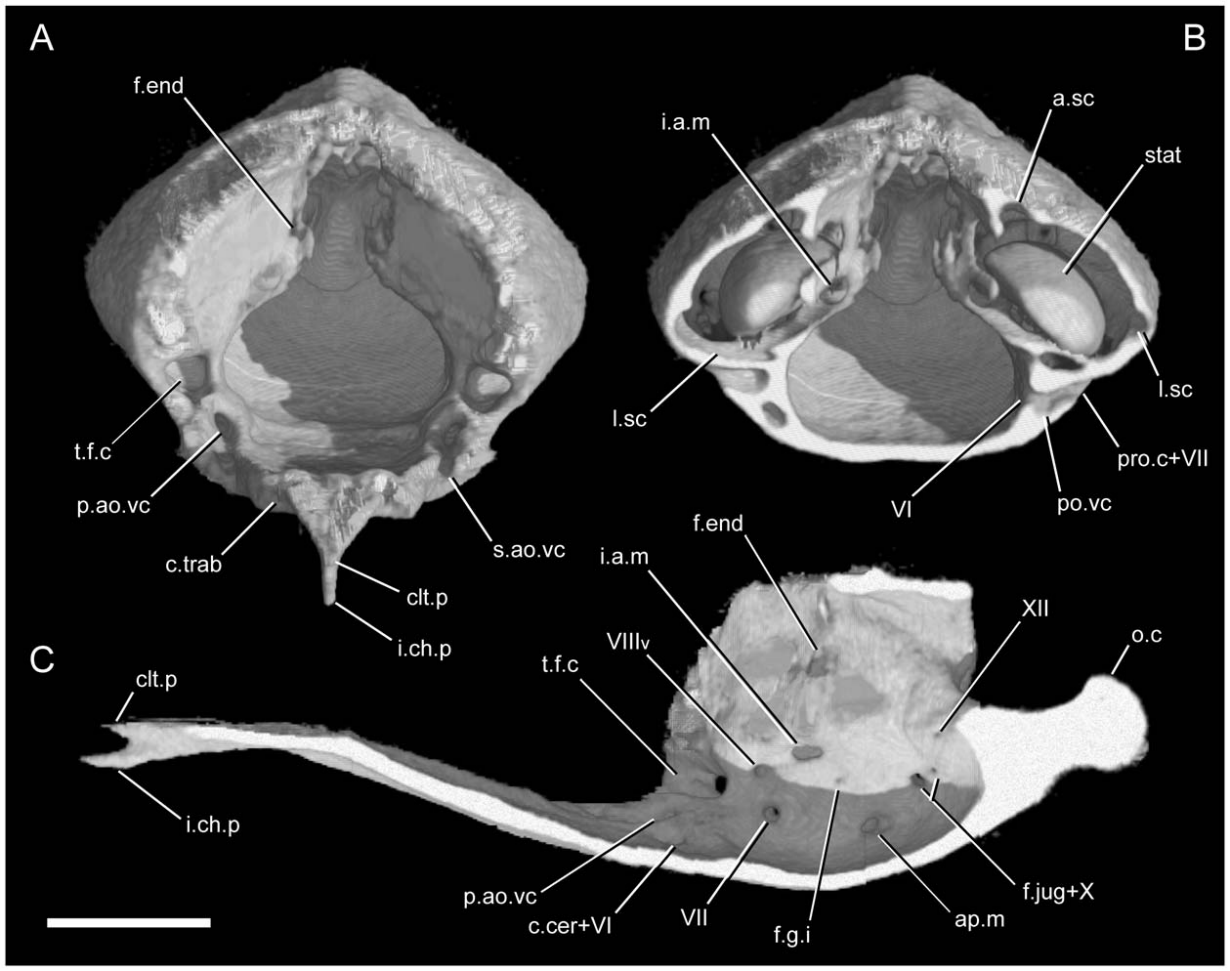
CT images of the otooccipital complex of *Uropeltis woodmasoni* (TMM M-10006). Scale bar = 1.0 mm. (A) Anterior view; (B) Anterior cut-away at level of otic capsule; (C) Lateral view, anterior to left. a.sc = anterior semi-circular canal; ap.m = apertura medialis recessus scalae tympani; c.cer = cerebral carotid foramen; c.trab = ossified crista trabecularis; clt.p = cultriform process; f.end = endolymphatic foramen; f.jug = jugular foramen; i.a.m = internal auditory meatus; i.ch.p = interchoanal process of sphenoid; l.sc = lateral semi-circular canal; o.c = occipital condyle; p.ao.vc = primary anterior opening of vidian canal; po.vc = posterior opening of vidian canal; pro.c = prootic canal; s.ao.vc = secondary anterior opening of vidian canal; s.cir = semi-circular canal; stat = statolithic mass; t.f.c = trigeminal facialis chamber (for trigeminal branches two and three); VI = abducens nerve; VII = facial nerve seven (to prootic opening); VIIIv = vestibular branch of auditory nerve; X = vagus nerve; XII = hypoglossal nerve.

In lateral view, an interchoanal process extends anteroventrally from the anterior tip of the parasphenoid as a prominent triangular keel (Figs. 23C; 24C). This ‘keel’ was also reported in *Plectrurus aureus*, but was described incorrectly as separate from the interchoanal process [19]. The morphology of the process in *U. woodmasoni* and *P. aureus* [19] is sharply triangular. In *U. woodmasoni*, the interchoanal process (i.e., keel) extends anteriorly beyond the anterior tip ( = cultriform process [32]) of the sphenoid. Overall, the sphenoid region slopes dorsally from the fusion with the prootic, to the end of the ossified crista trabecularis, anteriorly (Fig. 23C). Beyond this point, the anterior tip of the sphenoid slopes ventrally. At the anterior margin of the prootic region, the external opening for CN V_2_ is visible as a notch. Posterior to the CN V_2_ foramen is the opening for the mandibular branch of the trigeminal nerve (CN V_3_), and in between and ventral to those is a tiny laterosphenoid foramen, located within the relatively broad laterosphenoid region. TMM M-10001 possesses a second small foramen ventral to the laterosphenoid, but this is not present in any other observed *U. woodmasoni*. Posteroventral to Cn V_3_ opening, immediately anterior to the juxtastapedial recess, the prootic canal is visible. Internally the canal is divided. The larger, anterior division is the posterior opening of the vidian canal; the smaller division is the external opening for the facial nerve (CN VII) [17].

**Figure 25:**
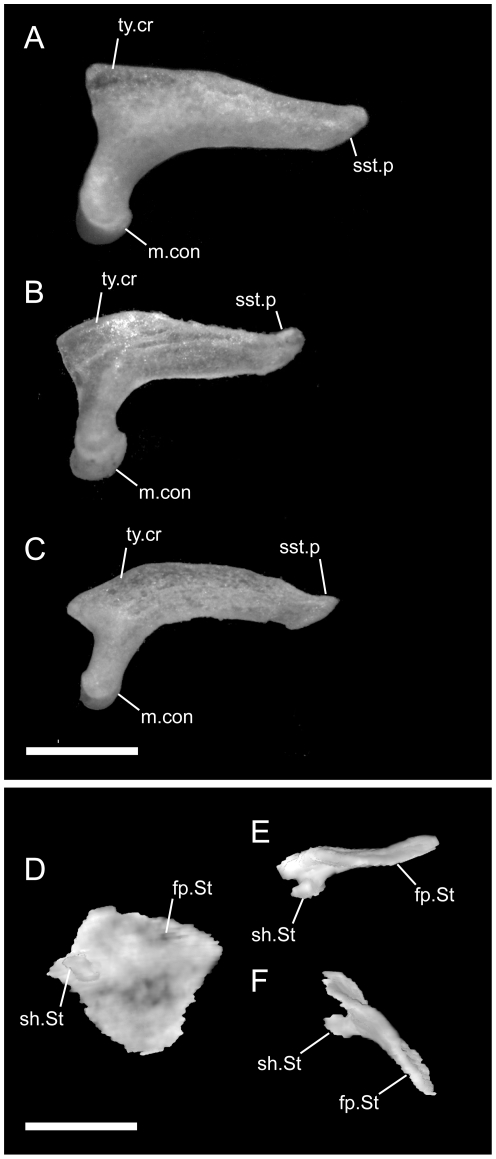
Disarticulated quadrates and stapes. Anterior is to the left unless noted; scale bars = 0.5 mm. A from *U. woodmasoni* (TMM M-10001); B from *U. melanogaster* (TMM M-10045); C from *B. rhodogaster* (TMM M-10027); and D–F from CT scans of *U. woodmasoni* (TMM M-10006). Left quadrates in lateral view (A–C), and right stapes in ventrolateral (K, anterior to the right), dorsal (L, anterior to the right), and anterolateral (M, lateral to the left) views. fp.St = stapedial footplate; m.con = mandibular condyle of quadrate; sh.St = stapedial shaft; sst.p = suprastapedial process (caudal process) of quadrate; ty.cr = tympanic crest of quadrate.

**Figure 26:**
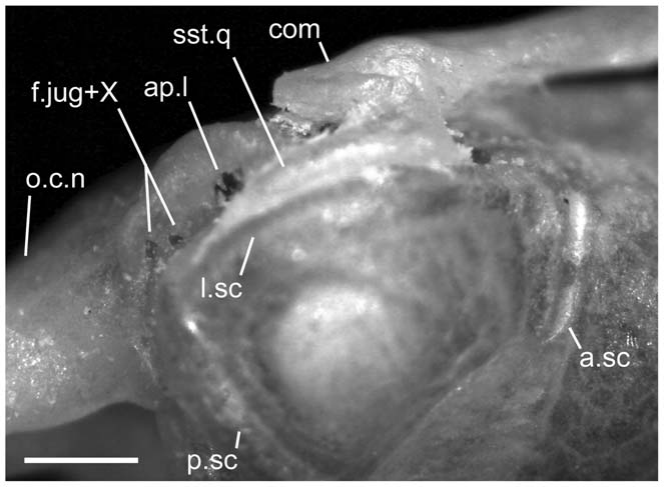
Magnified view of the posterior end of the crista circumfenestralis of *Uropeltis woodmasoni* (TMM M-10008) in dorsolateral view. Anterior is to the right; scale bar = 0.5 mm. This specimen has a double opening for the passage of cranial nerve X, the jugular vein, and associated tissue. a.sc = anterior semi-circular canal; ap.l = apertura lateralis recessus scalae tympani; com = compound bone; f.jug = jugular foramen; l.sc = lateral semi-circular canal; o.c.n = neck of occipital condyle; p.sc = posterior semi-circular canal; sst.q = suprastapedial process of the quadrate; X = foramen for vagus nerve.

The lateral exposure of the juxtastapedial recess varies gradationally across the specimens from wide open laterally to somewhat restricted at the anterior half. The large stapedial footplate, with a short and narrow stapedial shaft (Fig. 25D–F), takes up most of the space in the recess. The edges of the stapedial footplate are thin and damaged easily; they do not reconstruct clearly in CT scans because of the lower limits of the resolution of standard CT data sets (note scale bar in Figure 25D–F). In dried skulls the stapes sits loosely within the juxtastapedial recess, and in most specimens the stapes is either displaced or has fallen out and been lost. Within the juxtastapedial recess the lateral aperture of the recessus scalae tympani, foramen pseudorotundum, and the jugular foramen, which also carries the vagus nerve (CN X) [17], are visible from roughly anterior to posterior. The posteriormost opening is located in a cup-like recess that is formed by a posterior extension of the lower margin of the crista circumfenestralis, but is separated from the juxtastapedial recess by a low wall of bone (Fig. 26). Posteriorly, a short, shallow groove originates at this cup and wraps around to the back of the skull. In TMM M-10002, on the left side, the ventral margin of that groove extends dorsally to form a small canal. Additionally, in TMM M-10001, the opening for CN X and the jugular vein is divided on both sides, whereas in TMM M-10008 the opening is divided on the right side, but not the left. In TMM M-10007, on the right side, the posterior margin of the cup pinches in to form a pseudo-division. In TMM M-10006 [51, this study] and TMM M-10001 the divisions are asymmetrical in size and are located deeply, but in TMM M-10008 the split is at the surface (Fig. 26).

In the anterior view of disarticulated skulls, the otic capsules are large and nearly make contact under the skull roof (Fig. 24A,B). The anteromedial face of each capsule contains a vertical endolymphatic foramen that is open dorsally (Fig. 23A). Anteroventral to the otic capsule is the trigemino-facialis chamber where the trigeminal nerve is inferred to split into the separate V_2_ and V_3_ branches. At the posterodorsal corner of the chamber, a tiny foramen links the chamber to the otic capsule. Ventral to the chamber is the primary anterior opening for the vidian canal, and anteroventral to that the secondary anterior opening for the vidian canal is also visible. At the most posterior internal surface of the skull, in both of the completely disarticulated specimens of *U. woodmasoni* (TMM M-10021, -10001), tiny foramina were observed entering the occipital condyle, on either side of the midline. Narrow canals between the exoccipital and basioccipital regions are visible through the thin bone, and appear to connect the foramina to the internal opening for CN X and the jugular vein on either side of the braincase. Those condylar foramina also are visible in the CT scans of TMM M-10006.

In the sagittal sections of CT scans (and oblique views of disarticulated skulls), beginning anteriorly at the junction of the sphenoid region with the prootic region, a series of three openings from ventromedial to dorsolateral includes a passage for the cerebral carotid artery, the primary anterior opening of the vidian canal, and the large trigeminal-facialis chamber through which can be seen the notch for CN V_2_ anteriorly and the opening for CN V_3_ posteriorly (Fig. 24C). In TMM M-10021, on the right side only, a small opening links the trigeminal-facialis chamber to the internal passage of the vidian canal. In *U. woodmasoni*, internally, the opening for the cerebral carotid artery (which may also carry a branch of the abducens nerve (CN VI) [34]) joins with the vidian canal before both enter the prootic canal by means of a single passage. Additionally, a tiny foramen of unknown function pierces the lateral wall of the groove connecting the primary and secondary anterior openings of the vidian canal. Posterior to the trigemino-facialis chamber are two additional openings, one dorsal, which leads into the otic capsule and transmits the vestibular branch of the auditory nerve (CN VIIIv; see [51]), and a ventral one that leads directly into the prootic canal and transmits nerve CN VII [17], [34], [51]. Posterior to those and entering the floor of the otic capsule is the internal auditory meatus, which transmits the cochlear branch of the auditory nerve (CN VIIIc). Posteroventral to that opening is a small foramen that may be the internal glossopharyngeal foramen. In CT slices of TMM M-10006, that opening appears to lead to the recess medial to the foramen pseudorotundum, but it is difficult to trace (see also [51]). A larger opening located posteroventrally is the medial aperture of the recessus scalae tympani. At the back of the braincase, there is a large opening for the jugular vein and vagus nerve (as well as an opening for its much smaller division, when the opening is divided). Dorsomedial to these, at the back of the braincase, is a single, small opening for the hypoglossal nerve (CN XII), on either side of the foramen magnum (Fig. 24B).

#### *Uropeltis rubromaculata*

The braincase is similar to that of *U. woodmasoni*. However, some differences are present, including lack of a dorsal metotic foramen and lack of paired, ventral sphenoid depressions. In addition, the prootic canal and the opening for V_3_ are separated only by a thin wall of bone, and a small, round fovea occurs ventrally along the midline, at the level of the articulation with the lower jaw. The hypoglossal openings are single, but the opening for CN X and the jugular vein is divided on the left side only. In ventral view the interchoanal process (keel) appears to be present, but without disarticulated specimens its presence cannot be confirmed.

#### *Uropeltis melanogaster*

In the two disarticulated specimens the ventral interchoanal process is prominent but does not extend anteriorly past the anterior tip ( = cultriform process [32]) of the parasphenoid. The openings for CN V_2_ and CN V_3_ are at the same relative positions as in *U. woodmasoni*, but they are closer together, reducing the width of the laterosphenoid region. In TMM M-10045 the laterosphenoid foramen is located below the CN V_2_ opening, although in TMM M-10032 it is between the CN V_2_ and CN V_3_ openings, as in *U. woodmasoni*. Additionally, on the left side of TMM M-10032, a tiny foramen occurs immediately posterodorsal to the CN V_2_ notch; from the structure on the opposite side it is inferred that whatever is transmitted by that tiny foramen usually is incorporated into the opening for CN V_2_. The prootic canal and the CN V_3_ opening are so close together that they form one opening with a division located just below the level of the external surface. Only one foramen with a deep internal division occurs on the left side of TMM M-10045. The opening for CN X and the jugular vein is single, and there is a tiny, narrow shelf immediately dorsal to the foramen. Inside the braincase, no groove was observed along the dorsum of the crista trabecularis and the specimens lack foramina entering the occipital condyle, as well as dorsal metotic foramina.

#### *Rhinophis blythii*

The openings for CN V_2_ and CN V_3_ are in the same positions as in *U. woodmasoni*, although the prootic canal is shifted dorsally and appears to be merged with the juxtastapedial recess. The canal is separated from the opening of the V_3_ by a narrow partition of bone. On the left side the laterosphenoid foramen is ventral to and between the openings for CN V_2_ and CN V_3_, but on the right it is located posterodorsally, adjacent to the anteroventral margin of the CN V_3_ foramen. A tiny dorsal metotic foramen is visible when viewed through the foramen magnum, and the opening for CN X and the jugular vein is single. The opening for the hypoglossal nerve also is single. The interchoanal process is visible, but because the specimen is articulated, it is unknown if that process extends anteriorly beyond the cultriform process.

#### *Rhinophis drummondhayi*

The openings for CN V_2_ and CN V_3_ are positioned similarly to those in *U. woodmasoni*. The prootic canal is relatively small, is close to the juxtastapedial recess and the CN V_3_ foramen, and is separated from each of those by a thin wall of bone, remaining distinct. The laterosphenoid foramen is shifted posteriorly to lie ventral to the anterior margin of the opening for CN V_3_. An additional foramen is located dorsal to, and somewhat confluent with, the notch for CN V_2_. A small dorsal metotic foramen is present, as well as a single hypoglossal opening and a single opening for CN X and the jugular vein. Rieppel's canal is incomplete on both sides of the skull. Because the specimen is fully articulated, the presence of an interchoanal process could not be confirmed.

#### *Rhinophis philippinus*

The sagittal crest is weaker than in other examined species of *Uropeltis* and *Rhinophis*. In lateral view, the juxtastapedial recess is greatly restricted and has almost no open space anterior to the stapes. The openings for CN V_2_ and CN V_3_ are found in the same relative positions as in *U. woodmasoni*, but the prootic canal is much closer to the opening for CN V_3_ than to the juxtastapedial recess, and is distinctly separate from both. In TMM M-10037 the laterosphenoid foramen is located ventral to and between the openings for CN V_2_ and CN V_3_, but in TMM M-10038 the laterosphenoid foramen is directly ventral to the CN V_2_ foramen, and an additional foramen is centered between the laterosphenoid and the CN V_2_ foramen. In the latter specimen, two hypoglossal foramina occur on each side, whereas in the former they are single. Both specimens have a single opening for CN X and the jugular vein and both lack a dorsal metotic foramen.

TMM M-10038 is the only specimen of *Rhinophis* we examined that has a disarticulated braincase complex (disassociation occurred after preparation). Internally, the locations and connectivity of foramina follow that of *U. woodmasoni*, with a few small differences. As in *U. melanogaster* there is no groove dorsal to the crista trabecularis. On both sides of the braincase, similar to TMM M-10021 (*U. woodmasoni*) a tiny passage connects the vidian canal to the trigeminal facialis chamber. Additionally, at the back of the braincase, two tiny foramina, instead of a single opening, enter the occipital condyle on either side of the midline. Each foramen is connected to the opening for CN X and the jugular vein by a separate canal (visible through the bone). Internal hypoglossal openings could not be located definitively, and the small size of the external openings prohibited tracing their passage. In lateral view, TMM M-10038 has an interchoanal process ventral to the cultriform process of the sphenoid, and as in *U. woodmasoni*, the keel extends past the cultriform process of the sphenoid.

#### *Rhinophis homolepis*

The prootic canal has a robust division from the CN V_3_ opening, but only a narrow and deeply inset separation from the juxtastapedial recess, with which it appears to have merged. The opening for CN X and the jugular vein is single, as is the opening for the hypoglossal nerve. There is no dorsal metotic foramen, and the juxtastapedial recess is restricted, with the narrowest point occurring just posterior to its center. Because the specimen is fully articulated, the presence of an interchoanal process could not be confirmed.

#### *Brachyophidium rhodogaster*

**Figure 27:**
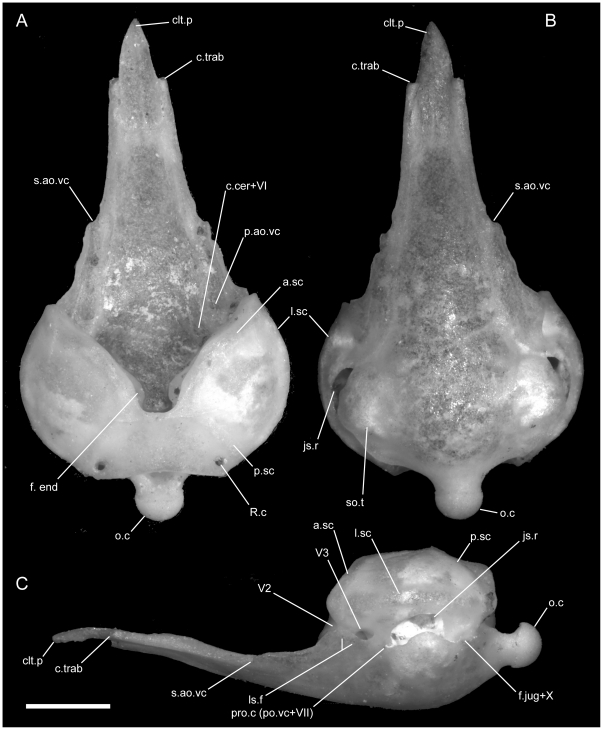
Otooccipital complex of *Brachyophidium rhodogaster* (TMM M-10023). Anterior up unless noted; scale bar = 1.0 mm. (A) Dorsal, (B) ventral, and (C) lateral (anterior to left) views. a.sc = anterior semi-circular canal; c.cer = cerebral carotid foramen; c.trab = ossified crista trabecularis; clt.p = cultriform process; f.end = endolymphatic foramen; f.jug = jugular foramen; js.r = juxtastapedial recess; l.sc = lateral semi-circular canal; ls.f = laterosphenoid foramen; o.c = occipital condyle; p.ao.vc = primary anterior opening of vidian canal; p.sc = posterior semi-circular canal; po.vc = posterior opening of vidian canal; pro.c = prootic canal; R.c = Rieppel's canal; s.ao.vc = secondary anterior opening of vidian canal; s.cir = semi-circular canal; so.t = spheno-occipital tubercle?; V2 = foramen for maxillary branch of trigeminal nerve; V3 foramen for mandibular branch of trigeminal nerve; VI = foramen for abducens nerve; VII = foramen for facial nerve seven; X = foramen for vagus nerve.

The basioccipital and basisphenoid are fused in all of our adult specimens, although previously those bones were reported to remain separate [17]. Thus, this species exhibits the same degree of fusion found in *Rhinophis* and *Uropeltis*. Dorsally, no sagittal crest occurs in the supraoccipital region. The canals piercing that area are more medially positioned than they are in species of *Uropeltis* and *Rhinophis* and open posteriorly inside the dorsal margin of the foramen magnum. The occipital condyle is short, lacking any solid neck between the triangular trough leading into the braincase and the actual condyle, and there is no posterior fovea as exhibited by *Uropeltis* and *Rhinophis* (Figs. 5C,27). The number of hypoglossal openings varies individually in *B. rhodogaster*. In TMM M-10019, TMM M-10027, and TMM M-10022 there are two on the right and one on the left, whereas in TMM M-10016, TMM M-10023, and TMM M-10020 there are two on the left and one on the right. In TMM M-10013, TMM M-10017, TMM M-10018, TMM M-10024, and TMM M-10014 the opening is single on both sides, and in TMM M-10015, TMM M-10026, and TMM M-10020 it is paired on both sides. When paired, one opening is located dorsal to the other, and the more dorsal opening is smaller. An exception occurs in specimen TMM M-10011, in which the opening is single on the right, but on the left it appears to be paired as a result of a deep division of one large foramen.

In ventral view, paired, small depressions are positioned in the basisphenoid region, each with a large amount of posterior thickening (not visible in the figures). Anterior to those, along the ventrolateral surface, are two large, shallow, crescentic depressions whose convex margins are directed medially. Unlike the condition exhibited by the other taxa, the anterior terminus of the crista trabecularis can be located posterior to the clasp between the pterygoid and palatine (refer to the section on the frontal for its relationship to the frontal-parietal suture). There is no keeled interchoanal process extending ventrally from the cultriform process of the sphenoid region (Fig. 27C).

In lateral view, the opening for CN V_2_ is proportionately smaller than in the species of *Uropeltis* and *Rhinophis* we examined, and the opening for CN V_3_ is more widely separated from it, creating a wider laterosphenoid region (Fig. 27C). The prootic canal is nearly confluent with the juxtastapedial recess, which is wide open laterally. The ossified portion of the stapedial shaft is even more reduced than in the other taxa examined. The laterosphenoid foramen is tiny and branches near the external surface. Among individuals of *B. rhodogaster* the foramen varies widely in its precise location, the location of the split, and the length of the branches. In the majority of the specimens, the bone is so thin that the split in the main branch is visible and the two openings can be identified, generally ventral to the V_3_ and V_2_ and at the same level with one another. Posterior to the juxtastapedial recess, and separated from it by a thin wall of bone, the opening for CN X and the jugular vein is single, except in TMM M-10026 and TMM M-10023, where the opening is bifurcated on the right. The opening is located in less of a cup-shaped recess than in our specimens of *Uropeltis* and *Rhinophis*, but it is still deeply recessed and is bounded laterally by a posteriorly extended lip of the crista circumfenestralis. Anterodorsal to the opening for CN X and the jugular vein, a small U-shaped shelf or groove opens anteriorly to accept the posterior tip of the caudal process of the quadrate. There is no obvious dorsal metotic foramen, although many specimens possess tiny pits that may have been open earlier in development.

Most *B. rhodogaster* exhibit a true opening (TMM M-10017, -10019, -10020, -10022–10024) or a narrow pit (TMM M-10014–10016, -10018) in the dorsum of the otic capsule inside the braincase, and that condition can differ between left and right in an individual. Internally along the sphenoid region, as in *U. melanogaster*, there is no groove and associated foramen located along the dorsal surface of the crista trabecularis. The overall pattern of cranial fenestration follows that of *Uropeltis*. However, in lateral view, inside the prootic canal, a third, anteroposterior opening leads to a small opening in the posterior wall of the medial opening of CN V_3_. In addition, a foramen is located inside the braincase between the hypoglossal nerve foramina and the opening for CN X and the jugular vein that is not found in our *Uropeltis* specimens. An unsuccessful attempt was made to trace this passage. TMM M-10026 was anomalous in lacking a branching laterosphenoid foramen and the two additional openings found in other *B. rhodogaster* but not in *Uropeltis*.

In specimens with two distinct extracranial hypoglossal openings on each side, two matching internal openings can be traced definitively to the two corresponding extracranial openings. In individuals with a single external opening, there is often a tiny or nearly closed second hypoglossal foramen internally. In disarticulated specimens, one or two blind openings penetrate the occipital condyle at the back of the braincase.

### Quadrate

The uropeltid quadrate differs notably from that of other snakes. Its most distinctive feature is an elongate, posteriorly directed caudal process ( = suprastapedial process of [17]). A similar process occurs in *Anomochilus* [49], but the nature of the articulation with the cranium is unique to uropeltids. Uropeltids lack a supratemporal bone (see [55] for discussion), and thus the quadrate articulates directly with the lateral surface of the braincase. In addition, the suspension of the quadrate occurs farther anteroventrally than it does even in *Anomochilus*.

#### *Uropeltis woodmasoni*

In lateral view, the quadrate is ventral to the midpoint of the height of the skull (Fig. 2A). The quadrate articulates with a groove formed by the inflated, dorsal margin of the crista circumfenestralis and is suspended just dorsal to the juxtastapedial recess, thus obscuring the latter from view. The quadrate wraps around the braincase posteriorly, and the caudal process maintains contact with the braincase along its full length. The mediolaterally compressed caudal process is curved, reflecting the expansion of the braincase in the otic region, and is aligned horizontally, forming a slightly obtuse angle with the shaft of the quadrate (Fig. 25A). At the anterodorsal corner produced between the two structures, there is a small, pointed, and broadly triangular expansion that extends anteriorly. That expanded corner is level with the dorsal margin of the bone. The caudal process is approximately twice the length of the shaft. At its anterior origin, the caudal process is twice the width of the shaft, but tapers to a blunt, slightly upturned point posteriorly. The shaft of the quadrate ends ventrally in a rounded, expanded mandibular condyle. The condyle expands transversely, as well as anteroposteriorly, and is dumbbell-shaped with a shallow trochlea for articulation with the compound bone.

#### *Uropeltis rubromaculata*

The quadrate of *U. rubromaculata* does not differ substantially from that of *U. woodmasoni*. However, in articulated skulls, because there is less constriction of the retroarticular process of the compound bone in *U. rubromaculata*, the shaft appears longer relative to the caudal process, but the proportions are actually the same as in *U. woodmasoni*.

#### *Uropeltis melanogaster*

In one specimen (TMM M-10032), the caudal process is proportionately longer than the shaft, reaching 2.5 times the length of the latter. The second specimen, TMM M-10045, lacks this extra length and shows a stronger resemblance to *U. woodmasoni*, although both specimens of *U. melanogaster* have a more slender quadrate (Fig. 25B). The anterodorsal margin of the quadrate curves so that the anterodorsal corner is ventral to the dorsal margin of the bone. The angle between the caudal process and the shaft is more acute, roughly 90°.

#### *Rhinophis blythii*

The single specimen possesses a quadrate with a caudal process that is approximately 2.5 times the length of the shaft. Overall the bone is robust, as in *U. woodmasoni*.

#### *Rhinophis drummondhayi*

This taxon shows no notable difference from *U. woodmasoni*.

#### *Rhinophis homolepis*

The caudal process is slender, but the shaft is robust, creating the illusion that the caudal process is elongated, when in fact it is only about twice the length of the shaft.

#### *Rhinophis philippinus*

The quadrate is distinct from that of other *Rhinophis* and *Uropeltis* species examined in having a more sharply pointed caudal process and an anterodorsal corner that is more rounded and lacks a well-developed, triangular extension.

#### *Brachyophidium rhodogaster*

The quadrate is suspended from the braincase as in the other species examined. Overall the bone is more robust than in any *Uropeltis* or *Rhinophis* species examined and the caudal process is consistently about 2.5 times the length of the shaft. The dorsal margin of the caudal process displays a strong concave arch (Figs. 2C,25C). The difference is sufficient to allow for an easy distinction to be made between isolated quadrates of *B. rhodogaster* and those of *Uropeltis* and *Rhinophis* species. Additionally, the anterodorsal projection may be more prominent than in *U. woodmasoni*, but is rounded in *B. rhodogaster*, rather than triangular. Because of the curvature of the caudal process, the anterodorsal corner is ventral to the dorsal surface of the bone. An obtuse angle is formed between the caudal process and the shaft of the quadrate.

### The Uropeltid Mandible

In uropeltids the lower jaw consists of separate compound ( = articular of [32]), dentary, angular, splenial, and coronoid bones. The coronoid exhibits various degrees of development across taxa [19], but is generally not more than a thin chip of bone anchored to the medial surface of the coronoid process of the compound bone and is usually lost in disarticulated specimens. Posterior to the socket for articulation with the quadrate, there is a modified retroarticular process that is similar in morphology to that of *Anomochilus* [17], [49].

### Compound Bone and Coronoid

#### *Uropeltis woodmasoni*

**Figure 28:**
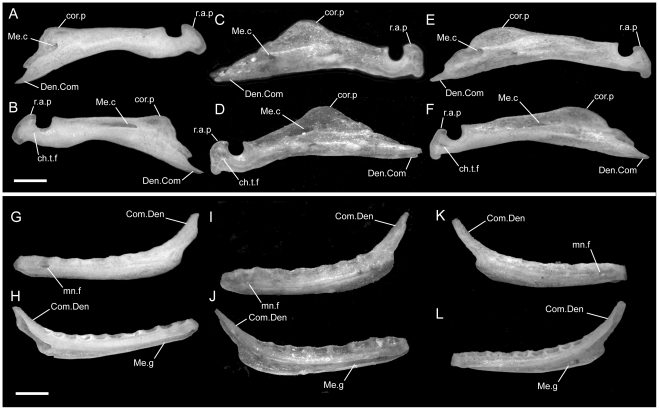
Disarticulated compounds and dentaries. Anterior is to the left in A,C,E,G,I,K,L; anterior is to the right in B,D,F,H,J,K; scale bars = 0.5 mm. Left compound bones in lateral (A,C,E) and medial (B,D,F) views. Left dentaries in lateral (G,I) and medial (H,J) views, and right dentary in lateral (K) and medial (L) views. A,B and G,H are from *U. woodmasoni* (TMM M-10001); C,D and I,J are from *U. melanogaster* (TMM M-10045); and E,F and K,L are from *B. rhodogaster* (TMM M-10022). ch.t.f = chorda tympani foramen; Com.Den = compound process of the dentary; cor.p = coronoid process of the compound; ct.f = foramen for the chorda tympani; Den.Com = dentary process of the compound; Me.c = Meckel's canal; Me.g = Meckel's groove; mn.f = mandibular foramen; r.a.p = retroarticular process of the compound.

The retroarticular process is well-developed, expanded, and rounded, and as it curves anterodorsally it partially constricts the socket for the mandibular condyle of the quadrate (Fig. 28A,B). In dorsal view, a shallow depression is followed posteriorly by a low ridge that forms the anterior lip of the socket. In other *Uropeltis* and *Rhinophis* species examined those features are more strongly developed. At its posterior end, the compound bone sandwiches the pterygoid between the jaw joint and the braincase. In lateral view, moving anteriorly, the dorsal and ventral margins of the compound bone are parallel, but less than halfway down the length of the bone the ventral margin begins to slope gradually downward. Farther along, immediately anterior to the center of the element, the dorsal margin expands into a small, rounded, triangular coronoid process (Figs. 2A; 28A,B). Anterior to that process the dorsal margin slopes sharply downward and ends in a pointed, slightly forked anterior tip. A forked anterior tip also occurs in *Plectrurus aureus* [19]. The anterodorsally inclined surface contacts the complementary sloped posteroventral margin of the dentary. A small foramen occurs ventral to the anterior half of the coronoid process.

In medial view, a foramen and deep groove for Meckel's cartilage [32] is ventral to the posterior margin of the coronoid process (Fig. 28B) and continues onto the dentary. In articulated skulls, the foramen is visible in lateral view (Fig. 2A). The compound bone is essentially hollow from its anterior tip to the coronoid process posteriorly, and the anterodorsal surface is deeply excavated to interlock with the dentary, which overlaps the compound bone laterally. The anterior tip of the compound bone extends forward to reach the suture between the angular and splenial. Medially, a foramen for the chorda tympani branch of the facial nerve (CN VII) occurs between the retroarticular process and the socket for the quadrate. The position of the foramen is the same as in *P. aureus* [19].

Medially (not visible in lateral view), a thin, small, and subcircular coronoid bone rests flat against the coronoid process of the compound bone. The anterior edge of the chip of bone contacts the dentary. In TMM M-10021, where the coronoid bone was disarticulated, two tiny foramina are visible in the underlying compound bone and they connect to Meckel's canal internally. Only one foramen occurs in TMM M-10001.

#### *Uropeltis rubromaculata*

Overall, the compound bone strongly resembles that of *U. woodmasoni*. The retroarticular process, however, is much more rounded and circular, although its dorsal surface does not seem to constrict the socket for the quadrate. A small, thin coronoid bone is present, and in lateral view a foramen is ventral to the anterior half of the coronoid process, although it is located more ventrally than in *U. woodmasoni*.

#### *Uropeltis melanogaster*

The retroarticular process of the compound is irregularly shaped and does not constrict the socket for the mandibular condyle. However, contrary to the condition in *U. woodmasoni* and *U. rubromaculata*, the dorsal surface of the retroarticular process is much broader and larger than the ventral (Fig. 28C,D). In medial view a foramen pierces the center of the retroarticular process, but there is not one below the coronoid process. Additionally the coronoid process is developed into a larger, taller, triangular process with a wide base that extends posteriorly to the socket for the quadrate as a laterally compressed, downward sloping crest. The massive coronoid process is also located more posteriorly, and the articulation surface for the dentary is longer and less steeply inclined than in *U. woodmasoni*. The coronoid bones of both individuals presumably became disarticulated and were lost before we studied the specimens.

#### *Rhinophis blythii*

In lateral view, the entire dorsal margin of the compound bone is arched from the posterior extent of the dentary articulation to just anterior to the articulation with the quadrate, forming a large, broad, rounded coronoid process (Fig. 8A). This gives the compound bone a wide and smooth appearance at its midpoint. A tiny coronoid bone is present, but unlike in *U. woodmasoni*, a sliver of the bone is visible in lateral view along the dorsal margin of the compound bone.

#### *Rhinophis drummondhayi*

Unlike *R. blythii*, *R. drummondhayi* has a distinct, broadly triangular coronoid process in lateral view. The retroarticular process of our specimen constricts the socket for the mandibular condyle of the quadrate, and the dorsal surface is narrow, blunt, and rounded. The ventral surface is continuous with the ventral margin of the body of the compound bone, forming an expanded, bluntly angled corner.

#### *Rhinophis philippinus*

The coronoid process of the compound bone is broad, and somewhat triangular. A foramen pierces the medial surface of the retroarticular process, but none occurs ventral to the coronoid process. The retroarticular process is like that of *U. woodmasoni*, and both the dorsal portion of the process and the anterior rim of the socket for the quadrate constrict the cotyle.

#### *Rhinophis homolepis*

The retroarticular process of the compound bone is narrow, but well-developed and rounded. There is no foramen visible in the retroarticular process. The anterior lip of the socket for the quadrate is greatly constricted, but the posterior lip is only moderately constricted. There is no distinct coronoid process in lateral view because the dorsal margin of the compound is only shallowly arched.

#### *Brachyophidium rhodogaster*

In lateral view the compound bone exhibits less curvature of the ventral surface than that of other species of *Uropeltis* or *Rhinophis* except *R. homolepis* (Figs. 2C;28E,F). The dorsal and ventral surfaces are horizontal and parallel for a much longer distance, with the bend occurring much farther anteriorly and at the same level dorsally and ventrally. In lateral view, the coronoid process is low, broad, and hemispherical. When articulated, less of the mandibular condyle of the quadrate is visible, indicating that the socket overlaps more of the condyle's lateral surface. The ventral part of the retroarticular process is narrow, rounded, and blunt while the dorsal half is broader. However, the shape and size of the retroarticular process, as well as the degree of constriction of the dorsal surface, varies widely among specimens. In one specimen (TMM M-10023) the dorsal part of the retroarticular process hooks anteriorly over the cotyle, constraining the socket greatly. In TMM M-10027 the end of the process is T-shaped. In all specimens there is a foramen for the chorda tympani nerve, although it is more anterior than in the species of *Uropeltis* and *Rhinophis* examined. The depression and ridge anterior to the cotyle are weakly developed, as in *U. woodmasoni*. A thin, sub-circular coronoid bone is present, but no foramina occur ventral to the coronoid process.

### Angular and Splenial

#### *Uropeltis woodmasoni*

The angular is a triangular bone that is sharply pointed posteriorly. It contacts and clasps the ventromedial margin of the compound bone from the anterior tip of the compound bone to just ventral to the coronoid process. The anterior tip of the angular, which participates in a vertical, straight contact with the splenial, is cylindrical is cross section. When disarticulated, the angular has a concave dorsal surface as well as a tiny foramen and crest located on the anterior tip of the ventral surface. The ventral surface of the angular is convex. The splenial mirrors the angular in shape, tapering from a cylinder posteriorly to a sharp point anteriorly. The bone possesses a large foramen medially, approximately two-thirds of the way from the anterior to the posterior end. The splenial clasps the ventromedial surface of the dentary, ending anteriorly at the midpoint of the latter. Additionally, only a sliver of the splenial is visible in lateral view, because it does not wrap around as far laterally as the slightly longer angular.

#### *Uropeltis rubromaculata*

The angular extends posteriorly to below the center of the coronoid process. Clear foramina occur in both the angular and splenial, and the foramen in the angular is located more ventrally than in *U. woodmasoni*.

#### *Uropeltis melanogaster*

The angular lacks the ventral convexity observed in *U. woodmasoni*. Instead, that surface of the bone is rough and irregular and has an anteroposteriorly aligned, low ridge along the midline. Medial to the ridge is a dent that may contain a nearly closed foramen at its center. The splenial is like that of *U. woodmasoni*.

#### *Rhinophis blythii*

As in *U. rubromaculata*, the angular extends to the midpoint of the coronoid process of the compound bone. The splenial does not differ from that of *U. woodmasoni*.

#### *Rhinophis drummondhayi*

The angular and splenial have the same general morphology of those of *U. woodmasoni*.

#### *Rhinophis philippinus*

The splenial is as large as the angular, and the dentary overlaps both in lateral view. In the other taxa, there is much less lateral overlap of the splenial by the dentary.

#### *Rhinophis homolepis*

The splenial and angular do not differ substantially from those of *U. woodmasoni*.

#### *Brachyophidium rhodogaster*

The angular reaches the posterior half of the coronoid process and has a small, ventral foramen. The foramen found in the splenial is located closer to the angular-splenial suture than in species of *Uropeltis* and *Rhinophis*.

### Dentary

#### *Uropeltis woodmasoni*

The dentary has a posterior tip that is blunt but tapered and which ends posteriorly just anterior to the peak of the coronoid process of the compound bone. The ventral surface of the posterior tip has a groove, possibly to facilitate articulation with the compound bone. In medial view, the groove for Meckel's cartilage continues from the compound bone, extends along the ventral half of the medial surface of the dentary, and terminates just posterior to the anterior tip of the latter element (Fig. 28H).

The rounded anterior tip of the dentary curves medially to meet the other dentary at a midline juncture composed mostly of soft tissue. The ligament between the two is not broad as in macrostomatans and probably restricts movement. The teeth on the dentary originate posterior to the anterior tip, and the edentulous space preceding them is approximately the size of half a tooth socket. The tooth row ends posteriorly at the level of the suture between the angular and splenial. Ten dentary teeth are present in most individuals, although TMM M-10003, TMM M-10005, and TMM M-10010 possess nine, and TMM M-10002 has ten on the right side and nine on the left (Table 1). The dentary teeth are shaped like the maxillary teeth, and the largest tooth occurs at or just anterior to the midpoint of the dentary. In lateral view, a single, large mandibular foramen is located near the anterior end of the dentary, just posterior to the start of the medial curvature of the element (Fig. 28G). In clean disarticulated specimens a small, medial knob also is visible at the anterior tip, and is probably an attachment point for the mandibular ligament.

#### *Uropeltis rubromaculata*

The dentary is similar to that of *U. woodmasoni*, except in the case of the teeth, which extend farther posteriorly; two full sockets are located posterior to the angular-splenial suture. Eight teeth occur on the dentary; like the maxillary teeth, they are enlarged relative to those of other taxa examined (Fig. 2B). Our tooth count is higher than a previous report of six or seven dentary teeth occurring in specimens of *U. rubromaculata* [15].

#### *Uropeltis melanogaster*

Other than a smoother and straighter process for the compound, a more sharply pointed posterior tip, and the possession of less edentulous space posterior to the anterior tip, the dentary does not differ substantially from that of *U. woodmasoni* (Fig. 28I,J). Eight teeth are present.

#### *Rhinophis blythii*

The dentary has a much more sharply pointed posterior tip than that of *U. woodmasoni*, and the teeth end posteriorly at the splenial-angular suture. The groove for Meckel's cartilage is open in anterior view, and this creates a medial, trochlea-like expansion of the anterior tip of the dentary. Eight teeth are present.

#### *Rhinophis drummondhayi*

As in *U. woodmasoni*, the teeth terminate posteriorly at the angular-splenial suture. Eight teeth are present.

#### *Rhinophis philippinus*

The posterior end of the dentary is much more pointed than in *U. woodmasoni*, and the last tooth occurs at the splenial-angular suture. Seven teeth are present.

#### *Rhinophis homolepis*

The last tooth position occurs anterior to the angular-splenial suture. There are nine tooth positions.

#### *Brachyophidium rhodogaster*

The sloped surface of the posterior end of the dentary is steeper and proportionately longer than in the other species examined (Fig. 28K,L). This results in a posterior tip that is more sharply pointed than in *U. woodmasoni*. The tooth row terminates anterior to the angular-splenial suture. In TMM M-10018, a small flange projects medially from the anterior end of the dentary, but this feature is not observed in any other specimen. The majority of specimens have ten teeth on the dentary. Two specimens (TMM M-10017, -10025) have nine teeth on the right side and ten on the left, and TMM M-10011 has positions for 12 teeth.

### Phylogenetic Analysis

**Figure 29:**
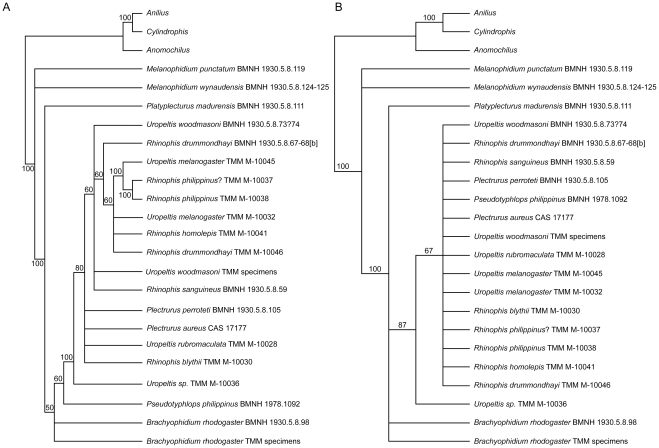
Majority Rule Consensus trees (50%) for the phylogenetic analysis of morphological characters for 16 uropeltid species. (A) All 33 characters included (see Methods S2); (B) characters 4,6,7,11,13 excluded.

**Figure 30:**
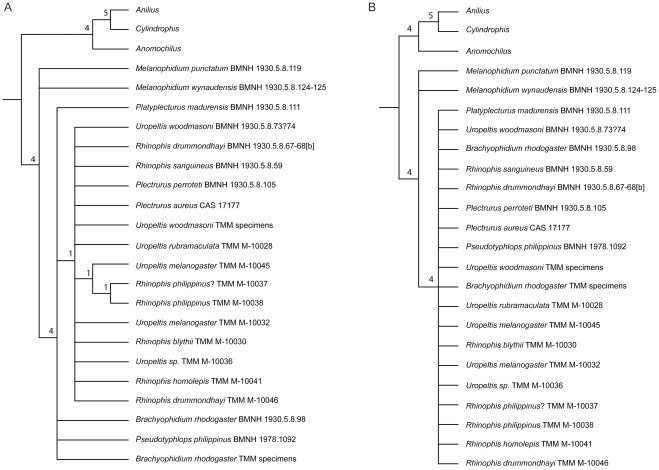
Strict Consensus trees with Bremer support values for the phylogenetic analysis of morphological characters for 16 uropeltid species. (A) All 33 characters included (see Methods S2); (B) characters 4,6,7,11,13 excluded.

Analysis of the relationships of 16 species of uropeltids resulted in 80 Most Parsimonious Trees (MPTs; Tree Length = 85, CI = 0.717, RI = 0.8110). The Majority Rule Consensus of those trees indicates that relationships are not well-resolved, although a small number of clades are highly supported (Fig. 29A). The larger individual of *U. melanogaster* (TMM M-10045) is the sister taxon to a monophyletic *R. philippinus* (100% of topologies). The position of *R. blythii* is variable, however, and that taxon may fall outside a clade containing the other three species of *Rhinophis*. Neither *Rhinophis* nor *Uropeltis* are monophyletic, and the monophyly of *Melanophidium*, *Brachyophidium*, and *Plectrurus* is not established. However, a clade including species of *Uropeltis*, *Rhinophis*, and *Plectrurus*, to the exclusion of *Brachyophidium*, *Platyplectrurus*, *Pseudotyphlops*, and *Melanophidium*, is recovered in 100% of topologies. In 60% of topologies, *Pseudotyphlops philippinus* is the sister taxon to the clade that exclusively contains species of *Uropeltis*, *Plectrurus*, and *Rhinophis*. Consistent with both previous molecular and morphological analyses, the two species of *Melanophidium* are supported strongly as the outgroup to the remaining uropeltid taxa (100% of topologies). The Strict Consensus exhibits less resolution, but a high Bremer support value also upholds the placement of the *Melanophidium* taxa (Fig. 30A). A clade that includes only the species of *Uropeltis*, *Rhinophis*, and *Plectrurus* is also retained in the Strict Consensus, but with low Bremer support. Similarly, the sister relationship between the larger specimen of *U. melanogaster* and a monophyletic *R. philippinus* is also recovered, but weakly supported.

When the six characters that exhibited the highest degree of polymorphism and individual asymmetries (i.e., Characters 4,6,7,11,13) were removed from the analysis, 469 MPTs were recovered (Tree Length = 49, CI = 0.7755, RI = 0.8791). As indicated by the Majority Rule Consensus (Fig. 29B), a clade comprising species of *Uropeltis*, *Rhinophis*, and *Plectrurus* was recovered in most topologies (87%). However, in both the Majority Rule and Strict Consensus, resolution of relationships other than the position of species of *Melanophidium* was extremely poor, suggesting that the excluded characters carry phylogenetic signal and should be revised rather than discarded (Figs. 29B,30B).

## Discussion

### Comparative Osteology and Morphological Variation

Uropeltids have a notably different skull morphology from other snakes [55]. All known uropeltid species are small (<80 cm; e.g., [61]) and fossorial, which is reflected in a general morphological similarity among uropeltid skulls. Comparison of cranial morphology among taxa we examined and those reported in the literature [17], [19], [31], [32], [34] suggests that the major contacts between bones and the overall relationships among articulated elements do not differ substantially among taxa. Most of the morphological variation that does occur is related to either shape or proportional differences for particular elements. The pattern of cranial fenestration also is variable both within and among uropeltid taxa (see [55] and this study].

As a result of the general similarity in skull morphology among uropeltids, isolated elements from different taxa often lack distinctive features (i.e., apomorphies). This is especially true for simple bones, such as the ectopterygoid and pterygoid. However, more complex elements such as the premaxilla, septomaxilla, and nasal, are distinctive enough to be recognized for particular taxa in some cases. The morphology of many elements and structures of *Brachyophidium rhodogaster*, for example, is distinct from that in the species of *Uropeltis* and *Rhinophis* examined by us, although bones and structures from the latter two often are indistinguishable from one another. That pattern is not surprising, because previous morphological and molecular phylogenetic analyses as well as our analysis (Fig. 29) suggested that, given current taxonomy, either *Rhinophis* is paraphyletic with respect to *Uropeltis* [17] or that both *Uropeltis* and *Rhinophis* are paraphyletic [20], [21]. Based on phylogenies reconstructed from immunological [20] and genetic [21] data, it was hypothesized that the Sri Lankan uropeltid species radiated from a single invasion from India and are monophyletic, regardless of current generic assignment [22]. Results from our morphological phylogenetic analysis at least partially support that hypothesis because the larger, presumably adult, specimen of the Sri Lankan *U. melanogaster* is most closely related to the Sri Lankan *R. philippinus* in all MPTs.

Although morphological data suggest that *B. rhodogaster* is positioned outside of a clade that exclusively includes species of *Plectrurus*, *Uropeltis*, and *Rhinophis* (see [17], this study), molecular data yielded hypotheses under which *B. rhodogaster* either nested within a deeply paraphyletic *Uropeltis* [21] or was the sister taxon of at least one sampled species of *Uropeltis* [20]. Although many of the morphological features that we described for *B. rhodogaster* may be plesiomorphic for uropeltids, the broader distribution of most features remains unknown because of the lack of detailed studies on the majority of the species of *Rhinophis* and *Uropeltis*, and inadequate knowledge of variation within *Platyplectrurus*, *Pseudotyphlops*, and the genus consistently recovered as sister to all other uropeltids, *Melanophidium* (per the results in this study as well as [17], [20], [21]). As a result, it is unclear if some of the more disparate morphologies described for *B. rhodogaster* are autapomorphies rather than plesiomorphies common to other uropeltids. We consider one specimen of “*B. rhodogaster*” (TMM M-10025) to have been misidentified previously. That individual was disarticulated entirely before we acquired it for study, but the morphology of the isolated elements is inconsistent with that of all other *B. rhodogaster* we examined. Features such as the bipartite rostrum of the premaxilla, long finger-like premaxillary process of the nasal, and lack of an anterior process on the maxilla indicate a relationship with species of *Uropeltis*, *Rhinophis*, *Plectrurus*, or a closely related but unexamined taxon. Another specimen, TMM M-10036, was identified originally as *Uropeltis* sp., but the presence of a keeled interchoanal process, lack of a groove on the dorsum of the crista trabecularis and absence of a palatine process of the vomer point to an affinity with *U. melanogaster* or *P. aureus* (figs. 12,25 of [19]), although it does not exclude the possibility that it is a new or unstudied species. In most topologies recovered in our phylogenetic analysis, TMM M-10036 fell outside of the clade containing other species of *Uropeltis*, *Rhinophis*, and *Plectrurus*, but always was more closely related to those taxa than to species of *Brachyophidium*, *Platyplectrurus*, *Pseudotyphlops*, or *Melanophidium* (Fig. 29). The case of this enigmatic specimen highlights the fact that ultimately all biological studies of uropeltids are at the mercy of a demonstrably inadequate taxonomy. That problem is exacerbated for skeletal material without associated tissue samples or intact skin.

Left-right asymmetry of both foramina and minor contacts is common among the individuals of the taxa examined in our study and also was noted in a prior study of endocasts of TMM M-10006 [51]. Although asymmetry of the contacts between bones may be attributed to differential drying of skulls during preparation, similar asymmetry in fenestration cannot be, and thus provides evidence for a high degree of variability in uropeltids. Intraspecific variation and asymmetries have direct implications for character identification and scoring. This is especially important for phylogenetic analyses of uropeltids, which previously relied predominantly on small sample sizes. In our study, a high degree of variation was discovered in taxa even when the total sample size equaled two specimens (Table S2, Fig. 29). That, however, could be the consequence of other common challenges to research on uropeltids, which include uncertainties in taxonomic arrangements, misidentification of specimens, rampant problems with synonymy, and a lack of data about development, sexual dimorphism, and patterns of geographic variation. The lack of developmental information is particularly important because many of the more disparate individuals differ in size from their conspecifics, and variation related to ontogeny versus that resulting from phylogeny (the presence of multiple, perhaps unknown, species) cannot be distinguished from one another without future developmental research.

The most convincing example of ontogenetic variation in our sample occurs between the two specimens of *U. melanogaster*. We are relying on species identifications provided at the time of collection, but the fact that the individuals were collected from the same locality (Table S1) supports the hypotheses of conspecificity. The two *U. melanogaster* vary in morphology for almost all skull elements, and in nearly all cases processes or structures present in the larger specimen (TMM M-10045) were smaller or absent in the smaller individual (TMM M-10032). For example, the septomaxilla of TMM M-10032 has smaller and rounded anterodorsal and anteroventral processes of the lateral wall, and the palatal tubercle and crest surrounding the vomeronasal cupola are practically absent. If those characters were scored in a matrix, TMM M-10032 might be reported to possess different character states than TMM M-10045. In fact, those two individuals were scored differently for characters 12 and 13, and were not supported as sister taxa in our phylogenetic analysis (Figs. 29,30). Until ontogenetic transformations are documented for a wider range of taxa, the recognition of these two individuals as conspecifics is only tentative. Future work may show that particular structures, proportions, and morphological features vary phylogenetically, ontogenetically, or sexually.

Both *U. woodmasoni* and *B. rhodogaster* exhibit the highest levels of polymorphism in our study, in some cases possessing all possible character states for a given character (Table S2, Methods S2). That is not surprising, however, given that many more specimens were available for those two taxa. The variation in *U. woodmasoni* and *B. rhodogaster* does not appear to be bimodal (within the limited sample sizes), and therefore is unlikely to be related to sexual dimorphism, although that phenomenon has not been studied in uropeltids. Additionally, all TMM specimens of *U. woodmasoni* were collected from the same locality, reducing the possibility for geographic variation (Table S1). Most TMM specimens of *B. rhodogaster* also were collected from a single locality, although notably, no data were available for the potentially misidentified TMM M-10025, and TMM M-10026 and TMM M-10027 are listed only as from ‘S. India’ (Table S1). Note that our score for the absence of the interchoanal process in *B. rhodogaster* differs from that reported previously for a specimen of *B. rhodogaster* originally identified as ‘*Teretrurus rhodogaster*’ (BMNH 1930.5.8.59) [17]. That and other scoring differences (Methods S2), as well as the results of our phylogenetic analysis, suggest that our *B. rhodogaster* and that specimen of ‘*T. rhodogaster*’ may not be conspecific. It is possible that either BMNH 1930.5.8.59 is *Teretrurus sanguineus* or that our specimens were misidentified, although nearly all TMM specimens of *B. rhodogaster* were collected from the type locality for *B. rhodogaster* [1]. Locality data may provide support for species identifications, because species of uropeltids appear to have narrowly restricted ranges in places where populations were best studied (e.g., Sri Lanka), [22]. The complex taxonomic history of *Brachyophidium* and *Teretrurus* [1], as well as problems recognized previously for species of *Uropeltis* [15], highlights the need for re-evaluation of uropeltid taxonomy, including examination of the morphology and provenance data of individual historical and type specimens.

### Implications for Phylogenetic Analyses

A useful starting point for identification of phylogenetically informative morphological characters of uropeltids was provided by previous authors [17]. As more specimens and taxa become available for study, proposed characters will need to be tested, modified, and expanded. Three of the taxa surveyed in our study (*U. woodmasoni*, *R. drummondhayi*, and *B. rhodogaster*) were also studied previously [17], but with larger sample sizes we were able to assess intraspecific variation. In some cases, the usefulness of previously recognized characters was supported, while others were found to be highly variable intraspecifically. At least seven (4,6,7,10,13,15,16) of the 33 characters proposed originally [17] expressed some degree of individual variation or polymorphism. We also found left-right asymmetries within single individuals for four of those characters (4,7,15,16). Individuals of two species, *U. woodmasoni* and *U. rubromaculata*, exhibited left-right asymmetries for Character 15, and those same taxa, as well as *R. blythii*, exhibited asymmetries for Character 4. Characters 4 and 7 address elements that articulate only weakly when in contact, and thus it is likely that the asymmetries are caused by differential drying during skeletonization. Characters 15 and 16 refer to divisions of foramina, which are not susceptible to variation owing to drying. However, in some individuals the divisions occur deeply and thus are likely to be scored differently when internal data from CT scans and X-rays are available to complement standard external, surface examination. Many of the minute details we observed would be difficult to see and score in standard museum material. Immaculately clean skeletal preparations or CT models are required for the elucidation of such fine detail. In other cases, as more taxa are examined, characters may need to be re-written or expanded in order to encompass the full range of morphological expression found in uropeltids. Character 4 describes a ‘well-defined buttressing contact’ between the anteromedial process of the maxilla and the anterolateral process of the vomer [17]. That description does not fit the condition found in any of our specimens, although the original authors reported it as present in *U. woodmasoni* and *R. drummondhayi* [17]. In all of the specimens that we examined, if a contact was present at all it was weak and by no means buttressing. Similar issues were discussed in an earlier description of *P. aureus* [19], in which issues involving Characters 1 and 26 [17] were highlighted also. As in *P. aureus*, for Character 1, *B. rhodogaster* possesses a definitive contact between the maxilla and premaxilla, but the articulation is not straight or buttressing as implied by the term ‘schizarthrotic’ in the original character description and scoring for that taxon (state 1). Instead, the articulation is more complex; a clasping contact is formed between the two bones because the transverse process of the premaxilla inserts into the space between the anteromedial process and the anterior tip of the maxilla. Additionally, the maxilla has a short, anterior process that overlaps the transverse process of the premaxilla dorsally. Character 26 describes the contact between the vomer and premaxilla; the two bones may meet in an overlapping contact (state 0), or abut one another within a well-defined recess (state 1). Although it is true that in all the uropeltids we examined the elements meet within a well-defined recess, the premaxillary process of the vomer also overlaps the premaxilla in all individuals of *P. aureus* [19], *B. rhodogaster*, *R. blythii*, *U. woodmasoni*, and perhaps *U. melanogaster*. Also, *Cylindrophis* (treated as a supraspecific taxon by [17]) previously was scored as possessing a condition similar to uropeltids (state 1) [17], but the actual condition in *Cylindrophis rufus* (UCMP 136995; pers. obs. JCO) is the same as in *Anilius* (state 0).

Character 14 [17] also may be insufficient as described currently. That character describes whether the jugular foramen is located fully behind the juxtastapedial recess (state 0), or recessed within the juxtastapedial recess (state 1). According to the original scoring [17], all sampled species of *Rhinophis* and *Uropeltis* expressed state 1, whereas *B. rhodogaster* had state 0. However, we found interpretation and scoring of Character 14 to be difficult once a wider range of taxa and individuals were examined. In *U. woodmasoni*, the jugular foramen is contained within a cup-like recess that, although separate from the juxtastapedial recess, is still formed by the margin of the crista-circumfenestralis (Methods S1). Our specimens of *Uropeltis rubromaculata*, *R. blythii*, and *R. philippinus* share that condition with *U. woodmasoni*. Scoring of *B. rhodogaster* for Character 14 also presented challenges. In most individuals the foramen is not located within a well-defined cup, but nonetheless is recessed within the margins of the crista circumfenestralis and is separated from the juxtastapedial recess proper by a low wall of bone (state 1). Our specimens of *R. homolepis* and *R. drummondhayi* also exhibit that intermediate condition. Finally, individuals of *U. woodmasoni*, *R. philippinus*, and *B. rhodogaster* exhibit morphologies intermediate to the states described previously for Character 11, whether the laterosphenoid is ‘narrow’ (state 0) or ‘broad’ (state 1) [17]. All of these issues demonstrate that as a broader sample of taxa and individuals are studied, characters will need to be redescribed and expanded to incorporate the growing range of known morphology among uropeltids. In our analysis many of those characters were found to be phylogenetically informative, providing resolution among higher groups of uropeltids (Figs. 29,30), but are insufficient and difficult to utilize as currently described.

Other characters proposed previously [17] were upheld by testing and were not subject to intraspecific variation, asymmetries, or intermediate morphologies in our study. Many of those characters may turn out to be important features for distinguishing clades within Uropeltidae. For example, we observed that the nasal tapers to a pointed tip anteriorly (Character 2, state 1) in all examined specimens of *Uropeltis* and *Rhinophis*. That morphology also is characteristic of *Plectrurus aureus* [19], *Plectrurus perroteti* and *Pseudotyphlops philippinus* [17]. Similarly, all individuals of *B. rhodogaster* have a nasal that is broad anteriorly with a small notch (Character 2, state 0). Character 5, whether the parietal does (state 0) or does not (state 1) participate in the optic foramen, has the same distribution as Character 2, with the exception of *P. philippinus* and *Melanophidium punctatum* sharing state 0 with *B. rhodogaster*. Character 17 also appears to be phylogenetically informative; *B. rhodogaster*, *P. madurensis*, and both *Melanophidium* possess a short stalk for the occipital condyle (state 0), but all other uropeltids examined by us and other authors [17], [19], have a long stalk (state 1). Similarly, Character 18, whether the posteroventral process of the dentary is distinct (state 0), reduced (state 1), or absent (state 2), exhibits invariant conditions in the taxa that we examined, and specific morphologies are characteristic of particular taxa. *Melanophidium* is the only taxon that possesses state 0, while *P. madurensis* and *P. perroteti* exhibit state 1 [17]. However, *Plectrurus aureus* [19] and all other uropeltid taxa examined by us and prior authors [17] show state 2. All remaining characters unaffected by intraspecific variation or other scoring issues separate only *Melanophidium* from the rest of the uropeltids (Characters 8,9, 29) or the uropeltids from *Anilius*, *Anomochilus*, and/or *Cylindrophis* (Characters 19–25,27,28,30–33).

In addition to the characters outlined previously [17], a number of features described herein are identified for future testing and possible use in phylogenetic analyses. In particular, the nature of the skeletal material available to us allowed for investigation of the potential utility of disarticulated elements as a source of phylogenetically informative data. A few of the thirty features are discussed in detail below; refer to Methods S4 for additional features and their distribution among the specimens we examined.

One possible new character is the morphology of the interchoanal process extending anteroventrally from the cultriform process. In the uropeltids that we examined, as well as in illustrations of *Melanophidium wynaudense* and *Rhinophis sanguineus* (figs. 2.25,2.31 [55]), when the process occurs it is sharply triangular (i.e., keel-shaped). In *Plectrurus aureus*, the ‘keel’ was described incorrectly as separate from the interchoanal process (fig. 25 [19]), although as in *Cylindrophis rufus* (UCMP 136995; pers. obs. JCO), an associated triangular, ventral projection also occurs more posteriorly (the additional projection is more prominent in *C. rufus*). Illustrations of the process in *Anomochilus*, in contrast, suggest that the interchoanal process is hook-shaped and more rounded in that taxon (fig. 2.20 [55]; fig. 5 [49]), whereas in *C. rufus* the process is straighter and almost needle-like (pers. obs. JCO). The wider taxonomic distribution of that feature in unknown currently, although the interchoanal process clearly is absent in our specimens of *B. rhodogaster*. In contrast to previous reports [17], however, a triangular interchoanal process occurs in *Anilius scytale*, and as in *C. rufus* (though less well-developed), an associated posterior projection also occurs (pers. obs. JCO). In *A. scytale* the interchoanal process extends beyond the cultriform process, anteriorly, but in *C. rufus* the two processes are of approximately equal length (UCMP 136995; pers. obs. JCO).

Another potentially useful characteristic is the presence of an elongate palatine process of the vomer, present in all specimens of *B. rhodogaster* and *U. woodmasoni* we examined, as well as in our specimen of *U. rubromaculata*. The process may be absent in *M. wynaudense*, *R. sanguineus*, and *P. philippinus* (see fig. 2 of [17]), suggesting that absence is the ancestral condition. All specimens of *U. melanogaster* and *Rhinophis* that we examined also lack the process, as does *P. aureus* [19]. That pattern is interesting because, as noted above, *Uropeltis* and *Rhinophis* may be paraphyletic (see [17], [20], [21] and this study), and the species from Sri Lanka may be monophyletic regardless of current generic assignment [20]–[22]. The species of *Uropeltis* and *Rhinophis* that we examined share more derived features with one another than with *B. rhodogaster*, but the majority of the other species assigned to *Rhinophis* and *Uropeltis* have never been studied. All potentially informative characters require more testing through broader taxonomic sampling, larger samples of individuals for most taxa, and studies of skeletal development.

### Miniaturization, Fossoriality, and Phylogeny

Fossoriality and miniaturization frequently occur together in vertebrates [62], although a causal relationship between the two phenomena is not well-established. However, recognition of the presence of those phenomena has important implications for phylogenetic analyses because morphological features that are the result of shared ancestry must be distinguished from those derived from shared functional and developmental constraints [63]. Homoplastic morphology frequently results from miniaturization of vertebrates because of similar problems and constrained solutions associated with size reduction [64]. That may be one reason why phylogenies based on osteology alone tend to group uropeltids and *Anomochilus* together [2], [4], while those based on molecular or combined evidence (osteology, plus molecular or soft tissue data) are more variable (e.g., [2], [6], [9], [11], ). Morphological features associated with burrowing and small size may be exacerbated in uropeltids and clades hypothesized to be their close relatives (e.g., *Anomochilus*) if those features were overprinted on existing modifications retained from an earlier period of fossoriality and size-reduction hypothesized to have played a role in the origin of snakes [55], [65]. Potential evidence for miniaturization from endocasts of soft tissues in *U. woodmasoni*, such as the relative enlargement and compact morphology of the sensory regions of the brain and inner ear, was discussed previously [51]. Our osteological descriptions provide additional support for features related to miniaturization in at least some species of uropeltids.

Miniaturized or size-reduced vertebrates tend to show a reduction in cranial ossification as well as increased mineralization of cartilage [64], [66]–[69], although those modifications do not occur in all miniaturized taxa [64], [67], [70]. For the most part, bone loss or reduction is not exhibited by uropeltids, which have highly ossified skulls that tend toward fusion (see [17], [55] and this study). The only bone that is lost in all uropeltids is the supratemporal, which also is lost in the burrower *Anomochilus leonardi* [71] (but not *A. weberi*, see [49]). Additionally, both uropeltids and species of *Anomochilus* lack teeth on elements that are toothed in other alethinophidian snakes (e.g., premaxilla, pterygoid). Reduction or loss of dentition is another feature shared among miniaturized or size-reduced taxa [63], [66].

The appearance of novel morphological conditions also is associated with miniaturization. One cranial region frequently modified in miniaturized vertebrates is the arrangement of the jaw muscles and suspensorium [64], [66], [67], [72], [73]. If the ear is relatively larger as a result of size-reduction of the skull, ancestral jaw mechanics may be impeded and a new arrangement of the suspensorium may result. That type of re-organization occurs in uropeltids and *Anomochilus*. In addition to the loss of the supratemporal in uropeltids and *A. leonardi* [71], the suspension of the quadrate is modified so that the bone articulates directly with the ventrolateral portion of the otic capsule. That positional change is reminiscent of many size-reduced animals where ‘verticalization’ of the suspensorium occurs and the jaw articulation is no longer visible in dorsal view [70], [72]. Furthermore, the morphology of the quadrate in both *Anomochilus* and uropeltids is modified to exhibit an elongate suprastapedial process not found in any other snake (Fig. 25A) [17]. In addition to the ventral transformation, the jaw suspension is shifted anteriorly in uropeltids and *Anomochilus*, a condition present in many miniaturized groups [63], [64], [70], [74]. The strong similarity between uropeltids and *Anomochilus* in these features may be due to a shared ancestry involving miniaturization, but it could also be the result of independent size-reduction within each lineage. Further systematic work is required, especially the evaluation of morphological features within a phylogenetic framework provided by molecular or combined analyses. That approach would reduce the circularity and bias associated with using morphological features correlated with fossoriality and/or miniaturization to reconstruct relationships.

### Conclusion

Although uropeltids share a superficially similar, highly derived cranial morphology, phylogenetically informative morphological variation does exist. Some previously proposed morphological characters are insufficient to capture a broader range of inter- and intraspecific variation, but can and should be expanded and modified rather than discarded. Among the taxa that we examined, *B. rhodogaster* possessed the most distinctive, and potentially autapomorphic, morphology for cranial elements, especially the premaxilla, maxilla, nasal, and braincase complex. Species of *Uropeltis* and *Rhinophis* often cannot be distinguished from one another morphologically, and potential apomorphies were shared by mixed groupings of species from both genera. Both that character distribution and results from our preliminary phylogenetic analysis support widespread previous indications of paraphyly within nominal genera of uropeltids, particularly *Rhinophis*, *Uropeltis*, *Plectrurus*, and *Melanophidium*. However, our analysis supports the existence of a clade composed exclusively of species of *Uropeltis*, *Rhinophis*, and *Plectrurus*, and upholds previous results suggesting species of *Melanophidium* to be successive outgroups to all other uropeltid taxa. Results from our analysis also partially support the hypothesis that Sri Lankan uropeltid species radiated from a single invasion from India and are monophyletic, regardless of current generic assignment.

Increased sampling of individuals within all of the taxa examined by us demonstrated a substantial degree of intraspecific variation, which has a large impact on the utility of morphological characters proposed previously for phylogenetic analysis. In addition to variation encountered among individuals for some characters, especially those related to divisions of foramina or delicate contacts between elements, asymmetries often were present within a single individual. Other features, such as the shape of the nasals, length of the occipital condyle, degree of development of the posteroventral process of the dentary, participation of the parietal in the optic foramen, presence of an interchoanal process, and presence of an elongate palatine process of the vomer, do not appear to be susceptible to high levels of intraspecific variation and may be phylogenetically useful. Additionally, we identified thirty morphological features that vary interspecifically within our sample, and may be of use in future phylogenetic assessments of the group. Seven of those require disarticulated material, and all require further testing with a larger and taxonomically more diverse sample.

Overall, our study highlights the need to examine additional taxa, especially rarer ones, perhaps using noninvasive techniques like micro-CT that would preserve soft tissues and obviate the need to disarticulate small, fragile skeletons. As a further test of potentially informative morphological characters, studies of skeletal development are required to assess ontogenetic variation, which is unknown currently for any uropeltid taxon. Other sources of variation (e.g., geography, sexual dimorphism) also need to be explored, and those studies must be conducted in the context of a well-supported phylogenetic hypothesis. The development of such a well-supported hypothesis may be hampered by broader trends, for example many large-scale features of the skulls of uropeltids and their potential close relatives, like *Anomochilus*, may be associated with fossoriality and miniaturization, which complicates analyses of relationships within Uropeltidae and among lineages of alethinophidian snakes.

## Supporting Information

Table S1 **Expanded specimen data including collection locality and condition of specimen.**
(DOC)

Table S2 **Variation among taxa and individuals scored for previously proposed morphological characters.**
(DOC)

Methods S1 **Character descriptions.**
(DOC)

Methods S2 Character matrix for phylogenetic analysis of morphological characters in PAUP*.
(DOC)

Methods S3 Character matrix in NEXUS format for phylogenetic analysis of morphological characters in PAUP*.
(NEX)

Methods S4 **Descriptions of potential new characters.**
(DOC)
