# Supplementary material for: Comparative Skull Morphology of Uropeltid Snakes (Alethinophidia: Uropeltidae) with Special Reference to Disarticulated Elements and Variation
Source: PLoS One. 2012 Mar 8;7(3):e32450. doi: 10.1371/journal.pone.0032450 (PMC3297617; doi:10.1371/journal.pone.0032450)
Supplement: Table S1 — Expanded specimen data including collection locality and condition of specimen. (DOC) [file pone.0032450.s001.doc]

**Table S1. Expanded specimen data including collection locality and condition of specimen.** Locality information taken directly from specimen labels and ledgers provided by C. Gans. bc = braincase; ept = ectopterygoid; max = maxilla; pa = parietal; pfr = prefrontal; pl = palatine; pmx = premaxilla; pt = pterygoid; q = quadrate; smx = septomaxilla; vo = vomer.

| **TMM number** | **field number** | **original identification** | **locality** | **skull condition and comments** |
| --- | --- | --- | --- | --- |
| M-10001 | CG 1342 | *Uropeltis woodmasoni* | Shembaganur, Madura Dist., E. Ugarte, S. India | fully disarticulated |
| M-10002 | CG 1690 | *Uropeltis woodmasoni* | T.N. Shembaganus, E. Ugarde, dou., India | articulated |
| M-10003 | CG 1691 | *Uropeltis woodmasoni* | T.N. Shembaganus, E. Ugarde, dou., India | articulated |
| M-10004 | CG 1692 | *Uropeltis woodmasoni* | T.N. Shembaganus, E. Ugarde, dou., India | articulated |
| M-10005 | CG 1693 | *Uropeltis woodmasoni* | T.N. Shembaganus, E. Ugarde, dou., India | articulated |
| M-10006 | CG 1694 | *Uropeltis woodmasoni* | T.N. Shembaganus, E. Ugarde, dou., India | articulated |
| M-10007 | CG 1695 | *Uropeltis woodmasoni* | T.N. Shembaganus, E. Ugarde, dou., India | articulated |
| M-10008 | CG 1696 | *Uropeltis woodmasoni* | T.N. Shembaganus, E. Ugarde, dou., India | articulated |
| M-10009 | CG 1697 | *Uropeltis woodmasoni* | T.N. Shembaganus, E. Ugarde, dou., India | articulated |
| M-10010 | CG 1698 | *Uropeltis woodmasoni* | T.N. Shembaganus, E. Ugarde, dou., India | partially disarticulated: lower jaws, 1 max, pt, pl |
| M-10011 | CG 1699 | *Teretrurus rhodogaster* | T.N. Shembaganur, E. Ugarde, dou., India | articulated |
| M-10013 | CG 1801 | *Teretrurus rhodogaster* | T.N. Shembaganur, E. Ugarde, dou., India (gift) | partially disarticulated: lower jaw, 2 q, occipital |
| M-10014 | CG 1802 | *Teretrurus rhodogaster* | T.N. Shembaganur, E. Ugarde, dou., India (gift) | articulated |
| M-10015 | CG 1803 | *Teretrurus rhodogaster* | T.N. Shembaganur, E. Ugarde, dou., India (gift) | articulated |
| M-10016 | CG 1804 | *Teretrurus rhodogaster* | T.N. Shembaganur, E. Ugarde, dou., India (gift) | partially disarticulated: pmx, max, lower jaws, q, pt, pl, vo, smx, pfr |
| M-10017 | CG 1805 | *Teretrurus rhodogaster* | T.N. Shembaganur, E. Ugarde, dou., India (gift) | articulated, lower jaws and Q's off. |
| M-10018 | CG 1806 | *Teretrurus rhodogaster* | T.N. Shembaganur, E. Ugarde, dou., India (gift) | partially disarticulated: lower jaws, left max, pt, ept, smx |
| M-10019 | CG 1807 | *Teretrurus rhodogaster* | T.N. Shembaganur, E. Ugarde, dou., India (gift) | partially disarticulated: bc, pa, lower jaws, 2 q |
| M-10020 | CG 1808 | *Teretrurus rhodogaster* | T.N. Shembaganur, E. Ugarde, dou., India (gift) | articulated |
| M-10021 | CG 1978 | *Uropeltis woodmasoni* | Shembaganur, S. India | fully disarticulated |
| M-10022 | CG 1980 | *Teretrurus rhodogaster* | Shembaganur, S. India | fully disarticulated |
| M-10023 | CG 1981 | *Teretrurus rhodogaster* | Shembaganur, S. India | fully disarticulated |
| M-10024 | CG 1901 | *Teretrurus rhodogaster* | none provided | fully disarticulated |
| M-10025 | CG 2090 | *Teretrurus rhodogaster* | ‘no data' (C. Gans) | fully disarticulated |
| M-10026 | CG 2973 | *Teretrurus rhodogaster* | S. India | fully disarticulated |
| M-10027 | CG 2974 | *Teretrurus rhodogaster* | S. India | fully disarticulated |
| M-10028 | CG 5206 | *Uropeltis rubramaculatus* | India, Kerala, Munnar Pullivarsal Estate; elevation 4200 feet, 19-20°C | articulated |
|  |  |  |  |  |
| M-10030 | F 141; CB 3 | *Rhinophis blythii* | Langton Estate, 5 km SE of Talawakele, Sri Lanka; elevation 1358m, 22°C | articulated |
| M-10032 | AL 161; CB 2, 1 of 2 | *Uropeltis melanogaster* | Nicapota, Lemastota, [Sri Lanka ?]; elevation 700m | fully disarticulated and crushed before preparation (C.Gans), associated with individual CB2, 1 of 2, from a collection of vertebrae from two individuals |
| M-10036 | F 249; CB 21 | *Uropeltis sp.* | Gammaduwa Rd., (Hunukelta, [ 'Hunukatta' crossed out], Rattotta), Sri Lanka; elevation 750m | partially disarticulated: pmx, max, lower jaws, q; juvenile? |
| M-10037 | F 354; CB 22 | *Rhinophis philippinus (?)* | Gammaduwa Rd., Gammaduwa, Sri Lanka; elevation 750m | partially disarticulated: lower jaws, 1pt, 1ept |
| M-10038 | F 348; CB 4 | *Rhinophis philippinus* | Gammaduwa Rd., Gammaduwa, Sri Lanka; elevation 750m | partially disarticulated: lower jaws, 1 q, pmx |
| M-10041 | F 387; CB 27 | *Rhinophis trevelyanus* | Balangoda (above), Samanala watta ['Swanalawatta' crossed out], Sri Lanka; elevation 950m (gift - Mr. Singha Bandora) | articulated: partial lower jaw removed |
| M-10045 | F 408; CB 29 | *Uropeltis melanogaster* | Lemastota, Nicopota, Sri Lanka; elevation 720m | fully disarticulated before preparation, 1 pt missing |
| M-10046 | F 434; CB 10 | *Rhinophis drummondhayi* | Hilagama on Bibilegama Rd., (near Namunukula), Wiwikeli Est., Sri Lanka; elevation 1020m | articulated |
