# Supplementary material for: Comparative Skull Morphology of Uropeltid Snakes (Alethinophidia: Uropeltidae) with Special Reference to Disarticulated Elements and Variation
Source: PLoS One. 2012 Mar 8;7(3):e32450. doi: 10.1371/journal.pone.0032450 (PMC3297617; doi:10.1371/journal.pone.0032450)
Supplement: Methods S1 — Character descriptions. (DOC) [file pone.0032450.s003.doc]

**Methods S1. Character descriptions.** Characters 1-33 correspond to characters of the same number published previously [17]. However, the descriptions of some of those previous characters are modified here as denoted by bold text.

1. Anterior tip of maxilla turned medially, closely approaching or touching transverse process of premaxilla (0); anterior tip of maxilla straight, contact with premaxilla shizarthrotic (1).

This character appears to be based on an earlier study of morphological characters in snakes (character 28 of [2]), and the states described in that work are referred to [75]. However, the term ‘shizarthrotic’ was not used by [75] and no definition was provided by subsequent authors. Three states were provided by [75] for the relationship between the maxilla and premaxilla, and the one that applied to uropeltids is a ‘sutural’ contact, in contrast to one with a clear gap between the elements, as well as an intermediate state [2]. Based on taxa that possess state 1 as given by [17], our use of ‘schizarthrotic’ refers to a tightly abutting sutural joint (Figs. 2,8).

2. Nasals relatively broad anteriorly, notched (0); nasals gradually tapering to pointed tip anteriorly (1).

This character is similar to character 4 used by [4]. The nasal in *U. woodmasoni* is distinctly emarginated in lateral view, but clearly tapers anteriorly (Fig. 12A). In dorsal view, the nasal in that taxon appears roughly triangular. In *B. rhodogaster* there is also a slight emargination laterally for articulation with the septomaxilla, but the dorsal lamina of the nasal remains relatively broad for almost its entire length anteriorly (best seen in dorsal view), and a distinct, small notch occurs in dorsal and lateral views (Fig. 12G,H). That morphology gives the nasal of *B. rhodogaster* a more rectangular shape in dorsal view.

3. Teeth on palatine present (0), absent (1).

This character is probably taken from character 18 of [2], itself based on [76]. All known uropeltids, except for *Melanophidium wynaudense* [17], lack teeth on the palatine (Fig. 21).

4. A distinct and well-defined buttressing contact between the **anteromedial process** of the maxilla and an **anterolateral** process of the vomer is absent (0), or present (1).

Description of this contact as a ‘well-defined buttress’ is insufficient. We maintain the original intent of Character 4 [17] of contact between the maxilla and vomer but note that in none of our material is the contact strong or ‘buttressed.’ The region of contact includes contributions from the maxilla, vomer, and premaxilla, and in some taxa (e.g. *Uropeltis rubromaculata*) there is a ventral projection of the septomaxilla that is visible in palatal view (Figs. 3,6,11,17-20). Character 4 can be worded as the presence of a contact between the maxilla and vomer; if they fail to meet, the septomaxilla is visible between them. In some individuals, the condition is bilaterally asymmetric.

5. Parietal enters optic foramen (0), optic foramen fully enclosed entirely within frontal (1).

**This character is similar to character 33 of [4], although in the latter, the description was put in terms of the morphology of the parietal border of the optic foramen. Character 5 appears straightforward but can be difficult to score in articulated specimens in which the foramen is fully enclosed within the frontal, but the parietal overlaps the frontal so as to contribute also to the lateral portion of the optic foramen. In those cases, the morphology was scored as state 1 (Fig. 14A,D), based on complementary disarticulated specimens. State 0 was used when the optic foramen was not fully enclosed in the frontal (i.e., the frontal formed a notch) and was completed by the parietal (Fig. 14H).**

6. **The ossified base of the** crista trabecularis ends behind the (lateral) **frontal-parietal** suture (0), at the (lateral) **frontal-parietal** suture (1), in front of the (lateral) **frontal-parietal** suture (2).

Note that in reference to the lateral frontal-parietal suture, the supraorbital process of the parietal is excluded (i.e., character should be scored from the ventral most expression of the lateral frontal-parietal suture, Fig. 11B). Also, ‘crista trabecularis’ refers specifically to only the **ossified base** of that structure, as reflected in our modification to the description.

7. Supraorbital process of parietal does not (0), or does (1) participate in suspension of prefrontal (contacts prefrontal above the orbit).

This character is based on character 21 of [4]. The term ‘suspension’ is inappropriate because even when the two processes abut one another, the articulation is loose and soft tissue provides all of the support (Figs. 2,8,9). Related to the presence of soft tissue, a high level of left-right asymmetry in individuals suggests strongly that differential drying in skeletal preparations is responsible for whether or not a ‘contact’ occurs.

8. Supraoccipital separate (0), or fused (1).

We interpret the term ‘fused’ here to mean that the supraoccipital is inseparable from other braincase elements and a suture is no longer visible. However, in uropeltids where the supraoccipital is absent (Figs. 5,6,23,24,27), it is unknown if the bone becomes fused with other elements or if it fails to develop entirely. No developmental studies exist. A more appropriate character description is whether or not a distinctly separate supraoccipital is present.

9. Prootic and opisthotic-exoccipital separate (0), or fused (1).

Based on the presence of sutures in some uropeltid taxa (see fig. 2.29, [55]), in other taxa in which separate elements are absent (Figs. 2,8,23,24,27), it appears that the bones have fused during development, although no developmental studies exist.

10. Basisphenoid – basioccipital separate (0), or fused (1).

See annotation for Character 9 and refer to figure 2.31 of [55] for illustration of state 0 in *Melanophidium wynaudense*. Examples of state 1 are found in Figures 4,7,23,24,27.

11. Laterosphenoid narrow (0) **(i.e., equal to or narrower than the V_3_ opening)**, broad (1) **(i.e., wider than the V_3_ opening)**.

We found that the width of the laterosphenoid varies gradationally and that many of our specimens fell into an intermediate category. As originally described the character was difficult to score so we amended the description to be more objective. State 0 refers to cases in which the laterosphenoid is equal to or narrower than the V_3_ opening (e.g., Fig. 8C), and state 1 refers to cases in which the laterosphenoid is wider than the V_3_ opening (e.g., Fig. 2C). Based on a high level of polymorphism in some taxa (e.g., *Uropeltis ceylanicus* from the British Museum of Natural History, pers. obs. JCO), variation in this character may be related to the absolute size of individuals, other aspects of individual variation, or ontogeny.

12. Facial nerve branches open into a recess behind the mandibular branch foramen which connects with the posterior opening of the Vidian canal (0); facial nerve branches open into a prootic canal which opens within the recession of the mandibular branch foramen and connects with the posterior opening of the Vidian canal (1); facial nerve branches open into a prootic canal which opens behind the mandibular branch foramen and connects with the posterior opening of the Vidian canal (2).

**This character appears to be a combination of Characters 67 and 68 of [4]. Their Character 67 referred to the position of the facial nerve foramen relative to only the opening for the mandibular branch of the facial nerve (V_3_), whereas character 68 referred to the position of the facial nerve foramen relative to only the opening for the Vidian canal. As currently written, Character 12 here is complex and unclear. We suggest simplifying the character to states in which the opening for the facial nerve is separate from (i.e. outside) the Vidian canal and separate from the opening for the V_3_ (0); is within a prootic canal and within the opening for the V_3_ (1); or within the prootic canal but separate from the opening for the V_3_ (2).**

13. Juxtastapedial recess wide open laterally (0; fenestra pseudorotunda may be exposed in lateral view), distinctly restricted by approximation of dorsal and ventral margin (1; fenestra pseudorotunda never exposed in lateral view). **New: state 2, restricted by approximation of dorsal and ventral margin (generally anteriorly or at the midpoint), but fenestra pseudorotunda visible in lateral view (i.e., the juxtastapedial recess is open posteriorly).**

Expression of this character varies gradationally across and within species of uropeltids. In some individuals the juxtastapedial recess is wide open with no restrictions (Fig. 27C), while in others the recess may be so restricted along the midpoint that it is nearly closed (Fig. 8C). The range of variation that we observed required the addition of a new state to accommodate an intermediate condition. Our new state 2 is based on the observation that the restriction or pinching of the juxtastapedial recess usually occurs at the anterior half of the recess so that in many cases the posterior portion remains wide open (Fig. 23C). The problem with the character states as originally described is that the lateral exposure of the foramen is compared with the restriction of the juxtastapedial recess, which does not accommodate all states. Future work on variation across a broader range of taxa and morphologies will likely necessitate more drastic redescription or modification of this character.

14. Jugular foramen behind juxtastapedial recess **[and crista circumfenestralis]** (0), recessed within **~~juxtastapedial recess~~ [crista circumfenestralis but separated from the juxtastapedial recess by wall of bone]** (1). **New state: in well defined ‘cup’ posterior to juxtastapedial recess, but still bound (recessed within) by crista circumfenestralis (2).**

Previously, the condition in *U. woodmasoni* was scored as being within the juxtastapedial recess, whereas the condition in *B. rhodogaster* was scored as being outside [17]. However, in our specimens, the conditions appear to be comparable, but *U. woodmasoni* has a bigger, deeper ‘cup’ structure surrounding the jugular foramen. We saw no uropeltid taxa in which the foramen is recessed within the juxtastapedial recess. In all the uropeltid specimens examined, the foramen was within the crista circumfenestralis, but it was always separated from the recess by wall of bone of varying thickness. In some taxa (e.g., *U. woodmasoni*), the area around the foramen forms a distinct round, laterally swollen cup (Figs. 23C,26). In *B. rhodogaster*, the margin of the crista circumfenestralis forms a distinct ridge bounding the foramen, but the area is not swollen into a laterally expanded cup (Fig. 27C). In *U. woodmasoni*, the wall is narrow, less than or equal to the width of the foramen; in *B. rhodogaster* and the *Rhinophis* species we examined, the wall is broad and wider than the foramen. Both conditions differ from that in *Cylindrophis* in which the exoccipital forms a long tube leading medially to the jugular foramen. However, even in that case the foramen is still separated from juxtastapedial recess by a narrow wall of bone (much narrower than the foramen).

15. Jugular foramen internally subdivided (0), single (1).

This character is sometimes asymmetric on the left and right sides of the skull. In other cases, the bifurcation may occur deeply and is therefore difficult to score in articulated and/or poorly prepared specimens. For example, in TMM M-10001 (*U. woodmasoni*), the opening is divided at the lateral surface on the left side, but on the right the bifurcation appears to be deeper and smaller. In our CT-scanned specimen (TMM M-10006), the opening appears single when examined by eye, but scans reveal that the opening is single laterally (i.e., near the external surface) and then bifurcates internally, deep within the skull, before opening into the endocranial cavity. Figure 26 shows a case where the opening clearly is subdivided at the external surface.

16. More than one hypoglossal foramina (0), single **~~but enlarged~~**

hypoglossal foramen (1).

In our sample, we found that the foramen can be single but not enlarged (e.g., *U. woodmasoni* and *U. melanogaster*). Also, the number of openings appears to be subject to a high level of individual variation. This character may need to be redescribed or modified to encompass a wider range of variation.

17. Stalk of occipital condyle short, depression in basioccipital for brainstem not visible in dorsal view (0), stalk of occipital condyle elongate, depression in basioccipital for brainstem visible in dorsal view (1).

In taxa that posses state 0 (e.g. *B. rhodogaster*, Figs. 5C,27A), the depression for the brainstem may be partially visible, but it is not as obvious or well-devloped as the elongated, deep triangular depression observed in taxa that exhibit state 1 (e.g. *U. woodmasoni*, Figs. 5A,23A). Also, in taxa that posses state 0, the depression meets the ball of the occipital condyle whereas in long-stemmed taxa the depression terminates anterior to the ball of the condyle.

18. Posteroventral process of dentary distinct (0), reduced (1), absent (2).

The posteroventral process of the dentary is absent in most known uropeltids (Fig. 28). The process is distinct, but small, in *Melanophidium* (fig. 2.33E,F [55]), and reduced in *Platyplectrurus madurensis* (fig. 2.33C,D [55]). The process is absent in *Plectrurus perroteti* (fig. 2.33A,B [55]; BMNH 1930.5.8.106, pers. obs. JCO), although previously that taxon was reported to posses state 1 [17].

19. Exoccipitals not in contact dorsal to basioccipital in occipital condyle (0); exoccipitals in contact dorsal to basioccipital in occipital condyle (1); exoccipitals and basioccipital fused in occipital condyle (2).

State 2 of this character is based on the assumption that the exoccipitals and basioccipitals fuse during cranial development. In some taxa (e.g., *Melanophidium*, fig. 2.31 [55]) the bones are separate, while in others (e.g., *U. woodmasoni*, Figs. 2,23C) there are no visible sutures in that region of the braincase, but no developmental data exist (see Characters 8,9,10 above).

20. Anterior **~~dentigerous~~ vomerine** process of palatine slender and straight (0), broadened anteriorly (1), modified into expanded lamina (2).

Most uropeltid taxa that have been described lack palatine teeth (Fig. 21). In those cases the term ‘dentigerous process’ is misleading. We recommend adoption of ‘vomerine process of palatine’ because it is applicable to a broader taxonomic sample of snakes and is independent of whether or not teeth occur.

21. Pterygoid teeth present (0), absent (1)

In [17], Character 50 of [4] was cited as the origin of this character. Character 21 is also similar to Character 23 of [2], itself based on descriptions by [76]. All known uropeltids lack pterygoid teeth (Fig. 22).

22. Suprastapedial process of **~~stapes~~** **quadrate** is not (0), or is (1) distinctly longer than shaft of **~~stapes~~ quadrate**.

In the original character description [17], Character 57 of [4] was cited. From the latter, as well as reference to the ‘surprastapedial process,’ found only on the quadrate, it is clear that this character should reference the ‘quadrate’ rather than the stapes (confirmed by O. Rieppel, pers. com. CJB). All known uropeltids share state 1 (Fig. 25).

23. Quadrate suspension close to dorsal margin of otic capsule (0), shifted anteroventrally on otic capsule (1).

In the original character description [17], Character 58 of [4] was cited. Similarities are also shared with Character 27 of [2], which described the position of the proximal articulation of the quadrate relative to sutures in the braincase. All known uropeltids share state 1 (Figs. 2,8).

24. Retroarticular process unmodified (0), wrapping around posterior aspect of mandibular condyle of quadrate (1).

Character 32 of [2] was cited as the basis for this character [17]. However, the character is also similar to Character 89 of [4], and both [2] and [4] described the modifications referred to here as either a short process with a dorsal (wrapping) spatulate process, or an elongate process. The wrapping condition (state 1, Fig. 28) is extreme in some *B. rhodogaster* specimens in which the retroarticular process curves anteriorly to nearly contact the anterior rim of the cotyle for the mandibular condyle. Relative to conspecifics, the retroarticular process is less strongly wrapped in the small *U. melanogaster* specimen and some individuals of other *Uropeltis* species suggesting this morphological variation may be the result of ontogenetic transformation. However, even in those cases the retroarticular process still extends dorsally, and is modified relative to the condition in *Cylindrophis* in which the cotyle is open posteriorly.

25. Premaxillary teeth present (0), absent (1).

All known uropeltids lack premaxillary teeth (Fig. 1).

26. **In ventral view,** contact between premaxilla and vomer overlapping (0), or in well defined recess (1).

As currently written, this character is insufficient for complex articulations that include overlapping *and* abutting contacts. In those cases, which are represented by all uropeltid species examined by us, we scored taxa as having state 1 in order to retain the presumed intent of [17]. However, the meaning of ‘recess’ is unclear, although we interpreted it to mean the ‘step’ (in ventral view) from the flat, anteriormost ventral palatal surface of the premaxilla to the higher (more dorsal) articulation area of that element. In *Cylindrophis rufus* the articulation is clearly overlapping and should be scored the same as in *Anilius*, in contrast to previous reports [17]. Future work addressing variation among a broader sample of taxa may necessitate modification of this character to states describing a purely clasping articulation, a purely abutting articulation, or a mixed, complex articulation similar to the ones present in the *Brachyophidium*, *Uropeltis*, and *Rhinophis* species that we described (Figs. 4,7,11,16,17-20).

27. Preorbital ridge on frontal [60] does not (0), does (1) project beyond anterior margin of dorsally exposed surface of frontal.

This character is straightforward to score, but the condition can be observed only in disarticulated material, ct scans, or other special preparations. All known uropeltids share state 1 (Fig. 14).

28. Interchoanal process of parasphenoid absent (0), present (1).

This character is based on Character 17 of [2] and refers to an elaboration of the surface of the parasphenoid ventral to the cultriform process that takes the form of an anteroventral projection. Among uropeltids, state 0 (absent) is exemplified by *B. rhodogaster* (Fig. 27). State 1 is exemplified by *U. woodmasoni* in which the process is a sharp, triangular keel (Fig. 23). A sharp keel also was illustrated in *Melanophidium wynaudense*, whereas the process was illustrated as more rounded and hook-like in *Anomochilus weberi* (figs. 2.20B, 2.31B, [55]). The process is present and needle-like in *Cylindrophis rufus* (UCMP 136995, pers. obs., JCO). CT scans of *Anilius* also reveal the presence of the process in contrast to previously reported absence in that taxon [17]. This character may have a wider distribution and more varied conditions than are currently known.

29. Posteroventral part of crista circumfenestralis does not (0), or does (1) form a distinctly enlarged gliding surface for the quadrate ramus of the pterygoid, or this gliding surface is present but “rounded off” (2).

Based on the current description, it is unclear if the region described is homologous with the sphenoccipital tubercle of *Cylindrophis* and *Anilius* because it is unclear if it is ventral to the sutural area between the basioccipital and exoccipital or if it is a swelling of the crista circumfenestralis. This character is difficult to score objectively, particularly in small specimens that may lack the more developed features of larger individuals or taxa. Particularly, the description ‘rounded off’ is ambiguous. In all specimens of *Branchyophidium*, *Uropeltis*, and *Rhinophis* described by us, that region of the braincase is smooth, with no distinct bump. However, in the much larger *Platyplectrurus madurensis*, there is a distinct, almost squared structure posterior to the end of the pterygoid (pers. obs., JCO).

30. Occipital condyle is not (0), or is (1) modified as described by [52] and [54].

All known uropeltids exhibit state 1.

31. The superior alveolar nerve canal in the maxilla is closed (0), or open (1) dorsally.

This character is straightforward to score, but requires clean, disarticulated material, CT scans, or other special preparations. All known uropeltids share state 1 (Fig. 3).

32. **Single frontal bone** is not (0), is (1) at least twice as long as broad.

The original character description [17] was ambiguous as to whether the measurements are to be taken on the paired frontals or a single frontal bone. The description was modified to follow original authors’ intent that the measurements refer to a single frontal bone (confirmed by O. Rieppel, pers. com. CJB). The term ‘broad’ refers to the width of a single frontal bone at its widest point (Fig. 14).

33. Supratemporal present (0), absent (1).

This character is the same as Character 38 of [4], and Character 16 of [2]. Controversy exists about whether the uropeltid supratemporal is lost (i.e., fails to form), incorporated into the braincase wall (i.e., fused, with no visible sutures), or reduced (i.e., present) but overlooked in dried skulls from which it may drop off during preparation [55,77].
