# Supplementary material for: Comparative Skull Morphology of Uropeltid Snakes (Alethinophidia: Uropeltidae) with Special Reference to Disarticulated Elements and Variation
Source: PLoS One. 2012 Mar 8;7(3):e32450. doi: 10.1371/journal.pone.0032450 (PMC3297617; doi:10.1371/journal.pone.0032450)
Supplement: Methods S2 — Character matrix for phylogenetic analysis of morphological characters in PAUP*. (DOC) [file pone.0032450.s004.doc]

**Methods S2. Character matrix for phylogenetic analysis of morphological characters in PAUP*. See Methods S1 for Character Descriptions. Methods S3 is matrix in Nexus format.** Bold text indicates scoring changes and additions to original matrix published by [17].

|  | **1** | **2** | **3** | **4** | **5** | **6** | **7** | **8** | **9** | **10** | **11** | **12** | **13** | **14** | **15** | **16** | **17** |
| --- | --- | --- | --- | --- | --- | --- | --- | --- | --- | --- | --- | --- | --- | --- | --- | --- | --- |
| *Anilius* | 0 | 0 | 0 | 0 | 0 | 0 | 0 | 0 | 0 | 0 | 0 | 0 | 0 | 0 | 0 | 0 | 0 |
| *Cylindrophis* | 0 | 0 | 0 | 0 | 0 | 0 | 0&1 | 0 | 0 | 0 | 0 | 0 | 0 | 0 | 0 | 0 | 0 |
| *Anomochilus* | 0 | 0 | 1 | 0 | 0 | 0 | 1 | 0 | 0 | 0 | 0&1 | 0 | 0 | 0 | ? | 0 | 0 |
| *Melanophidium punctatum* BMNH 1930.5.8.119 | 0 | **1** | 0 | 0 | 0 | 0 | 1 | 0 | 0 | 0 | 0 | 0 | 0 | 0 | 0 | **0&1** | 0 |
| *Melanophidium wynaudensis* BMNH 1930.5.8.124–125 | 0 | 0 | 1 | 0 | 0 | 0 | 0&1 | 0 | 0 | 0 | 0 | 0 | 0 | 0 | 0 | 1 | 0 |
| *Platyplecturus madurensis* BMNH 1930.5.8.111 | 0 | **0** | 1 | 0 | 0 | 0 | 0&1 | 1 | 1 | 1 | **0** | 2 | **2** | **2** | 1 | 1 | 0 |
| *Uropeltis woodmasoni* BMNH 1930.5.8.73–74 | 1 | 1 | 1 | 1 | 1 | 2 | 0 | 1 | 1 | 1 | 1 | 2 | 1 | **2** | 0 | 1 | 1 |
| *Brachyophidium rhodogaster* BMNH 1930.5.8.98 | 1 | 0 | 1 | 0 | 0 | 1 | 1 | 1 | 1 | 0 | 1 | 2 | 0 | **1** | 1 | 1 | 0 |
| *Rhinophis drummondhayi* BMNH 1930.5.8.67–68 [b] | 1 | 1 | 1 | 1 | 1 | 1 | 1 | 1 | 1 | 1 | 1 | 2 | 1 | 1 | 0 | 1 | 1 |
| *Rhinophis sanguineus* BMNH 1930.5.8.59 | 1 | 1 | 1 | 1 | 1 | 2 | 0 | 1 | 1 | 1 | 1 | 1 | 1 | 1 | 0 | 1 | 1 |
| *Plectrurus perroteti* BMNH 1930.5.8.105 | 1 | 1 | 1 | 0 | 1 | 0 | 0&1 | 1 | 1 | 1 | 1 | 2 | 1 | 1 | 0 | 1 | 1 |
| *Pseudotyphlops philippinus* BMNH 1978.1092 | 1 | 1 | 1 | 0 | 0 | 0 | 1 | 1 | 1 | 1 | 0 | 1 | 1 | 0 | 1 | 1 | 1 |
| *Plectrurus aureus* CAS 17177 | 1 | 1 | 1 | 0 | 1 | 0 | 0 | 1 | 1 | 1 | 1 | 2 | 0 | 1 | 0 | 1 | 1 |
| *Uropeltis woodmasoni* | 1 | 1 | 1 | 1&0 | 1 | 1&2 | 0&1 | 1 | 1 | 1 | 1 | 2 | 0&1&2 | 2 | 1&0 | 1 | 1 |
| *Brachyophidium rhodogaster* | 1 | 0 | 1 | 1&0 | 0 | 0&1&2 | 1&0 | 1 | 1 | 1 | 0&1 | 2 | 0 | 1 | 1 | 1&0 | 0 |
| *Uropeltis rubramaculata*TMM M-10028 | 1 | 1 | 1 | 0 | 1 | 0 | 0 | 1 | 1 | 1 | 1 | 2 | 1 | 2 | 0&1 | 1 | 1 |
| *Uropeltis melanogaster* TMM M-10045 | 1 | 1 | 1 | ? | 1 | ? | ? | 1 | 1 | 1 | 0 | 1&2 | 2 | 1 | 1 | 1 | 1 |
| *Uropeltis melanogaster* TMM M-10032 | 1 | 1 | 1 | ? | 1 | ? | ? | 1 | 1 | 1 | 0/1 | 2 | 0 | 1 | 1 | 1 | 1 |
| *Rhinophis blythii*TMM M-10030 | 1 | 1 | 1 | 0&1 | 1 | 0 | 0 | 1 | 1 | 1 | 1 | ? | 0 | 2 | 1 | 1 | 1 |
| *Uropeltis* sp. TMM M-10036 | 1 | 1 | 1 | ? | 1 | 0 | 0 | 1 | 1 | 1 | 0 | 2 | 0 | 1 | 1 | 1 | 0 |
| *Rhinophis philippinus*? TMM M-10037 | 1 | 1 | 1 | 1 | 1 | 2 | 1 | 1 | 1 | 1 | 0 | 2 | 1 | 2 | 1 | 1 | 1 |
| *Rhinophis philippinus* TMM M-10038 | 1 | 1 | 1 | ? | 1 | 2 | 1 | 1 | 1 | 1 | 0 | 2 | 2 | 2 | 1 | 0 | 1 |
| *Rhinophis homolepis* TMM M-10041 | 1 | 1 | 1 | 1 | 1 | 1 | 1 | 1 | 1 | 1 | 1 | 2 | 2 | 1 | 1 | 1 | 1 |
| *Rhinophis drummondhayi* TMM M-10046 | 1 | 1 | 1 | 1 | 1 | 2 | 1 | 1 | 1 | 1 | 1 | 2 | 0 | 1 | 1 | 1 | 1 |

Methods S2 continued

|  | **18** | **19** | **20** | **21** | **22** | **23** | **24** | **25** | **26** | **27** | **28** | **29** | **30** | **31** | **32** | **33** |
| --- | --- | --- | --- | --- | --- | --- | --- | --- | --- | --- | --- | --- | --- | --- | --- | --- |
| *Anilius* | 0 | 0 | 0 | 0 | 0 | 0 | 0 | 0 | 0 | 0 | **1** | 1 | 0 | 0 | 0 | 0 |
| *Cylindrophis* | 0 | 1 | 0 | 0 | 0 | 0 | 0 | 1 | **0** | 1 | 1 | 1 | 0 | 0 | 0 | 0 |
| *Anomochilus* | 0 | 1 | 1 | 1 | 1 | 1 | 1 | 1 | 1 | 1 | 1 | 2 | 0 | 0 | 0 | 0 |
| *Melanophidium punctatum* BMNH 1930.5.8.119 | 0 | 2 | 2 | 1 | 1 | 1 | 1 | 1 | 1 | **?** | 1 | 1 | 1 | 1 | 1 | 1 |
| *Melanophidium wynaudensis* BMNH 1930.5.8.124–125 | 0 | 2 | 2 | 1 | 1 | 1 | 1 | 1 | 1 | 1 | 1 | 1 | 1 | 1 | 1 | 1 |
| *Platyplecturus madurensis* BMNH 1930.5.8.111 | 1 | 2 | 2 | 1 | 1 | 1 | 1 | 1 | 1 | **?** | 1 | 2 | 1 | **?** | 1 | 1 |
| *Uropeltis woodmasoni* BMNH 1930.5.8.73–74 | 2 | 2 | 2 | 1 | 1 | 1 | 1 | 1 | 1 | 1 | 1 | 2 | 1 | 1 | 1 | 1 |
| *Brachyophidium rhodogaster* BMNH 1930.5.8.98 | 2 | 2 | 2 | 1 | 1 | 1 | 1 | 1 | 1 | 1 | 1 | 2 | 1 | 1 | 1 | 1 |
| *Rhinophis drummondhayi* BMNH 1930.5.8.67–68 [b] | 2 | 2 | 2 | 1 | 1 | 1 | 1 | 1 | 1 | 1 | 1 | 2 | 1 | 1 | 1 | 1 |
| *Rhinophis sanguineus* BMNH 1930.5.8.59 | ? | 2 | 2 | 1 | 1 | 1 | 1 | 1 | 1 | 1 | 1 | 2 | 1 | 1 | 1 | 1 |
| *Plectrurus perroteti* BMNH 1930.5.8.105 | 1 | 2 | 2 | 1 | 1 | 1 | 1 | 1 | 1 | 1 | 1 | 2 | 1 | 1 | 1 | 1 |
| *Pseudotyphlops philippinus* BMNH 1978.1092 | 2 | 2 | 2 | 1 | 1 | 1 | 1 | 1 | 1 | 1 | 1 | 2 | 1 | 1 | 1 | 1 |
| *Plectrurus aureus* CAS 17177 | 2 | 2 | 2 | 1 | 1 | 1 | 1 | 1 | 1 | 1 | 1 | 2 | 1 | 1 | 1 | 1 |
| *Uropeltis woodmasoni* | 2 | 2 | 2 | 1 | 1 | 1 | 1 | 1 | 1 | 1 | 1 | 2 | 1 | 1 | 1 | 1 |
| *Brachyophidium rhodogaster* | 2 | 2 | 2 | 1 | 1 | 1 | 1 | 1 | 1 | ? | 0 | 2 | 1 | 1 | 1 | 1 |
| *Uropeltis rubramaculata*TMM M-10028 | 2 | 2 | 2 | 1 | 1 | 1 | 1 | 1 | 1 | ? | 1 | 2 | 1 | ? | 1 | 1 |
| *Uropeltis melanogaster* TMM M-10045 | 2 | 2 | 2 | 1 | 1 | ? | 1 | 1 | ? | 1 | 1 | 2 | 1 | 1 | 1 | 1 |
| *Uropeltis melanogaster* TMM M-10032 | 2 | 2 | 2 | 1 | 1 | ? | 1 | 1 | ? | 1 | 1 | ? | 1 | 1 | 1 | 1 |
| *Rhinophis blythii*TMM M-10030 | 2 | 2 | 2 | 1 | 1 | 1 | 1 | 1 | 1 | ? | 1 | 2 | 1 | ? | 1 | 1 |
| *Uropeltis* sp. TMM M-10036 | 2 | 2 | 2 | 1 | 1 | 1 | 1 | 1 | 1 | ? | 1 | 2 | 1 | 1 | 1 | 1 |
| *Rhinophis philippinus*? TMM M-10037 | 2 | 2 | 2 | 1 | 1 | 1 | 1 | 1 | 1 | ? | 1 | 2 | 1 | 1 | 1 | 1 |
| *Rhinophis philippinus* TMM M-10038 | 2 | 2 | 2 | 1 | 1 | 1 | 1 | 1 | ? | ? | 1 | 2 | 1 | 1 | 1 | 1 |
| *Rhinophis homolepis* TMM M-10041 | 2 | 2 | 2 | 1 | 1 | 1 | 1 | 1 | 1 | ? | ? | 2 | 1 | ? | 1 | 1 |
| *Rhinophis drummondhayi* TMM M-10046 | 2 | 2 | 2 | 1 | 1 | 1 | 1 | 1 | 1 | ? | ? | 2 | 1 | ? | 1 | 1 |
