# Supplementary material for: Comparative Skull Morphology of Uropeltid Snakes (Alethinophidia: Uropeltidae) with Special Reference to Disarticulated Elements and Variation
Source: PLoS One. 2012 Mar 8;7(3):e32450. doi: 10.1371/journal.pone.0032450 (PMC3297617; doi:10.1371/journal.pone.0032450)
Supplement: Methods S4 — Descriptions of potential new characters. (DOC) [file pone.0032450.s006.doc]

**Methods S4. Descriptions of potential new characters.** Whenever possible the distribution of preliminary character states is provided in the annotations for the specimens from the TMM collection. A summary table is not provided because all potential characters in this list require further testing and modification through the examination of a larger number of individuals and a broader taxonomic sample. None of the described states imply ordered characters.

1. Anterior tip of the premaxilla narrow and pointed (0), or inflated into distinctly expanded rostrum with a bifurcated tip (1).

In our sample, state 0 is exemplified in *B. rhodogaster*, whereas state 1 occurs in all species of *Uropeltis* and *Rhinophis* we examined (Fig. 1).

2. A mediolaterally oriented canal penetrating the septum at the base of the nasal process is absent (0), or present (1).

Within our sample, only specimens of *B. rhodogaster* lack a canal piercing the septum at the base of the nasal process. All other taxa that we surveyed exhibit that canal (Fig. 1). In some individuals of *U. woodmasoni*, however, the canal is incomplete on one side or may be absent.

3. Ventral premaxillary foramen (see text) in ventral surface of premaxilla is single (0), or multiple (1).

With clean specimens, multiple openings often are visible in addition to the single large opening described previously in some uropeltids [17]. A single opening is present in *U. woodmasoni*, *R. philippinus*, *R. homolepis*, and *B. rhodogaster*. The remaining taxa that we sampled possess multiple foramina.

4. Subnarial fenestra (sometimes foramen, see text) between premaxilla and palate absent (0), or present (1).

Clean, articulated specimens are required to score this feature. Among our specimens we observed state 1 in all species of *Rhinophis* and *Uropeltis*. State 0 is present in *B. rhodogaster* (Figs. 4,7,11,17-20).

5. Canal located along the lateral wall of the nasal process of premaxilla absent (0), or present (1).

This feature can be scored only in clean, disarticulated material. We observed state 1 in disarticulated individuals of *R. blythii* and *U. melanogaster*. State 0 was observed in *U. woodmasoni* and *B. rhodogaster*. The condition could not be scored in the other taxa.

6. In articulated skulls, anterior projections of the premaxillary process of the vomer that bound the vomerine process of the premaxilla are absent (0), or present (1).

This feature should be scored using clean, articulated material. Both specimens of *U. melanogaster* were disarticulated and therefore could not be scored. We observed the anterior projections in *R. blythii*, *R. philippinus*, and *R. homolepis*. The projections were absent in *U. woodmasoni* and *U. rubromaculata.* When partially disarticulated material is available for a broader sample of taxa, this feature may need to be modified to reflect the underlying morphology of the vomer as it affects the shape of the contact in external view. In articulated material it appears that the anterior projections are slivers of vomer visible within the subnarial fenestrae (foramina). In taxa that exhibit state 0, the morphology of the vomer is hypothesized to be such that the anterolateral extensions of the bone are absent or obscured relative to those that exhibit state 1. The most appropriate score for the condition in *B. rhodogaster* may be ‘non applicable’ because the nature of the contact between the vomer and premaxilla differs from that of the other taxa we examined (refer to text and Fig. 4).

7. The posteriormost tooth position on the maxilla is located anterior to the anteriormost contact with the ectopterygoid (0), at the anteriormost contact with the ectopterygoid (1), or posterior to the anteriormost contact with the ectopterygoid (2).

Even when the teeth have been lost, the position of the posteriormost tooth socket is easily identified when the specimen remains articulated. There is ambiguity when specimens are disarticulated, although the contact between the maxilla and ectopterygoid is elongate and often leaves a roughened scar on the maxilla. We observed state 0 in specimens of *B. rhodogaster* and *R. homolepis*; state 1 in *R. blythii*, *R. drummondhayi*, and *R. philippinus*; and state 2 in *U. woodmasoni*. The condition in *U. melanogaster* tentatively is reported to be state 2, although both specimens were disarticulated.

8. Minimum number (taking into account left-right asymmetries and intraspecific variation) of maxillary teeth are six or more (0), or five or fewer.

Before characters based on maxillary tooth morphology can be better refined, a broader taxonomic sample of dentition is required. There appear to be two broad patterns within uropeltids; many taxa (e.g., *U. woodmasoni*, *U. melanogaster*, species of *Rhinophis*, *B. rhodogaster*) have a few, large teeth (minimum of five or fewer, taking individual variation into account), whereas others (e.g., *U. rubromaculata*) have a higher number of smaller teeth (minimum of six or more). However, in *R. philippinus*, the teeth are relatively small, but only five are present. At least two characters relating to the teeth should be described, but we chose not to attempt description of character states for size until more taxa are examined.

9. The position of the posteriormost maxillary foramen is anterior to the midpoint of the ascending process of the maxilla (0), centered ventral to the ascending process (1), or posterior to the midpoint of the ascending process (2).

In all species of *Rhinophis* that we examined, the foramen is located anteriorly (state 0). In *R. drummondhayi* the foramen is entirely anterior to the full extent of the ascending process. Our specimen of *U. rubromaculata* exhibits state 1, and multiple states occur in our sample of *B. rhodogaster*.

10. The position of the anteriormost maxillary foramen is located at the level of the posterior half of the anteromedial process of the maxilla (0), posterior to the anteromedial process (1), or anterior to the anteromedial process (2).

Clean, disarticulated material is required to score this feature. In our sample, state 0 occurs in *U. woodmasoni* and *U. rubromaculata*; state 1 occurs in *U. melanogaster*, *R. blythii*, *R. drummondhayi, R. philippinus*, and *R. homolepis*; and state 2 occurs only in *B. rhodogaster*.

11. The anterior surface of the anteromedial process of the maxilla does (0) or does not (1) articulate with the premaxilla.

Articulated specimens are necessary to score this feature. Because of that limitation, *U. melanogaster* could not be scored. Out of the remaining sample available to us, only *B. rhodogaster* exhibits state (0).

12. A premaxillary process of the septomaxilla is absent (0) or present (1).

This feature is most easily scored from disarticulated septomaxillae (Fig. 10), although when present, the triangular premaxillary process is visible inside the nasal passage of clean, articulated specimens (Fig. 2). The process occurs in all specimens of *Uropeltis* and *Rhinophis* that we examined, but is absent in *B. rhodogaster*.

13. The posteromedial process of the septomaxilla is short (shorter than the diameter of the vomeronasal foramen) (0), or long (longer than the diameter of the vomeronasal foramen) (1).

Specimens must be fully or partially disarticulated to score this feature. Our specimens of *B. rhodogaster* exemplify state 0, whereas state 1 occurs in specimens of *U. woodmasoni* and *U. melanogaster* (Fig.10). The other taxa could not be scored because no disarticulated material was available. Although the condition in *B. rhodogaster* is markedly different from that of the other taxa we scored, including a broader sample of taxa will necessitate refinement of the descriptions ‘short’ verses ‘long’ to accommodate intermediate states.

14. The medial wall of the nasal is smooth (0), marked by a transverse groove (1), or exhibits an anterior canal (2).

Refer to the text for detailed descriptions of states 1 and 2. Disarticulated material is required to score this feature. We observed state 0 in specimens of *B. rhodogaster*, state 1 in *U. melanogaster* and *R. philippinus*, and state 2 in *U. woodmasoni*. The other taxa could not be scored.

15. Lateral tubercle on nasal absent (0) or present (1).

Disarticulated specimens are necessary to score this feature. The tubercle is absent in *B. rhodogaster*, *R. philippinus*, and *U. melanogaster*, but is present in *U. woodmasoni*. The other taxa could not be scored.

16. The frontal process of the prefrontal is equal to (0) or longer than (1) the lateral foot process of the prefrontal.

In our sample, all taxa exhibit state 1 except for *B. rhodogaster*, which exhibits state 0.

17. The height of the prefrontal is less than or equal to the length of bone (0), or greater than the length (1).

In lateral view, the shape of the prefrontal varies among taxa. In some (e.g., *U. woodmasoni*) it may appear almost diamond-shaped, whereas in other taxa the angles of the element are more rounded (Fig. 13). One contributor to the overall shape is the difference between the length and the height of the prefrontal. In *U. melanogaster* and *R. blythii*, the height exceeds the length (state 1), but in all other taxa we examined the height is less than or equal to the length (state 0). *R. drummondhayi* and *R. philippinus* may exhibit a more intermediate morphology, but more taxon sampling is required to refine this feature. An additional contributor to the shape of the prefrontal is the degree of angularity in the anterior corner. In *U. woodmasoni* the corner is prominent, lending an overall diamond-shape to the element, whereas in *U. melanogaster* the corner appears to be absent (Fig. 13). More taxa need to be surveyed, but the morphology of the anterior margin may be phylogenetically informative as a separate feature.

18. Bifurcation of the groove traversing the ventromedial surface of the frontal is absent (0) or present (1).

Clean, disarticulated or partially disarticulated material is required to score this feature. Among the taxa we examined bifurcation is present only in *U. woodmasoni*. It is absent in *B. rhodogaster*, *R. philippinus*, and *U. melanogaster*. The other taxa could not be scored.

19. The sagittal crest terminates anteriorly at a shallow depression (0), roughened circular patch (1), or triangular flat spot that tapers posteriorly (2).

In all taxa that we examined the sagittal crest is only weakly developed compared to non-uropeltid snakes and larger species described previously [55]. In taxa in which the crest terminates anteriorly at a shallow depression (*U. woodmasoni*, *U. rubromaculata*, *R. blythii*, *R. drummondhayi*, *R. philippinus*, *B. rhodogaster*), most specimens also exhibit a thickened anterior rim delineating that depression. In *U. melanogaster* the sagittal crest bifurcates slightly as it terminates anteriorly and a triangular, smooth, flat spot originates between the bifurcation. In *U. rubromaculata* the crest ends anteriorly at a roughened, circular patch of bone. Our specimen of *R. homolepis* lacks a sagittal crest and could not be scored. The lack of crest in that specimen may be associated with small size and/or ontogeny.

20. A tab-like process of the parietal for articulation with the braincase is absent (0) or present (1).

Disarticulated material is required to score this feature. *Uropeltis woodmasoni* exemplifies state 1, whereas specimens of *B. rhodogaster* and *U. melanogaster* exhibit state 0 (Fig. 15). The other taxa could not be scored.

21. Tab of bone projecting into the vomeronasal opening originates at the ventral surface of the vomer (0) or dorsal to the ventral surface of the vomer (1).

In our sample, *R. drummondhayi* and *R. philippinus* exhibit state 1, whereas all other taxa we examined exhibit state 0 (Fig. 16).

22. The palatine process of the vomer is shorter than (0), or as long as or longer than (1) the posterior extent of the interchoanal process of the palatine.

In taxa that exhibit state 0 (Fig. 16D) the posterior margin of the body of the vomer terminates as short, triangular tip and does not conceal the interchoanal process of palatine. State 0 is exemplified by *U. melanogaster* and the species of *Rhinophis* that we examined. In taxa that exhibit state 1 (Fig. 16A), an elongate, narrow palatine process extends posteriorly away from the body of the vomer, and can be as long as, or longer than, the interchoanal process of the palatine so that the latter is obscured in ventral view. State 1 is exemplified by *U. woodmasoni* and also found in *U. rubromaculata* and *B. rhodogaster*. In *Anomochilus*, the posterior process of the vomer is located more laterally than in uropeltids and therefore does not obscure the interchoanal process of the palatine, even though the two processes are the same length.

23. Lateral process of the palatine formed by a closed loop of bone (0), or the overlying loop is not closed and the process remains separate (1).

In the all of the taxa that we examined except for *R. blythii*, in dorsolateral view of articulated skulls, and especially in disarticulated material, the lateral ‘process’ of the palatine is actually the ventral surface of a loop of bone surrounding the large foramen for CN V_2_ (state 0; Fig. 21B). In *R. blythii*, however, there appears to be a true separate lateral process of the palatine because the overlying loop is not closed (state 1).

24. Number of small foramina in the lateral surface of the palatine is one (0), two (1), or zero (2).

Intraspecific variation for this feature was relatively high even within our small sample. Broader taxonomic sampling may reveal that the level of variation is too high to support the utility of this feature. We observed a single foramen in *U. woodmasoni*, *U. melanogaster*, and *R. drummondhayi*. Two foramina are present in *U. rubromaculata* and *R. blythii*. No openings are present in the lateral surface of *R. homolepis*. One individual of *R. philippinus* exhibits one opening, whereas the second specimen has none. Most specimens of *B. rhodogaster* exhibit one opening, although some individuals have none and others have two.

25. The anterior margin of the vomerine process of the palatine is broadly rounded (0) or triangular (1).

In *R. drummondhayi*, the anterior margin of the vomerine process of the palatine is triangular. In all other taxa that we examined, the anterior margin is more broadly rounded. However, a few of our specimens exhibited conditions that are somewhat intermediate and the number of intermediate morphologies likely will increase as more taxa are sampled.

26. At the location of the bend (angle) near the midpoint of the ectopterygoid, a triangular flange is absent (0) or present (1).

Our specimen of *U. rubromaculata* exhibits state 1. All other taxa that we examined exhibit state 0 with the exception of *B. rhodogaster*, in which different individuals exhibited either state 1 or 0.

27. Concave arch in the dorsal margin of the caudal process of the quadrate is absent (0), or present (1).

State 1 is exhibited only by our specimens of *B. rhodogaster* (Fig. 25). All other taxa that we examined exhibit state 0.

28. In anterior view the groove for Meckel's cartilage is not (0) or is visible (1).

Among our specimens, only *R. blythii* exhibits state 1. In that taxon, the visibility of the groove produces a medial, trochlea-like expansion of the anterior tip of the dentary. All other taxa we examined exhibit state 0.

29. The posteriormost tooth on the dentary is positioned at the level of the angular-splenial contact (0), posterior to the angular-splenial contact (1), or anterior to the angular-splenial contact (2).

Within our sample, only *U. rubromaculata* exhibits state 1, and only *R. homolepis* and *B. rhodogaster* exhibit state 2. All remaining taxa that we examined exhibit state 0.

30. Teeth on the dentary not enlarged (0), or enlarged (1).

As currently written, this description is subjective because it is based primarily on comparisons with the condition in *U. woodmasoni*. With a broader taxonomic sample, efforts should be made to standardize the description so that the determination of size is relative to other observable features of the dentary in the same individual. Within our sample, only *U. rubromaculata* has teeth that are enlarged relative to those found in comparable specimens of *U. woodmasoni*. All other specimens exhibit state 0. Note that unlike the condition with the maxillary teeth, the enlargement of the teeth on the dentary does not appear to be coupled with a reduction in number of teeth.
